# Reduced N_2_ fixation in the tropical Atlantic Ocean during the warm late Pliocene

**DOI:** 10.1038/s41467-026-75846-4

**Published:** 2026-08-05

**Authors:** Maayan Yehudai, Jesse R. Farmer, Marietta Straub, Anja S. Studer, Lukas Oesch, Roger C. Creel, Ralf Schiebel, Alexandra Auderset, Kira Lawrence, Nazik Ogretmen, Gerald H. Haug, Daniel M. Sigman, Alfredo Martínez-García

**Affiliations:** 1 Climate Geochemistry, Max-Planck Institute for Chemistry, Mainz, Germany; 2 Department of Earth and Planetary Sciences, Weizmann Institute of Science, Rehovot, Israel; 3 School for the Environment, University of Massachusetts Boston, Boston, MA USA; 4 Institute of Radiation Physics, Lausanne University Hospital and University of Lausanne, Lausanne, Switzerland; 5 Department of Environmental Sciences, University of Basel, Basel, Switzerland; 6 Geological Institute, Department of Earth Sciences, ETH Zürich, Zürich, Switzerland; 7 Department of Geology & Geophysics, Texas A&M University, College Station, TX USA; 8 School of Ocean and Earth Science, National Oceanography Centre Southampton, University of Southampton, Southampton, UK; 9 Department of Geology and Environmental Geosciences, Lafayette College, Easton, PA USA; 10 Department of Environmental Conservation, University of Massachusetts, Amherst, MA USA; 11 Center for Marine Sciences, University of Algarve, Faro, Portugal; 12 Portuguese Institute for the Ocean and Atmosphere, Algés, Portugal; 13 Department of Geosciences, Princeton University, Princeton, NJ USA; 14 Present Address: Aarhus Institute of Advanced Studies, Aarhus University, Aarhus, Denmark

**Keywords:** Element cycles, Palaeoceanography, Palaeoclimate

## Abstract

Water column denitrification, a major oceanic nitrogen (N) sink, decreased under past warmer-than-present climates, but the response of N_2_ fixation, the main oceanic N source, remains unresolved. Here we address this gap using foraminifera-bound N isotope records from the tropical Atlantic spanning the warm late Pliocene, a geological analog for projected 21^st^ century warming. Our records show that tropical North Atlantic N_2_ fixation was reduced during the late Pliocene and had increased markedly by the late Pleistocene, likely due to an increase in water column denitrification, a process that globally engenders N-poor, phosphorus (P)-bearing surface waters. After ~ 2.8 million years ago, as glaciations intensified, our records show that obliquity-paced variations in the intensity of N_2_ fixation emerged, likely reflecting sea-level controlled changes in continental shelf area and benthic denitrification in the western tropical Atlantic, again through the generation of N-poor, P-bearing surface waters. These findings show that N_2_ fixation responds to the ocean’s N loss processes both over millions of years and on orbital timescales, suggesting that the global marine fixed N budget may rebalance under warmer climates by reducing both water column denitrification and N_2_ fixation.

## Abstract (web summary)

Tropical Atlantic nitrogen isotope records show that N₂ fixation was reduced during the warm late Pliocene due to decreased global water column denitrification. It increased as Earth cooled into the Pleistocene, with sea-level changes modulating glacial-interglacial fixation rates.

## Introduction

Nitrogen (N) is a key element in ocean biogeochemistry due to its role as a limiting nutrient for biological productivity and its associated influence on the carbon (C) cycle and the oceanic dissolved oxygen distribution (e.g., 1). A unique characteristic of the N cycle is that the oceanic inventory of biologically available (“fixed”) N is dominantly controlled by biological processes^1^. Fixation of atmospheric N_2_ by cyanobacteria, the major N_2_-fixing microorganisms in the open ocean, produces most of the bioavailable N in the ocean and thus plays a vital role in sustaining marine life^2^. Denitrification is an anaerobic microbial process that removes fixed N from the ocean by converting biologically available N back into N_2_^3^. The removal of fixed N by denitrification generates excess Phosphorus (P), i.e., P that exceeds phytoplankton requirements based on the Redfield stoichiometric ratio (N/P ≈ 16/1). With the supply of excess P to the euphotic zone, nutrient consumption by phytoplankton leads to N-deplete but P-bearing surface waters, which encourages N_2_ fixation, providing a mechanism that couples N inputs to losses in the ocean^4–9^.

Global warming has the potential to perturb components of the marine N cycle, including the sources and sinks of fixed N^10^. However, the interactions between N_2_ fixation and denitrification, as well as their spatiotemporal response to a warmer climate, are poorly understood^6,11–13^. Past climate analogs can provide important information about changes in N cycle dynamics in a warmer world. The Middle Pliocene Warm Period (~ 3.3 to ~ 3.0 million years ago; Ma) is considered as a potential analogue for 21st century Earth^14^. Pliocene global average temperatures were ~2–4 °C warmer than today, with atmospheric CO_2_ levels (hereafter, pCO_2_) and a continental configuration similar to present^15,16^. During the Pliocene-Pleistocene Transition (PPT; defined here as ~3.2–2.6 Ma), Earth’s climate cooled and became more variable^17^ as Northern Hemispheric glaciations intensified (iNHG; defined here as occurring at ~2.8 Ma). During this interval, pCO_2_ decreased^15,16,18–20^ and ocean circulation and spatial productivity patterns reorganized^17,21,22^. Given this context, N cycle dynamics may have shifted as well. While changes in the oceanic N inventory have been proposed to modulate the biological pump and, in turn, atmospheric pCO_2_ levels^23–25^, their contribution to late Pliocene climate change and the PPT remains poorly constrained, and will depend on the relationship between N₂ fixation and denitrification.

Nitrogen isotopes are a powerful tool for studying changes in the modern and past marine N cycle^26–28^. N_2_ fixation in surface waters of the ocean adds newly fixed N with a δ^15^N of ~ −1‰^29^ (where δ^15^N = (^15^N/^14^N)_sample_/(^15^N/^14^N)_reference_ −1, with atmospheric N_2_ as the reference). The newly fixed N is exported in sinking matter and nitrified in the subsurface, lowering the δ¹⁵N of the shallow pycnocline nitrate pool^30^. Denitrification occurs mainly in oxygen-deficient zones (ODZs) of the oceanic water column and in suboxic sediment porewaters on marine continental shelves^1,30^. Within the ODZs, water column denitrification preferentially removes ^14^N-nitrate, raising the residual nitrate δ^15^N to ~14–20‰^31^. This δ^15^N elevation is transported throughout the ocean by circulation and export production^32^, ultimately raising the δ^15^N of global mean ocean nitrate above that of oceanic N inputs, which are dominated by N_2_ fixation^30,33^. By contrast, benthic/sedimentary denitrification, which is probably the ocean’s greatest fixed N loss pathway, has little isotopic effect on the mean oceanic nitrate δ^15^N as it typically consumes nitrate to completion^34,35^. Accordingly, the global mean δ^15^N of ocean nitrate is sensitive to the ratio of denitrification occurring in the water column vs. the sediments, with proportionally more water column denitrification translating to a higher mean ocean δ^15^N^36,37^. Both denitrification pathways also generate excess P relative to N, which may in turn encourage N_2_ fixation as described above. However, because of the high isotope effect of water column denitrification and the minimal isotope effect of sedimentary denitrification, the coupling of each to N_2_ fixation has distinct effects on global δ^15^N mean value of, and regional pycnocline gradients in, nitrate δ^15^N^30,38^.

Existing studies of changes in the Pliocene N cycle were based on bulk sediment δ^15^N^39,40^. However, bulk sediment δ^15^N may be altered by diagenetic effects and the contribution of exogenous N δ^15^N^4,41–48^. Foraminifera-bound δ^15^N (hereafter FB-δ^15^N) reliably records the δ^15^N of the nitrate supplied to the euphotic zone^26,27^, in part because the fossil test protects the bound organic matter from diagenesis and external N contamination^49^. A recent study based on both bulk sediment δ^15^N and FB-δ^15^N showed that water column denitrification in the Eastern tropical Pacific was lower during the warmer conditions of the Pliocene, and increased as global climate cooled into the Pleistocene^50^. This result aligns with additional data indicating that global water column denitrification rates were lower during past warmer climatic periods^40,47,48,50–52^. As possible explanations for reduced water column denitrification during warmer periods, weaker low-latitude winds may have suppressed upwelling and reduced subsurface oxygen demand, while invigorated Southern Ocean deep-water ventilation may have increased deep-ocean oxygen supply^47^. However, the potential consequences of these changes for N_2_ fixation and their implications for the feedbacks that shape the global ocean N budget remain unclear.

Benthic denitrification rates have been suggested to vary with continental shelf area changes and therefore respond to glacial-interglacial sea-level fluctuations^5,53^, thus influencing oceanic nitrate δ¹⁵N indirectly. Regional pycnocline δ^15^N values may deviate from the global mean pycnocline δ^15^N, currently ~ 6.25‰^54,55^, for example due to local isotopic modification by water-column denitrification in ODZs and N_2_ fixation in oligotrophic gyres. Given the large changes in continental shelf area that accompanied the expanded glacial-interglacial sea level variations after the iNHG^5,53^, benthic denitrification may have changed substantially as well.

**Fig. 1:**
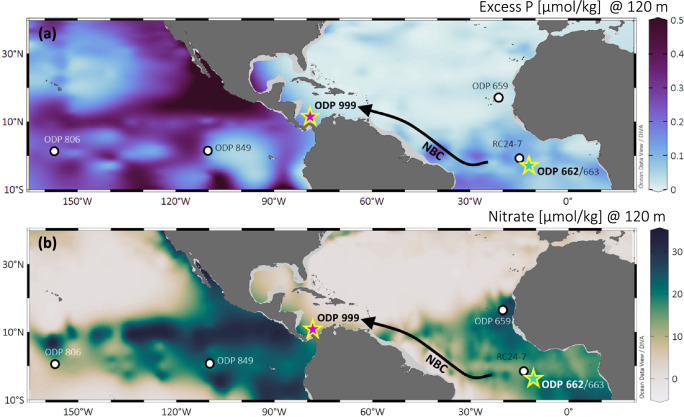
Core locations and shallow subsurface nutrient characteristics. **a** annual average excess P at 120 m water depth, and **b** annual average nitrate concentrations, both at 120 m depth. Maps were generated using Ocean Data View (ODV)^89^. Excess P is derived from annual average of phosphate and nitrate data (Excess *P* = [PO_4_^3−^]-[NO_3_^−^]/16, with units of μmol/l)^8^. Stars mark locations of Ocean Drilling Program (ODP) Sites 999 and ODP 662, from which this study’s data are presented. Other cores referenced in the text are marked with circles. ODP Sites 806 and 849 are located in the West and East Equatorial Pacific and represent changes in Eastern Pacific water column denitrification driven by the strength of the Oxygen Deficient Zone (ODZ)^50^. Alkenone mass accumulation rates (MAR) data from ODP 662^22^ and *F. profunda* percentage data from core RC24-7^65^ are used to reconstruct changes in Eastern Equatorial Atlantic (EEA) upwelling during the Pliocene Pleistocene Transition (PPT) and over the last glacial cycle, respectively. Also shown are sites with Plio-Pleistocene dust flux data (ODP Sites 659^61^, 662^90^, and 663^60^). Shallow subsurface nitrate is higher at the EEA Site 662 and lower at the Western Tropical Atlantic (WTA) Site 999, reflecting the east-west nutrient supply gradient. The black arrow represents the Northern Brazil Current (NBC), which today transfers excess P-rich waters from the EEA upwelling regions into the WTA^63^.

In this work, we investigate the Plio-Pleistocene history of N_2_ fixation by generating a new, high-resolution FB-δ^15^N record spanning the mid-late Pliocene and into the early Pleistocene (~ 3.6 to ~2 Ma) based on measurements of two foraminifera species (see “Methods”) from the western tropical Atlantic (WTA) Ocean Drilling Program (ODP) Site 999 (12°45’N, 78°44’W; 2828 m; Figs. 1 and S1; “Methods”). ODP Site 999 is located in the tropical North Atlantic, which appears to host nearly 90% of the Atlantic basin’s N₂ fixation^7^, and has previously been shown to record orbitally paced variations in N_2_ fixation over the last glacial cycle^4,44^. We compare our WTA FB-δ^15^N record with new high resolution data from the eastern equatorial Atlantic (EEA) ODP Site 662 (1°23.41’S, 11°44.35’W, 3832 m; Figs. 1, S1, S2, and S4; “Methods”), where FB-δ^15^N changes reflect variations in the δ^15^N of nitrate in the main pycnocline that underlies the Atlantic subtropical gyres^53^ (see “Methods” section for further details). Site 999’s record spans more than a million years (~ 2.0–3.6 Ma) at 4–5 kyr resolution, with Site 662 covering the interval ~2.5–2.9 Ma, enabling the evaluation of both long-term and orbitally paced N_2_ fixation and pycnocline dynamics in a warmer-than-present climate context.

## Results

**Fig. 2:**
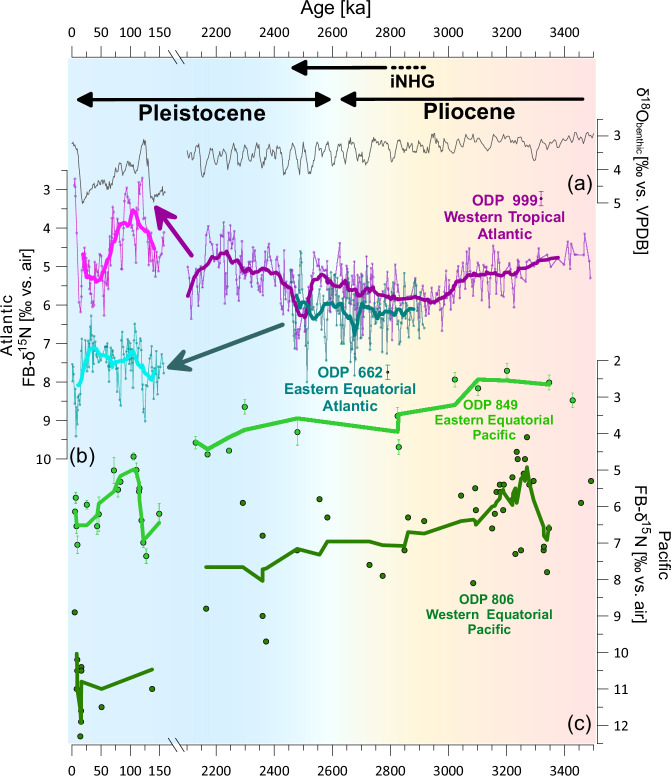
Foraminifera-bound (FB)-δ^15^N from Atlantic ODP Sites 999 and 662 and their relationship to changes in Pacific FB-δ^15^N. **a** benthic δ^18^O data from LR04 benthic δ^18^O stack (gray)^73^. **b** FB-δ^15^N from ODP Site 999 (this study in purple and ref. ^4^. in pink, derived from *T. sacculifer*) in the Caribbean Sea at the Western Tropical Atlantic (WTA) and ODP Site 662 (this study in teal and ref. ^53^ in light teal, derived from *T. sacculifer* and *N. dutertrei*) from the Eastern Equatorial Atlantic (EEA). The representative average standard uncertainty for both records is shown next to the site name. The thick lines mark the weighted average with a 21-point window width (~ 105 kyr for the Pliocene Pleistocene Transition (PPT) records and ~26 kyr for the last 160 ka record). **c** FB-δ^15^N records from Pacific ODP Sites 849^50^ (light green, thick line marks the weighted average with a 3-point window) and 806^50^ (dark green, thick line marks the weighted average with a 3-point window for the PPT and 5-point window for the last 160 kyr). The dashed black horizontal arrow represents the intensification of Northern Hemispheric Glaciation (iNHG) starting at ~2.8 Ma. The thick purple, teal and light and dark green arrows show the direction of FB-δ^15^N change between the PPT and the Late Pleistocene. Background shading indicates long-term cooling since the PPT. Note that higher isotope values are plotted downward in all panels.

Our FB-δ^15^N records from the WTA (Site 999A; Supplementary Data 1) and EEA (Site 662; Supplementary Data 2) capture the gradual evolution of conditions over the Plio-Pleistocene as well as changes over glacial-interglacial timescales. WTA FB-δ^15^N values of the surface-dwelling foraminifera *Trilobatus sacculifer* and *Globerginoides ruber* (see “Methods”) are elevated across the PPT compared to modern ocean measurements^9^, coretop sediments of Holocene age^26^ and time series records of the last 160 kyr^4^. As we observe no significant difference between *G. ruber* and *T. sacculifer* FB-δ^15^N variation in the WTA (“Methods” and Fig. S2), our measurements focused on *T. sacculifer*. During the PPT, *T. sacculifer* FB*-*δ^15^N averages 5.4 ± 0.3 (1σ)‰ compared to 4.5 ± 0.9 (1σ)‰ during the last 160 kyr (Welch’s *t*-test, *p* « 0.001; Fig. 2a). PPT *T. sacculifer* N content (Fig. S3) in the WTA varies between 2.6 and 5.6 μmol/mg, with an average of 3.9 ± 0.4 μmol/mg, and does not correlate with FB-δ^15^N, consistent with a lack of an isotopically fractionating diagenetic change in foraminifera-bound N (Fig. S4).

**Fig. 3:**
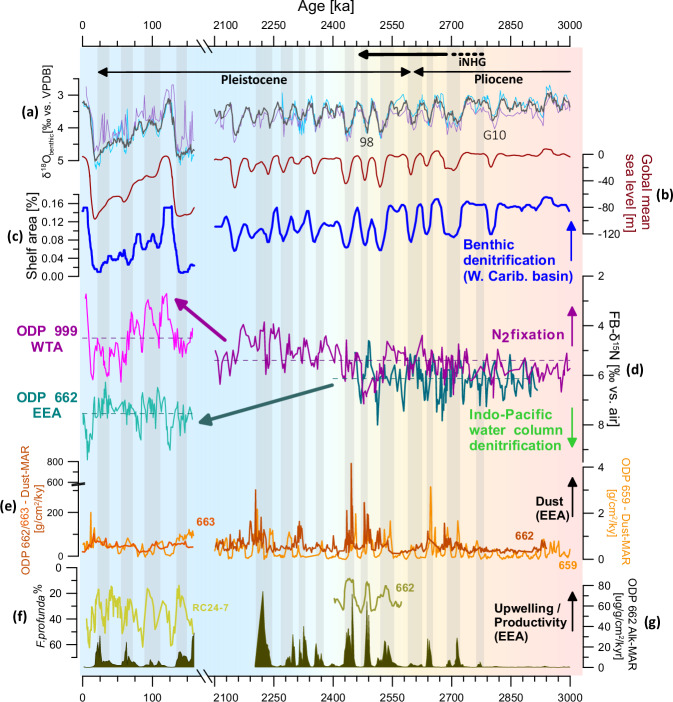
Comparison of the foraminifera-bound (FB)-δ^15^N data from ODP Sites 999 and 662 to Atlantic climate- and ocean-related records throughout the Pliocene-Pleistocene Transition (PPT) and over the Late Pleistocene. **a** benthic δ^18^O data from ODP 999 and 662 (violet and blue, respectively)^73^ plotted with the LR04 benthic δ^18^O stack (gray)^73^. Also noted here are Marine Isotopic Stages (MIS) G10, associated with the ﻿intensification of Northern Hemispheric Glaciation (iNHG), and MIS 98, associated with the early Pleistocene, where the FB-δ^15^N values at Site 999 are highest throughout the PPT record. **b** Global mean sea level is plotted in dark brown^66^ and is compared to (**c**) modelled changes in shelf area percentage relative to basin size in the Western Caribbean basin (W. Carib.; see main text, “Methods” and Fig. S12 for more details). **d** FB-δ^15^N records from ODP Sites 999 and 662 as described in Fig. 2. Purple diagonal arrow indicates the increase in N_2_ fixation between the PPT and the past 160 kyr. The diagonal teal arrow indicates the long-term increase in the pycnocline δ^15^N value as recorded at Site 662 between the Pliocene and the past 160 kyr. The purple/green vertical arrows represent the expected changes to FB-δ^15^N from higher N_2_ fixation and increased Plio-Pleistocene global water column denitrification^50^, respectively. The horizontal dashed lines indicate the average FB-δ^15^N values for the respective periods. **e** Dust input inferred from terrigenous mass accumulation rate (MAR) records from Eastern Atlantic ODP Sites 659^52^, 662^77^, and 663^75^ in orange, dark brown and light brown, respectively. **f** *F. profunda* abundance [%] data, which are interpreted to represent nutricline/pycnocline depth, from ODP Sites 662 (olive; 23) and RC24-7 (light green; 66). **g** Alkenone MAR from ODP 662 [μg/cm^2^/kyr] as a proxy for productivity (dark olive; 22). The grey vertical bands represent glacial periods corresponding with higher alkenone MAR. Background shading indicates long-term cooling since the PPT.

**Fig. 4:**
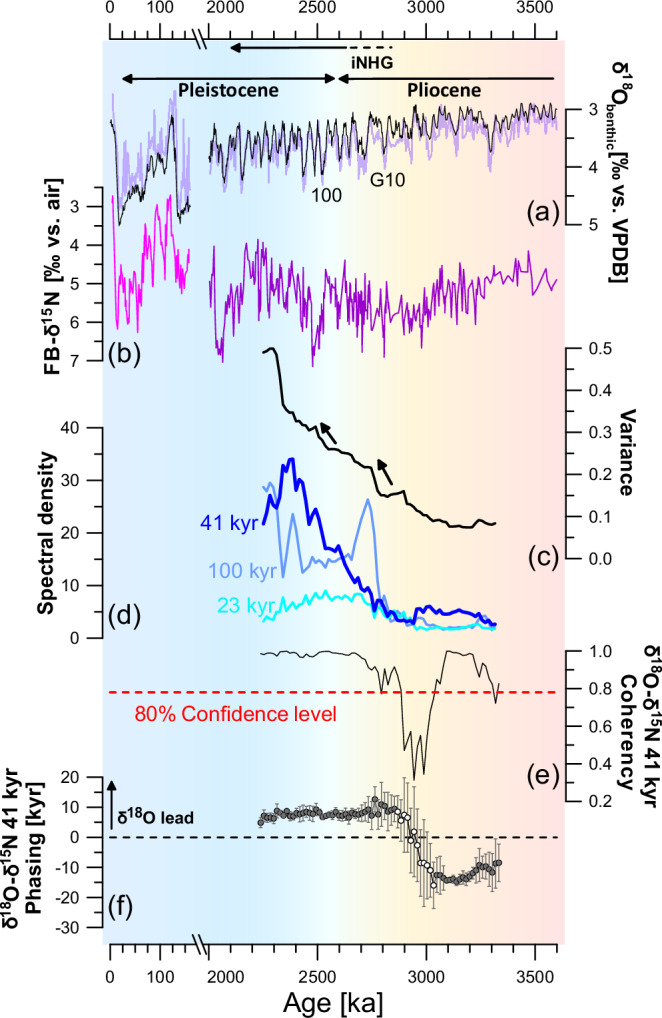
Foraminifera-bound (FB)-δ^15^N and continuous spectral analysis results from ODP Site 999. **a** Benthic δ^18^O data from ODP 999 (light purple; 68) and the LR04 benthic stack (black; 68). **b** FB-δ^15^N data from ODP 999 (this study and ref. ^38^) (note the reversed *y*-axis to indicate increase in N_2_ fixation upwards). **c** FB-δ^15^N variance analysis showing an increase ~ 2.8 Ma and also at ~ 2.6 Ma (black small arrows) coinciding with the beginning of the Pleistocene. **d** Continuous spectral density power for the three major orbital parameters (23 kyr precession, teal; 41 kyr obliquity, blue; 100 kyr eccentricity, light blue) with obliquity (41-kyr) showing the most persistent increase after intensification of the Northern Hemispheric Glaciation (iNHG) and Marine Isotopic Stage (MIS) G10 (~ 2.8 Ma). **e** Cross-coherency between the benthic δ^18^O and FB-δ^15^N records from ODP Site 999 on the 41-kyr period. The horizontal dashed red line represents the 80% confidence interval. **f** Phase relationship between the δ^18^O and δ^15^N records from ODP Site 999 for 41-kyr period, where upwards vertically means the δ^18^O record is leading δ^15^N as shown with the black arrow. The white-filled circles represent points where the coherency is lower than the 80% confidence interval. Spectral and cross-spectral analyses were conducted using ARAND software^83^. Background shading indicates long-term cooling since the Pliocene Pleistocene Transition (PPT).

The PPT WTA FB-δ^15^N record shows clear glacial-interglacial variability, with glacial values generally higher than interglacial values and ranging from around 6 to 7‰, with a notable increase during Marine Isotopic Stages (MIS) 100 and 98 (~ 2.46–2.55 Ma). Interglacial values decrease slightly after ~2.8 Ma. However, compared to interglacial FB-δ^15^N of the last 160 kyr, PPT interglacial values are elevated by ~2‰ (Fig. 3d). Using spectral analysis (“Methods”), the orbital variance and spectral density amplitude of WTA FB-δ^15^N begin to increase ~ 3 Ma (Fig. 4c), with sharper “jumps” during and after the iNHG at MIS G10 (~ 2.8 Ma) and MIS 100 (~ 2.5 Ma). In addition, a consistent increase in the dominance of the 41-kyr obliquity frequency band of FB-δ^15^N at Site 999 occurs after ~ 2.8 Ma (Fig. 4d). The importance of the 100-kyr eccentricity band also increases sharply during the iNHG; however, it remains inconsistent throughout the rest of the record. Finally, while the 23-kyr precession band within the ODP Site 999 δ^15^N record increases after the iNHG, this increase is smaller than the observed increase in obliquity spectral density. This is in contrast to the last 160 kyr, during which the FB-δ^15^N record from Site 999 shows pronounced dominance of the 23-kyr cyclicity (Figs. 2b and S9; ref. ^53^), overprinted on a longer timescale signal of higher FB-δ^15^N values during the last ice age and lower values during the Holocene and the Last Interglacial (Fig. 4; ref. ^4^).

At Site 662 in the EEA, we combine FB-δ^15^N from surface-dwelling *T. sacculifer*, whose dinoflagellate symbionts keep its δ^15^N close to the local nitrate source signal^44,56^, and thermocline-dwelling *N. dutertrei*, adjusted for a measured interspecies offset of 0.83 ± 0.47‰, to reconstruct pycnocline nitrate δ^15^N at Site 662 (“Methods”; Fig. S2). The Site 662 combined FB-δ^15^N record displays values over the mid-late Pliocene (averaging 6.17 ± 0.63 (1σ)‰) that are 0.5–2‰ lower than the last 160 kyr (averaging 7.54 ± 0.58 (1σ)‰; Welch’s *t*-test, *p* « 0.001; Fig. 3d; ref. ^53^).

During the PPT, Site 999 FB-δ^15^N was generally lower than at Site 662, but the inter-site average difference (0.5 ± 0.7 (1σ)‰) was far smaller than the average difference over the last 160 kyr (3.1 ± 1.1 (1σ)‰). While the influence of orbital frequencies is observed at both sites after the iNHG (Figs. 4 and S8; “Methods”), the FB-δ^15^N values from the two sites are either inversely correlated or uncorrelated. Similar FB-δ¹⁵N structural differences are observed over the last 160 kyr, with a strong precession-paced variability at Site 999 versus the absence of a clear precessional signal at Site 662^53^.

## Discussion

### Drivers of WTA N_2_ fixation since the Pliocene

We first evaluate global pycnocline δ^15^N change as reconstructed from Site 662 and then assess regional N_2_ fixation changes inferred from FB-δ^15^N Site 999 and its difference from that of Site 662. At Site 662, the lower FB-δ^15^N values seen across the PPT relative to the last 160 kyr indicate that the δ^15^N of pycnocline nitrate supplying the tropical Atlantic was lower during the warmer Pliocene and increased into the late Pleistocene. Lower Pliocene nitrate δ^15^N is consistent with reduced water column denitrification and/or enhanced sedimentary denitrification. N isotope evidence already exists for reduced global water column denitrification in the Pacific during the PPT (ODP Sites 806 and 849; Fig. 2; ref. ^50^), which would have decreased the δ^15^N of nitrate in the global mean pycnocline (Fig. 2b) and, thereby, FB-δ^15^N at Site 662. The δ^15^N of the global mean pycnocline nitrate value (~ 6.25‰ today; Fig. S1; refs. ^54,55^) represents the best value for calculating the global ocean’s N isotopic budget^55^, which is composed dominantly of N_2_ fixation and denitrification. Regions where the main pycnocline outcrops, such as at Site 662^53^, provide an opportunity to monitor its nitrate δ^15^N for comparison with records such as that of Site 999. In contrast to the 662 long-term observation, FB-δ^15^N values at Site 999 in the WTA were generally higher across the PPT relative to the last 160 kyr interglacials (Fig. 2a).

Both water column and sedimentary denitrification consume fixed N, but not P, thus generating the global supply of excess P. Reduced water column denitrification throughout the PPT, as documented in the Pacific sites^50^, is therefore consistent with reduced global supply of excess P, which would have impacted both the EEA and WTA regions, though not in the same manner. In the modern low latitude Atlantic, upwelling in the EEA outcrops nutrient-rich Southern Ocean-sourced intermediate and mode waters that carry signals from the Indo-Pacific denitrification processes^4,9^. The excess P generated by Indo-Pacific water column denitrification in these intermediate and mode water is upwelled in the low latitude Atlantic, including the EEA, and transported to the WTA through surface currents^9,57^.

**Fig. 5:**
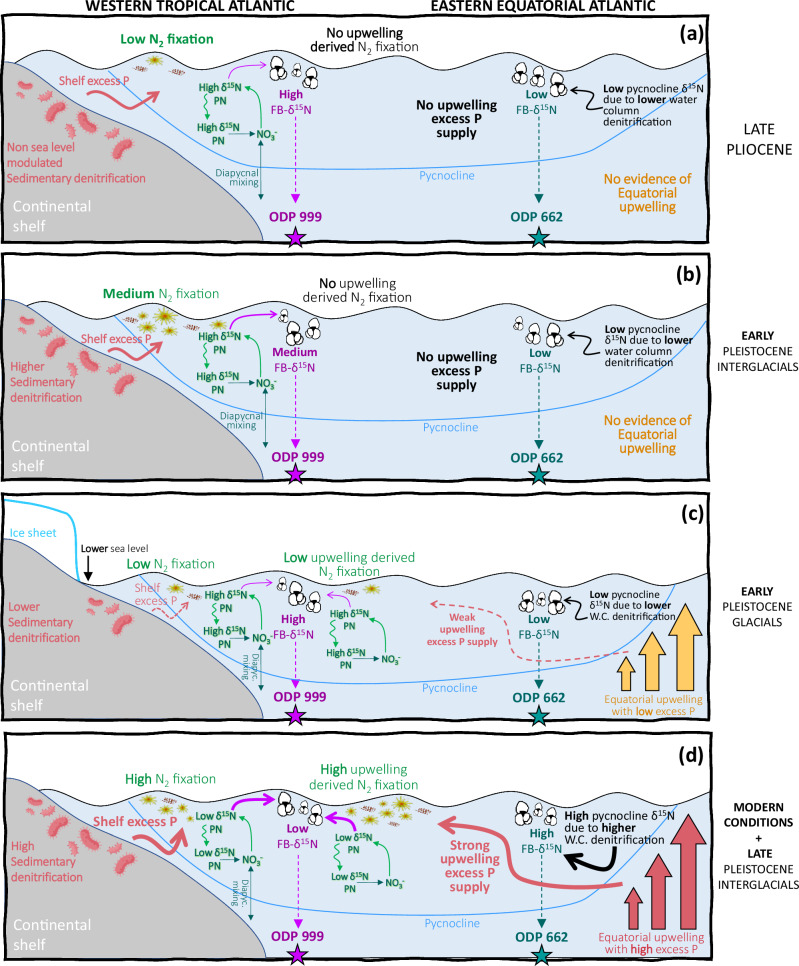
Schematic summary of changes in nitrogen (N) cycling and excess phosphorus (P) supply mechanisms across the Pliocene Pleistocene Transition (PPT), in the low latitude Atlantic. **a** **Warm Pliocene** (~3.6–2.8 Ma): Reduced water column (W.C.) denitrification in the Pacific lowered the excess P content and the δ^15^N value of the global average pycnocline, resulting in lower Foraminifera Bound (FB)-δ^15^N recorded at the Eastern Equatorial Atlantic (EEA, ODP Site 662) while decreasing upwelling-derived excess P supply to the Western Tropical Atlantic (WTA, ODP Site 999). This latter effect led to less N_2_ fixation and thus raised the δ^15^N of particulate nitrogen (PN) incorporated into planktic foraminifera in the WTA, reducing the east-to-west FB-δ¹⁵N gradient. **b** **Early Pleistocene interglacials** (after ~2.8 Ma): The ﻿intensification of Northern Hemispheric Glaciation (iNHG) introduced orbitally-paced sea-level fluctuations that repeatedly exposed and submerged continental shelves. Higher sea level during interglacials increased shelf area, promoting sedimentary denitrification and excess P supply to the WTA, resulting in higher N₂ fixation relative to glacials. **c** **Early Pleistocene glacials**: Despite intensified glacial equatorial upwelling in the EEA after the iNHG (orange upward arrows), weak Pacific water column denitrification limited excess P in upwelled waters (dashed peach arrow), thereby failing to drive a strong increase in N₂ fixation in the WTA. Moreover, lower sea levels further reduced shelf area and sedimentary denitrification, limiting regional excess P supply to the WTA and thus reducing N₂ fixation. **d** **Modern conditions and Late Pleistocene interglacials**: High water column denitrification increases the pycnocline’s excess P content and the pycnocline δ^15^N value that is then recorded in FB-δ^15^N in the EEA. Precession-paced upwelling (peach upward arrows) drives higher Atlantic N₂ fixation through the transport of excess P-rich waters from the EEA to the WTA (thick peach arrow). This decreases the δ^15^N of PN that is then incorporated into planktic foraminifera, lowering FB-δ^15^N in the WTA.

Because N_2_ fixation in the oligotrophic WTA is in part a response to the excess P supply from the global pycnocline^8,58^, a lower excess P reservoir during the PPT would be expected to suppress regional N_2_ fixation in the WTA. This expectation is supported by the higher WTA FB-δ^15^N interglacial values over the PPT relative to late Pleistocene interglacials (Fig. 2), which indicate reduced PPT levels of N_2_ fixation. We estimate the strength of N_2_ fixation in the tropical North Atlantic from the FB-δ^15^N difference between the WTA and EEA (ΔFB-δ^15^N = FB-δ^15^N_999_–FB-δ^15^N_662_; Fig. S9). This interpretation is based on the inference that Site 662 approximates the δ^15^N of the Atlantic-wide pycnocline nitrate supply^53^, with Site 999 indicating modification of the local pycnocline (and shallower) nitrate δ^15^N by the addition of newly fixed N carrying a low δ^15^N (Fig. 1). In this framework, lower ΔFB-δ^15^N during the warmer late Pliocene indicates lower N_2_ fixation rates, and higher ΔFB-δ^15^N into the Pleistocene indicates higher N_2_ fixation rates in the WTA (Fig. 5). Considered together, Site 662 indicates reduced global water-column denitrification, while the Site 999 record and its offset from Site 662 indicate reduced regional N_2_ fixation in the WTA (Fig. S9), consistent with the patterns described above.

The Pliocene-to-present increase in tropical interglacial Atlantic N_2_ fixation indicated by ΔFB-δ^15^N could be a response to dust-derived iron (e.g., refs. ^23,24^), might have had a connection to sea surface temperature (SST; ref. ^59^), and/or could have represented an excess-P-driven compensation for denitrification^5,44,53^. While the dust supply to the tropical Atlantic increased after ~2.75 Ma (Fig. 3e; refs. ^60–62^), higher dust input does not correlate with higher N_2_ fixation rates in our data (“Methods”; Fig. S10). The highest dust input is reconstructed during glacials, when the FB-δ^15^N values at Site 999 are higher, reflecting lower levels of N_2_ fixation. In addition, since the dust accumulation rates over the late Pliocene and during post-iNHG interglacials are similar (Fig. 3e), changes in dust supply alone cannot explain the overall lower rates of WTA N_2_ fixation over the PPT, nor the increase in N_2_ fixation after ~2.8 Ma. Regarding the role of temperature, SST and FB-δ^15^N display no clear relationship at Site 999 (Fig. S11), which rules out temperature as a direct contributor to either the long-term trend or the orbital scale variations in WTA FB-δ^15^N. Thus, we conclude that the lower N_2_ fixation rates observed in the WTA during the Pliocene compared to the present and to the last 160 kyr can only be explained by a reduction in the supply of excess P to the global pycnocline, most likely as a consequence of the reduction in water column denitrification in the Pliocene tropical Pacific.

### Orbital N_2_ fixation variability in the WTA during the early Pleistocene

The importance of excess P supply in driving N_2_ fixation changes in the North Atlantic over the last 160 kyr has been highlighted in previous studies^4,53^. However, our data suggest that, under the warmer climate state of the PPT, the variations in excess P supply to the WTA had a different origin than during the late Pleistocene. Today and over the late Pleistocene, excess P in the WTA is sourced from surface currents coming from EEA upwelling regions (Fig. 1) and is modulated by precession^4^. Upwelled waters in the EEA are relatively P-rich because these waters derive from intermediate and mode waters that were last ventilated in the Southern Ocean and incorporate Indo-Pacific nutrient signals, including excess P generated by water column denitrification in the Pacific ODZs (Fig. 1; refs. ^4,9,63^). Moreover, tropical and subtropical South Atlantic surface waters are characterized by significant excess P, possibly because of iron limitation of N2 fixation^64^. There are, thus, two sources of excess P—EEA upwelling and resident South Atlantic surface waters—that are carried into the WTA by the North Brazil Current (NBC, Fig. 1a). In the case of stronger trade winds, both EEA upwelling and NBC transport increase, supplying more of both excess P sources to the WTA.

Over the last 160 kyr, an equatorial Atlantic upwelling control on N_2_ fixation in the WTA is supported by relationships between FB-δ^15^N at Site 999 and variations in alkenone mass accumulation rates (MAR) and nannofossil assemblages from Site 662 (Fig. 3; refs. ^4,53,65^). To explore the nature of the relationship between EEA upwelling dynamics and WTA N_2_ fixation changes during the PPT, we compare our WTA and EEA records to other proxy records reflecting different mechanisms that may alter the supply of excess P to the WTA (Figs. 3 and S10–S12). At Site 662, glacial-interglacial alkenone MAR variations likely initiated with the iNHG (Fig. 3f, g) and reflect productivity variations driven by obliquity-controlled changes in nutrient upwelling^22^. Across the PPT, proxies such as alkenone MAR and the abundance of the thermocline-dwelling coccolithophore *Florisphaera profunda* at Site 662 (Fig. 3f, g) indicate that higher nutrient upwelling and productivity occurred during the glacials associated with obliquity minima^17^. According to the mechanism proposed for the last 160 kyr, increased nutrient upwelling in the EEA should enhance N_2_ fixation in the WTA by increasing excess P supply. However, our data show that N_2_ fixation in the WTA was lower during glacial periods than interglacials across the PPT, as indicated by the highest FB-δ^15^N values. In other words, the minima in the WTA FB-δ^15^N (Fig. 3d), which we interpret to represent higher N_2_ fixation, generally correspond with the minima in productivity at Site 662 (Fig. 3f, g), suggesting that orbital-scale variation in N_2_ fixation during the PPT was not driven by EEA upwelling changes as it has been over the last 160 kyr^4^ and is today^9^. The lack of response of WTA N_2_ fixation to equatorial upwelling changes across the PPT suggests that, during this period, the upwelled waters were not as excess P-rich as they are today. This is consistent with reduced Pacific water column denitrification during the late Pliocene, as compared to the late Pleistocene^39,40,47,50^.

Instead, we propose that glacial-interglacial sea-level change was the primary control on WTA N_2_ fixation during and after the iNHG. Benthic denitrification rates have been suggested to vary with continental shelf area changes and therefore respond to glacial-interglacial sea-level fluctuations^5,53^. During the PPT, as glacial sea levels declined after ~2.8 Ma, continental shelf exposure reduced regional benthic denitrification, limiting excess P supply to the WTA, whereas interglacial shelf submergence enhanced benthic denitrification and N_2_ fixation. At Site 999, a cross-spectral analysis of FB-δ^15^N and benthic δ^18^O, which should at least partially reflect sea-level driven changes, shows increasing coherency that starts at ~3.3 Ma and becomes persistent after ~2.8 Ma (Fig. 4e, f), supporting this mechanism. The observed lead of δ^18^O relative to FB-δ^15^N may be explained by (1) the δ^18^O signal from cooling that should lead ice sheet-driven sea level fall, and/or (2) a delay in the re-establishment of benthic denitrification following deglacial sea level rise (Fig. 5).

The conclusion that sea-level driven shelf submergence drove changes in tropical N_2_ fixation during the PPT is consistent with late Pleistocene studies from the South China Sea^5^ and sub tropical North Atlantic^53^, where regional shelf area changes due to sea-level fluctuations influenced excess P supply and N_2_ fixation (Fig. S12). To test the significance of these fluctuations at the WTA during the PPT, we modeled continental shelf area percentage changes in the west Caribbean basin by applying two recent Plio-Pleistocene global mean sea-level reconstructions^66,67^ to the PRISM4 Pliocene topography^68^. The modeled shelf area percentage in the west Caribbean basin remained stable before the iNHG, with fluctuations emerging after ~2.8 Ma that correspond to glacial FB-δ^15^N maxima at Site 999 (“Methods”, Figs. 3b, c, S12, and S13). Relative to the South China Sea, where shelf area percentage fluctuations have changed significantly since the Pliocene (Fig. S12d), WTA shelf area variability during the early Pleistocene glacials is not very different from that observed over the last 160 kyr. This reflects the steeper continental shelf structure in the WTA compared to other regions, such that, in comparison to the glacial intervals of the PPT, the lower sea levels of the Late Pleistocene glacial intervals would not have translated to substantially less shelf area or sedimentary denitrification. This observation can also explain the small differences observed in FB-δ^15^N between the glacials of the PPT and those of the last 160 kyr compared to the respective changes in interglacial FB-δ^15^N between these periods (Fig. 4). Combined with our sea-level simulations, these observations are consistent with benthic denitrification changes, through their influence on N_2_ fixation, as the main driver of the glacial FB-δ^15^N oscillations at Site 999 after ~2.8 Ma (Figs. S12 and S13, “Methods”).

#### Implications for Atlantic N cycling in a warmer ocean

This study provides a continuous, orbital-scale resolution record of N_2_ fixation dynamics in the low latitude Atlantic across the Plio-Pleistocene Transition. Our records highlight the mechanisms controlling N_2_ fixation in the WTA, a region accounting for a major fraction of basin-wide Atlantic N₂ fixation^7^. These results document both glacial-interglacial variability and longer-term trends in oceanic N cycling that have not been captured in the modern or Quaternary records (Fig. 5). These findings from the warm Pliocene provide potential insights into how future warming may alter global N_2_ fixation. Recent studies have shown the water column denitrification, and thus the extent of the ODZs of the ocean, were systematically reduced during warmer periods of the geological past, suggesting that a similar pattern may emerge in the future under global warming^47,48,50,51^. In this study, we show that North Atlantic N_2_ fixation was also reduced during the warm Pliocene period. We attribute this to a decrease in the supply of excess P to the global pycnocline as a consequence of the reduced water column denitrification in the Pacific basin. These findings suggest that, if prolonged global warming does cause a significant decrease in water column denitrification, N_2_ fixation will also decrease due to the lower concentrations of excess P in the global pycnocline. This mechanism would rebalance the natural N sources and sinks of the ocean, preventing dramatic changes in the ocean N reservoir.

In addition to these Pliocene-to-Pleistocene changes, starting at the iNHG, we observe a pronounced increase in obliquity-driven variability in N_2_ fixation in the WTA. We argue that sea-level changes during and after the iNHG modulated glacial benthic denitrification rates by exposing continental shelf areas in the WTA. The glacial shelf exposure reduced the supply of excess P to the WTA surface ocean, diminishing N_2_ fixation and increasing WTA FB-δ^15^N (Fig. 5b). This mechanism contrasts with late Quaternary and modern conditions, whereby N_2_ fixation is primarily driven by equatorial upwelling changes that transfer excess P waters to the North Atlantic^4,9,53^ (Fig. 5c). We suggest that the apparent lack of N_2_ fixation response to equatorial upwelling changes during the early Pleistocene (Fig. 5b) was the consequence of a lower excess P concentration in the global pycnocline prior to the Late Pleistocene. That is, although equatorial upwelling intensified after the iNHG, the upwelled water did not contain enough excess P to stimulate major N_2_ fixation changes in the North Atlantic. Thus, our results suggest that if water column denitrification decreases under protracted global warming, Atlantic N_2_ fixation may become decoupled from equatorial upwelling changes, with potential consequences for upper ocean ecosystems^9^.

## Methods

### Core locations and hydrographic settings

We analyzed samples from two ODP sites in the low-latitude Atlantic Ocean. ODP Site 999 Hole A (12° 45’ N, 78° 44’ W; 2828 m) is in the Caribbean Sea (Fig. 1). In the modern ocean, this region is oligotrophic, as surface waters feeding it originate from the subtropical gyres and are nutrient depleted (Fig. 1). The tropical North Atlantic hosts nearly 90% of the Atlantic basin’s N_2_ fixation^7^, sustained by diazotrophic activity in the Caribbean and tropical western North Atlantic^7,69,70^. Previous work suggests that most of the N_2_ fixation occurs in the oligotrophic surface ocean, i.e., the subtropical gyres^2,71^, which makes this site ideal for documenting changes in the Atlantic N_2_ fixation over time.

ODP Site 662 is located in the Guinea Basin in the EEA (1°23.41’S, 11°44.35’W, 3832 m) at the focus of equatorial surface water divergence (Fig. 1), where Ekman wind-driven upwelling brings nutrient-rich pycnocline waters to the surface^7,72^. Given the complete consumption of upwelled nitrate in the equatorial Atlantic surface ocean, FB-δ^15^N at Site 662 reflects the δ^15^N of pycnocline nitrate, which aligns with the global mean pycnocline nitrate δ^15^N of ~6.3‰^55^ and has remained stable across glacial-interglacial cycles^53^. The interpretive frameworks of the two sites, therefore, complement each other: Site 999 records regional changes in N₂ fixation, while Site 662 tracks changes in global pycnocline δ¹⁵N, controlled by the balance between water column denitrification and N₂ fixation^47^.

### Updated age models for ODP sites 662 and 999 A

The age models for both sites are based on alignment of benthic foraminifera δ^18^O to the δ^18^O benthic stack^73^. We updated the age model for Site 662 using δ^18^O values from the benthic stack as age tie points (Fig. S5 and Supplementary Data 3), with sample ages linearly interpolated between tie points using Python.

### Foraminifera-bound N isotope analysis

Foraminifera-bound δ^15^N was measured with the “persulfate-denitrifier” method^14,20,45,48,74^. Sediment samples from Sites 999 A and 662 were wet sieved through a 63-μm mesh and dried overnight in a clean oven at 40 °C. Two to three hundred individual foraminifera specimens were manually picked from the 250–425 μm size fraction. At Site 999 A, we picked *Trilobatus sacculifer* and another species *Globigerinoides ruber*, both commonly found in warm, oligotrophic surface waters of the tropical and subtropical oceans (Fig. S2)^75^. Since the FB-δ^15^N of both species exhibit a good correlation at Site 999 over the Early Pleistocene interval (Figs. S2 and S4), we focused on the FB-δ^15^N data from the more continuously measured *T. sacculifer* for this study.

At ODP Site 662, we picked specimens of *T. sacculifer* and non-spinose thermocline-dweller *Neogloboquadrina dutertrei*, the latter of which is typical for the tropical upwelling conditions characterizing Site 662. The FB-δ^15^N of *N. dutertrei* is typically higher than that of *T. sacculifer* given its absence of symbionts (see below). Based on the average matched sample FB-δ^15^N elevation of 0.83‰ in *N. dutertrei* relative to *T. sacculifer* at Site 662, 0.83 ± 0.47‰ was subtracted from the measured *N. dutertrei* FB-δ^15^N values to reconstruct the pycnocline nitrate δ^15^N values captured by *T. sacculifer* (FB-δ^15^N_TC_; Fig. S2).

For both sites, 3–5 mg of foraminifera tests were gently crushed and chemically treated to remove clay, external organic matter, and manganese coatings^44,47,51^. Subsequently, the shell fragments were dissolved in 4 N hydrochloric acid to release the organic N bound within the calcite. The organic N was then converted to nitrate by persulfate oxidation. The nitrate concentration of the solution was measured by chemiluminescence^76^, and an aliquot of the nitrate solution (5 nmol of N) was converted to nitrous oxide using a strain of denitrifying bacteria *Pseudomonas chlororaphis*^77^. The N isotopic composition of the N_2_O produced by the bacteria was measured with a custom-built continuous-flow system for N_2_O extraction and purification connected to a Thermo Scientific MAT253+ Isotope Ratio Mass Spectrometer^77–81^. Measurements were referenced to air N_2_ for δ^15^N using the nitrate reference standards IAEA-NO_3_ (δ^15^N = 4.7‰) and USGS-34 (δ^15^N = −1.8‰), and then corrected for the contribution of the oxidation procedural blank^81^. Triplicates of in-house calcitic and aragonitic biomineral standards (PO-1, *n* = 18 and LO-1, *n* = 12 and MF-1, *n* = 3) were included in each run to evaluate the long-term internal laboratory standard reproducibility^49,82^, which yielded values of 0.2–0.3‰ (1σ).

#### Spectral and cross-spectral data analysis

FB-δ^15^N values were processed using both the ARAND software^83^ and Pyleoclim python script^84^. Initially, all analyzed records were resampled and interpolated at a resolution of 4000–5000 years, depending on their average original sampling resolution. The δ^15^N records were then spectrally analyzed using Continuous Wavelet Transform to estimate the change in time series variance and dominances of orbital scale cycles. Cross-spectral coherences and phase relationship with other climatic records was also analyzed using both ARAND (Fig. 3 in the main text) and Cross Wavelet Analysis of Pyleoclim python script (Fig. S8).

#### FB-δ^15^N offset analysis between *G. ruber* and *T. sacculifer* at ODP Site 999

Based on previous approaches^4,26^, we divided the species-level FB-δ¹⁵N data into glacial and interglacial groups based on the LR04 glacial interglacial boundary ages^73^, to test whether the δ^15^N offset between *G. ruber* and *T. sacculifer* varies between climate states. For each group, we calculated linear regressions between the species and assessed the δ^15^N offset using Welch two-sample *t*-tests and bootstrap-derived confidence intervals (Supplementary Data 4). Both glacial and interglacial subsets show strong correlations between the two species (*r*^*2*^ = 0.7, *p* = 5.4 × 10^−15^ for glacials; *r*^*2*^ = 0.5, *p* = 1.6 × 10^−7^ for interglacials), indicating that relative changes in δ^15^N are closely tracked by both species. The mean offsets are −0.73‰ for glacials and −0.52‰ for interglacials, and while these offsets are statistically significant (*p* = 0.045 for both), the magnitude is small relative to the resolved δ^15^N variability in the ODP Site 999 record. The bootstrap analysis of the mean offset further confirms that the glacial-interglacial difference in mean offset is ~0.2‰. These results justify our use of the *T. sacculifer* δ^15^N record as representative for Site 999, while acknowledging the minor climate-dependent offset, which does not significantly impact the overall interpretation.

#### Calculation of pycnocline nitrate δ^15^N from ODP Site 662 FB-δ^15^N

Previous work on foraminifera-bound nitrogen isotopes^26,27^ indicates that FB-δ^15^N of *T. sacculifer* is 1–1.5‰ lower than that of the non-symbiotic thermocline-dwelling species *N. dutertrei*. This offset between the symbiotic species *T. sacculifer* and the non-symbiotic species *N. dutertrei* is most likely caused by the uptake of excreted low-δ^15^N ammonia by the symbionts and the subsequent internal N cycle to the host, which would lower the δ^15^N of the foraminifera organic N. FB-δ^15^N of *T. sacculifer* is thought to be very close to the δ^15^N of its food source and thus reflects the δ^15^N of supplied nitrate^26^. In contrast, modern measurements of the non-symbiotic thermocline-dwelling species *N. dutertrei* reveal a FB-δ^15^N elevation of around 1‰ with respect to the nitrate consumed in the euphotic zone^26,27^. In our data, FB-δ^15^N_*T. sacculifer*_ (Fig. S2) varies from 5 to 7.5‰ and FB-δ^15^N_*N. dutertrei*_ from 6‰ to 8‰, which indicates the δ^15^N of the nitrate supply to be between 5 and 7.5‰ and an offset between the two species of −0.5 to 2‰. The sample resolution of *T. sacculifer* FB-δ^15^N at Site 662 is limited due to sporadic appearance of the species. We therefore combined the two species’ FB-δ^15^N data by calculating the average of their δ^15^N (FB-δ^15^N_TC_; Fig. S2c). To calculate FB-δ^15^N_TC_, the FB-δ^15^N_*N. dutertrei*_ was first corrected by subtracting the average δ^15^N elevation of 0.83 ± 0.47‰ relative to FB-δ^15^N_*T. sacculifer*_ at sample depths where both species were measured (Fig. S2d). The combined FB-δ^15^N_TC_ dataset from Site 662 is therefore used in the main text to reflect pycnocline nitrate δ^15^N at this site. The N content for the species measured at Site 662 range from 3.21 to 5.01 μmol/mg with an average of 3.88 μmol/mg for *N. dutertrei*, and 3.41 to 5.02 μmol/mg with an average of 4.19 μmol/mg for *T. sacculifer*, both showing no correlation with FB-δ^15^N (Fig. S4).

#### Continental shelf area percentage calculation using past sea level modelling

To produce a first-order estimate of continental shelf area change in the west Caribbean, we apply the global mean sea level curve of Berends et al.^66^ to the PRISM4 Pliocene topography reconstructed by Dowsett et al. (Fig. S12 and ref. ^68^). This topography, constructed to represent the mid-Piacenzian (~ 3.0 Ma) and named PRISM4, accounts for dynamic topography and residual glacial isostatic adjustment signals in modern topography due to Pleistocene ice loading. In addition, we repeated the analysis using the recently published Plio-Pleistocene global mean sea-level reconstruction^67^ to assess the sensitivity of our results to the choice of sea-level simulation (Fig. S13). After merging global mean sea level and topography, we calculate the continental shelf area as the region at each time step extending from mean tide level to −150 m below sea level.

Beyond the correction included in PRISM4, we choose not to account for Pliocene glacial isostatic adjustment in our calculations. We justify this choice in several ways. First, while Pliocene to modern ice models for Antarctica are accessible (e.g., ref. ^85^), no such models are freely available for the Northern Hemisphere (sensu^66,86,87^), which hinders production of Glacial Isostatic Adjustment (GIA) models for this period. Second, although GIA exerts a spatially heterogenous influence on continental shelf area in the late Pleistocene (e.g., ref. ^53^), this influence was likely more muted at low- to mid-latitudes during the Pliocene due to the smaller size and loading footprint of the Northern Hemisphere ice sheets. Nevertheless, we acknowledge that some degree of GIA-related deformation may have occurred^86^, and our calculation should therefore be regarded as a first-order estimate.

Using the calculated shelf area and the global mean sea-level, we can then calculate the shelf area percentage with respect to the basin size, presented in Fig. 4 in the main text and Figs. S12 and S13. To test the sensitivity of these results to the choice of sea-level reconstruction, we repeated the shelf-area analysis using the recently published GMSL curve^67^. Although these recent sea level estimates yield lower glacial sea levels after the iNHG compared to earlier reconstructions, the pattern of enhanced variability after ~2.8 Ma is similar and is consistent with our initial interpretation (Fig. S13). However, since the variations in *glacial* sea level or shelf area percentage do not significantly change between the Pliocene and Pleistocene-Holocene interglacials (Fig. S12), the sea-level modulated benthic denitrification cannot explain the overall lower N_2_-fixation across the PPT at the WTA Site 999. Rather, this long-term change in N_2_ fixation is consistent with lower Pacific denitrification, as supported by the PPT-to-late-Pleistocene-Holocene EEA and WTA FB-δ^15^N differences (Fig. S9).

## Supplementary information

Supplementary Information

Description of Additional Supplementary Files

Supplementary Data 1

Supplementary Data 2

Supplementary Data 3

Supplementary Data 4

Peer Review file

## Data Availability

The foraminifera-bound nitrogen isotope data generated in this study (ODP Sites 999 and 662) have been submitted to the PANGAEA repository and can be found in the following links: Supplementary Dataset S1- ODP Site 999 FB-δ15N: 10.1594/PANGAEA.996039. Supplementary Dataset S2- ODP Site 662 FB-δ15N: 10.1594/PANGAEA.996038. Supplementary Dataset S3- ODP Site 662 age model LR04 tie points: 10.1594/PANGAEA.996036. The processed nitrogen isotope data generated in this study are also provided in the Supplementary Information Data file.

## Code availability

The code used in this study is available in the Zenodo repository (10.5281/zenodo.20929804)^88^.

## References

[1] Gruber, N. The marine nitrogen cycle: overview and challenges. *Nitrogen Mar. Environ*. 10.1016/B978-0-12-372522-6.00001-3 (2008).

[2] Wang, W. L., Moore, J. K., Martiny, A. C. & Primeau, F. W. Convergent estimates of marine nitrogen fixation. *Nature* **566**, 205–211 (2019). 30760914 10.1038/s41586-019-0911-2

[3] Gruber, N. & Galloway, J. N. An Earth-system perspective of the global nitrogen cycle. *Nature* **451**, 10–13 (2008). 10.1038/nature06592 18202647

[4] Straub, M. et al. Changes in North Atlantic nitrogen fixation controlled by ocean circulation. *Nature* **501**, 200–203 (2013). 23965620 10.1038/nature12397

[5] Ren, H. et al. Impact of glacial/interglacial sea level change on the ocean nitrogen cycle. *Proc. Natl. Acad. Sci. Usa.* **114**, E6759–E6766 (2017). 28760968 10.1073/pnas.1701315114 PMC5565415

[6] Sohm, J. A., Webb, E. A. & Capone, D. G. Emerging patterns of marine nitrogen fixation. *Nat. Rev. Microbiol.* **9**, 499–508 (2011). 21677685 10.1038/nrmicro2594

[7] Marconi, D. et al. Tropical dominance of N2 fixation in the North Atlantic Ocean. *Glob. Biogeochem. Cycles* **31**, 1608–1623 (2017).

[8] Deutsch, C., Sarmiento, J. L., Sigman, D. M., Gruber, N. & Dunne, J. P. Spatial coupling of nitrogen inputs and losses in the ocean. *Nature* **445**, 163–167 (2007). 17215838 10.1038/nature05392

[9] Jung, J. et al. Equatorial upwelling of phosphorus drives Atlantic N_2_ fixation and Sargassum blooms. *Nat. Geosci*. 10.1038/s41561-025-01812-2 (2025).

[10] Gruber, N. & Galloway, J. N. An Earth-system perspective of the global nitrogen cycle. *Nature* **451**, 293–296 (2008). 18202647 10.1038/nature06592

[11] Zehr, J. P. & Capone, D. G. Changing perspectives in marine nitrogen fixation. *Science* **80**, 368 (2020). 10.1126/science.aay9514 32409447

[12] Barcelos, E. et al. Effect of rising atmospheric carbon dioxide on the marine nitrogen fixer Trichodesmium. *Glob. Biogeochem. Cycles* **21**, 1–6 (2007).

[13] Shi, D., Kranz, S. A., Kim, J. M. & Morel, F. M. M. Ocean acidification slows nitrogen fixation and growth in the dominant diazotroph Trichodesmium under low-iron conditions. *Proc. Natl. Acad. Sci. USA*. **109**, E3094–E3100 (2012). 23071328 10.1073/pnas.1216012109 PMC3494951

[14] Burke, K. D. et al. Pliocene and Eocene provide best analogs for near-future climates. *Proc. Natl. Acad. Sci. USA*. **115**, 13288–13293 (2018). 30530685 10.1073/pnas.1809600115 PMC6310841

[15] Martınez-Botı, M. A. et al. Plio-Pleistocene climate sensitivity evaluated using high-resolution CO_2_ records. *Nature* **518**, 54 (2015). 10.1038/nature14145 25652996

[16] McClymont, E. L. et al. Lessons from a high-CO_2_ world: an ocean view from ~3 million years ago. *Clim* **16**, 1599–1615 (2020).

[17] Bolton, C. T. et al. Glacial-interglacial productivity changes recorded by alkenones and microfossils in late Pliocene eastern equatorial Pacific and Atlantic upwelling zones. *Earth Planet. Sci. Lett.* **295**, 401–411 (2010).

[18] Haug, G. H., Sigman, D. M., Tiedemann, R., Pedersen, T. F. & Sarnthein, M. Onset of permanent stratification in the subarctic Pacific Ocean. *Nature* **401**, 779–782 (1999).

[19] Shackleton, N. et al. Oxygen isotope calibration of the onset of ice-rafting and history of glaciation in the North Atlantic region. *Nature* **307**, 620–623 (1984).

[20] Sigman, D. M., Jaccard, S. L. & Haug, G. H. Polar ocean stratification in a cold climate. *Nature* **428**, 59–63 (2004). 14999278 10.1038/nature02357

[21] Raymo, M. E., Hodell, D. A. & Jansen, E. Response of deep ocean circulation to initiation of northern hemisphere glaciation. *Palaeogeogr. Palaeoclimatol. Palaeoecol.* **7**, 645–672 (1992).

[22] Lawrence, K. T. et al. Time-transgressive North Atlantic productivity changes upon Northern Hemisphere glaciation. *Paleoceanography* **28**, 740–751 (2013).

[23] Falkowski, P. G. Evolution of the nitrogen cycle and its influence on the biological sequestration of CO_2_ in the ocean. *Nature* **387**, 272–275 (1997).

[24] Broecker, W. S. & Henderson, G. M. The sequence of events surrounding termination II and their implications for the causes of glacial interglacial CO_2_changes. *Paleoceanography* **13**, 352–364 (1998).

[25] Tschitschko, B. et al. Rhizobia–diatom symbiosis fixes missing nitrogen in the ocean. *Nature* **630**, 899–904 (2024). 38723661 10.1038/s41586-024-07495-w PMC11208148

[26] Ren, H., Sigman, D. M., Thunell, R. C. & Prokopenko, M. G. Nitrogen isotopic composition of planktonic foraminifera from the modern ocean and recent sediments. *Limnol. Oceanogr.* **57**, 1011–1024 (2012).

[27] Smart, S. M. et al. Ground-truthing the planktic foraminifer-bound nitrogen isotope paleo-proxy in the Sargasso Sea. *Geochim. Cosmochim. Acta* **235**, 463–482 (2018).

[28] Jung, J. et al. Coral photosymbiosis on Mid-devonian reefs. *Nature* **636**, 647–653 (2024). 39443794 10.1038/s41586-024-08101-9 PMC11655356

[29] Knapp, A. N., Sigman, D. M. & Lipschultz, F. N isotopic composition of dissolved organic nitrogen and nitrate at the Bermuda Atlantic Time-series Study site. *Global Biogeochemical Cycles* **19**, GB1018 (2005).

[30] Sigman, D. M. & Fripiat, F. Nitrogen isotopes in the ocean. In *Encyclopedia of Ocean Sciences*10.1016/B978-0-12-409548-9.11605-7 (Elsevier Ltd., 2019).

[31] Cline, J. D. & Kaplan, I. R. Isotopic fractionation of dissolved nitrate during denitrification in the eastern tropical north pacific ocean. *Mar. Chem.* **3**, 271–299 (1975).

[32] Sigman, D. M., DiFiore, P. J., Hain, M. P., Deutsch, C. & Karl, D. M. Sinking organic matter spreads the nitrogen isotope signal of pelagic denitrification in the North Pacific. *Geophys. Res. Lett.* **36**, 1–5 (2009).

[33] Altabet, M. A. Constraints on oceanic N balance/imbalance from sedimentary 15N records. *Biogeosciences* **4**, 75–86 (2007).

[34] Brandes, J. A. & Devol, A. H. Isotopic fractionation of oxygen and nitrogen in coastal marine sediments. *Geochim. Cosmochim. Acta* **61**, 1793–1801 (1997).

[35] Lehmann, M. F. et al. The distribution of nitrate ^15^N/^14^N in marine sediments and the impact of benthic nitrogen loss on the isotopic composition of oceanic nitrate. *Geochim. Cosmochim. Acta* **71**, 5384–5404 (2007).

[36] Brandes, J. A. & Devol, A. H. A global marine-fixed nitrogen isotopic budget: implications for Holocene nitrogen cycling. *Glob. Biogeochem. Cycles* **16**, 67–14 (2002).

[37] Deutsch, C., Sigman, D. M., Thunell, R. C., Meckler, A. N. & Haug, G. H. Isotopic constraints on glacial/interglacial changes in the oceanic nitrogen budget. *Glob. Biogeochem. Cycles* **18**, 1–22 (2004).

[38] DeVries, T., Deutsch, C., Rafter, P. A. & Primeau, F. Marine denitrification rates determined from a global 3-D inverse model. *Biogeosciences* **10**, 2481–2496 (2013).

[39] Liu, Z., Altabet, M. A. & Herbert, T. D. Plio-Pleistocene denitrification in the eastern tropical North Pacific: intensification at 2.1 Ma. *Geochem., Geophys. Geosyst.* **9**, 1–14 (2008).

[40] Wang, X. T. et al. Oceanic nutrient rise and the late Miocene inception of Pacific oxygen-deficient zones. *Proc. Natl. Acad. Sci. USA*. **119**, 1–7 (2022). 10.1073/pnas.2204986119 PMC9659387 36322766

[41] Altabet, M. A., Francois, R., Murray, D. W. & Prell, W. L. Climate-related variations in denitrification in the Arabian Sea from sediment ^15^N/^14^N ratios. *Nature* **373**, 506–509 (1995).

[42] Altabet, M. A. & Francois, R. Sedimentary nitrogen isotopic ratio as a recorder for surface ocean nitrate utilization. *Glob. Biogeochem. Cycles* **8**, 103–116 (1994).

[43] Meckler, A. N. et al. Deglacial nitrogen isotope changes in the Gulf of Mexico: evidence from bulk sedimentary and foraminifera-bound nitrogen in Orca Basin sediments. *Paleoceanography* **26**, PA4216 (2011).

[44] Ren, H. et al. Foraminiferal isotope evidence of reduced nitrogen fixation in the ice age Atlantic Ocean. *Science* **244**, 244–248 (2009). 10.1126/science.1165787 19095896

[45] Robinson, R. S. et al. A review of nitrogen isotopic alteration in marine sediments. *Paleoceanography* **27**, PA4203 (2012).

[46] Studer, A. S. et al. Ice Age-Holocene similarity of foraminifera-bound nitrogen isotope ratios in the eastern equatorial Pacific. *Paleoceanogr. Paleoclimatol.* **36**, 1–16 (2021).

[47] Auderset, A. et al. Enhanced ocean oxygenation during Cenozoic warm periods. *Nature* **609**, 77–82 (2022). 36045236 10.1038/s41586-022-05017-0 PMC9433325

[48] Hess, A. V. et al. A well-oxygenated eastern tropical Pacific during the warm Miocene. *Nature* **619**, 521–525 (2023). 37380780 10.1038/s41586-023-06104-6

[49] Martínez-garcía, A. et al. Laboratory assessment of the impact of chemical oxidation, mineral dissolution, and heating on the nitrogen isotopic composition of fossil-bound organic matter geochemistry, geophysics, geosystems. *Geochem. Geophys. Geosyst.* **23**, e2022GC010396 (2022).

[50] Rafter, P. A. et al. Persistent eastern equatorial Pacific Ocean upwelling since the warm Pliocene. *Science* **390**, eads8720 (2025). 41037626 10.1126/science.ads8720

[51] Moretti, S. et al. Oxygen rise in the tropical upper ocean during the Paleocene-Eocene Thermal Maximum. *Science* **383**, 727–731 (2024). 38359106 10.1126/science.adh4893

[52] Rao, Z. C. et al. A nitrogen isotopic shift in fish otolith–bound organic matter during the Late Cretaceous. *Proc. Natl. Acad. Sci. USA*. **121**, e2322863121 (2024). 39074276 10.1073/pnas.2322863121 PMC11317583

[53] Auderset, A. et al. Sea level modulation of Atlantic nitrogen fixation over glacial cycles. *Paleoceanogr. Paleoclimatol.* **39**, 1–21 (2024).

[54] Fripiat, F. et al. Nitrogen isotopic constraints on nutrient transport to the upper ocean. *Nat. Geosci.* **14**, 855–861 (2021).

[55] Fripiat, F. et al. The impact of incomplete nutrient consumption in the Southern Ocean on global mean ocean nitrate δ15N. *Glob. Biogeochem. Cycles* **37**, 1–25 (2023).

[56] Auderset, A. et al. Effects of photosymbiosis and related processes on planktic foraminifera-bound nitrogen isotopes in South Atlantic sediments. *Biogeosciences* **22**, 1887–1905 (2025).

[57] Straub, M. et al. Nutrient conditions in the subpolar North Atlantic during the last glacial period reconstructed from foraminifera-bound nitrogen isotopes. *Nature* **28**, 79–90 (2013).

[58] Ward, B. A., Dutkiewicz, S., Moore, C. M. & Follows, M. J. Iron, phosphorus, and nitrogen supply ratios define the biogeography of nitrogen fixation. *Limnol. Oceanogr.* **58**, 2059–2075 (2013).

[59] Houlton, B. Z., Wang, Y. P., Vitousek, P. M. & Field, C. B. A unifying framework for dinitrogen fixation in the terrestrial biosphere. *Nature* **454**, 327–330 (2008). 18563086 10.1038/nature07028

[60] Demenocal, P. B. Plio-pleistocene African climate. *Science* **270**, 53–59 (1995). 7569951 10.1126/science.270.5233.53

[61] Crocker, A. J. et al. Geochemical response of the mid-depth Northeast Atlantic Ocean to freshwater input during Heinrich events 1 to 4. *Quat. Sci. Rev.* **151**, 236–254 (2016).

[62] Ruddiman, W. F., Raymo, M. E., Martinson, D. G., Clement, B. M. & Backman, J. Pleistocene evolution: northern hemisphere ice sheets and north Atlantic Ocean. *Paleoceanography* **4**, 353–412 (1989).

[63] Fratantoni, D. M., Johns, W. E., Townsend, T. L. & Hurlburt, H. E. Low-latitude circulation and mass transport pathways in a model of the tropical Atlantic Ocean. *J. Phys. Oceanogr.* **30**, 1944–1966 (2000).

[64] Moore, C. M. et al. Large-scale distribution of Atlantic nitrogen fixation controlled by iron availability. *Nat. Geosci.* **2**, 867–871 (2009).

[65] Molfino, B. & Mcintyre, A. Precessional forcing of nutricline dynamics in the equatorial Atlantic. *Science* **249**, 766–769 (1990). 17756790 10.1126/science.249.4970.766

[66] Berends, C. J., de Boer, B. & van de Wal, R. S.W. Reconstructing the evolution of ice sheets, sea level, and atmospheric CO_2_ during the past 3.6 million years. *Clim* **17**, 361–377 (2021).

[67] Clark, P. U. et al. Global mean sea level over the past 4.5 million years. *Science* **390**, eadv8389 (2025). 10.1126/science.adv8389 41100616

[68] Dowsett, H. et al. The PRISM4 (mid-Piacenzian) paleoenvironmental reconstruction. *Clim* **12**, 1519–1538 (2016).

[69] Knapp, A. N., DiFiore, P. J., Deutsch, C., Sigman, D. M. & Lipschultz, F. Nitrate isotopic composition between Bermuda and Puerto Rico: implications for N2 fixation in the Atlantic Ocean. *Glob. Biogeochem. Cycles* **22**, 1–14 (2008).

[70] Zehr, J. P. & Capone, D. G. Marine N2 fixation, global change and the future BT - marine nitrogen fixation. (eds. Zehr, J. P. & Capone, D. G.) 157–170 10.1007/978-3-030-67746-6_9 (Springer International Publishing, 2021).

[71] Shao, Z. et al. Global oceanic diazotroph database version 2 and elevated estimate of global oceanic N2 fixation. *Earth Syst. Sci. Data* **15**, 3673–3709 (2023).

[72] Brandt, P. et al. Physical processes and biological productivity in the upwelling regions of the tropical Atlantic. *Ocean Science* **19**, 581–601 (2023).

[73] Lisiecki, L. E. & Raymo, M. E. A Pliocene-Pleistocene stack of 57 globally distributed benthic d18O records. *Paleoceanography* **20**, 1–17 (2005).

[74] Jesse, R. et al. Early Pliocene shoaling of the Central American Seaway reconstructed from foraminifera-bound nitrogen and oxygen isotopes. *Paleoceanogr. Paleoclimatol.* **40**, e2024PA005043 (2025).

[75] Schiebel, R. & Hemleben, C. *Planktic Foraminifers in the Modern Ocean*, Vol. 358 (Springer, 2017).

[76] Braman, R. S. & Hendrix, S. A. Nanogram nitrite and nitrate determination in environmental and biological materials by vanadium(III) reduction with chemiluminescence detection. *Anal. Chem.* **61**, 2715–2718 (1989). 2619057 10.1021/ac00199a007

[77] Sigman, D. M. et al. A bacterial method for the nitrogen isotopic analysis of nitrate in seawater and freshwater. *Anal. Chem.* **73**, 4145–4153 (2001). 11569803 10.1021/ac010088e

[78] Moretti, S. et al. Analytical improvements and assessment of long-term performance of the oxidation-denitrifier method. *Rapid Commun. Mass Spectrom.* **38**, e9650 (2024). 10.1002/rcm.9650 38073197

[79] Casciotti, K. L., Sigman, D. M., Hastings, M. G., Bo, J. K. & Hilkert, A. Measurement of the oxygen isotopic composition of nitrate in seawater and freshwater using the denitrifier method. *Anal. Chem.* **74**, 4905–4912 (2002). 10.1021/ac020113w 12380811

[80] Weigand, M. A., Foriel, J., Barnett, B., Oleynik, S. & Sigman, D. M. Updates to instrumentation and protocols for isotopic analysis of nitrate by the denitrifier method. *Rapid Commun. Mass Spectrom.* **30**, 1365–1383 (2016). 27197029 10.1002/rcm.7570

[81] Böhlke, J. K., Mroczkowski, S. J. & Coplen, T. B. Oxygen isotopes in nitrate: new reference materials for 18O:17 O:16 O measurements and observations on nitrate-water equilibration. *Rapid Commun. Mass Spectrom.* **17**, 1835–1846 (2003). 12876683 10.1002/rcm.1123

[82] Leichliter, J. N. et al. Nitrogen isotopes in tooth enamel record diet and trophic level enrichment: results from a controlled feeding experiment. *Chem. Geol.* **563**, 120047 (2021).

[83] Howell, P., Pisias, N., Ballance, J., Baughman, J. & Ochs, L. *ARAND Time-Series Analysis Software* https://github.com/jesstierney/arand (2006).

[84] Khider, D. et al. Pyleoclim: paleoclimate timeseries analysis and visualization with Python paleoceanography and paleoclimatology. *Paleoceanogr. Paleoclimatol.*10.1029/2022PA004509 (2022).

[85] Pollard, D. & Deconto, R. M. Description of a hybrid ice sheet-shelf model, and application to Antarctica. *Geosci. Model Dev.* **5**, 1273–1295 (2012).

[86] Raymo, M. E., Mitrovica, J. X., O’Leary, M. J., DeConto, R. M. & Hearty, P. J. Departures from eustasy in Pliocene sea-level records. *Nat. Geosci.* **4**, 328–332 (2011).

[87] Willeit, M., Ganopolski, A., Calov, R. & Brovkin, V. Mid-Pleistocene transition in glacial cycles explained by declining CO2 and regolith removal. *Sci. Adv.* **5**, eaav7337 (2025). 10.1126/sciadv.aav7337 PMC6447376 30949580

[88] Yehudai, M. & Creel, R. C. *Code for: Reduced N₂ Fixation in the Tropical Atlantic Ocean During the Warm Late Pliocene*10.5281/zenodo.20929804 (2026). 10.1038/s41467-026-75846-4 PMC13448806 42562821

[89] Schlitzer, R. *Ocean Data View* https://odv.awi.de (2023).

[90] Ruddiman, W. F. & Janecek, T. R. Pliocene-Pleistocene biogenic and terrigenous fluxes at equatorial Atlantic Sites 662, 663, and 664. *Proc., Sci. Results, ODP, Leg. 108, East. Trop. Atl.* **108**, 211–240 (1989).

