# Supplementary material for: Reduced N2 fixation in the tropical Atlantic Ocean during the warm late Pliocene
Source: Nat Commun. 2026 Aug 5;17:7931. doi: 10.1038/s41467-026-75846-4 (PMC13448806; doi:10.1038/s41467-026-75846-4)
Supplement: Supplementary file 1 [file 41467_2026_75846_MOESM1_ESM.pdf]

Supplementary Information for

Reduced N_2 fixation in the Tropical Atlantic Ocean during the Warm Late Pliocene

Maayan Yehudai[1,2*], Jesse R. Farmer[3], Marietta Straub[1,4], Anja S. Studer[5], Lukas Oesch[6], Roger C. Creel[7], Ralf Schiebel[1], Alexandra Auderset[8], Kira Lawrence[9], Nazik Ogretmen[10,11], Gerald H. Haug[1,6], Daniel M. Sigman[12] and Alfredo Martínez-García[1*]

Maayan Yehudai
Email: m.yehudai@mpic.de

This PDF file includes:

Supplementary Figures S1 to S13
SI References

Other supporting materials for this manuscript include the following:

Supplementary Data 1 to 4

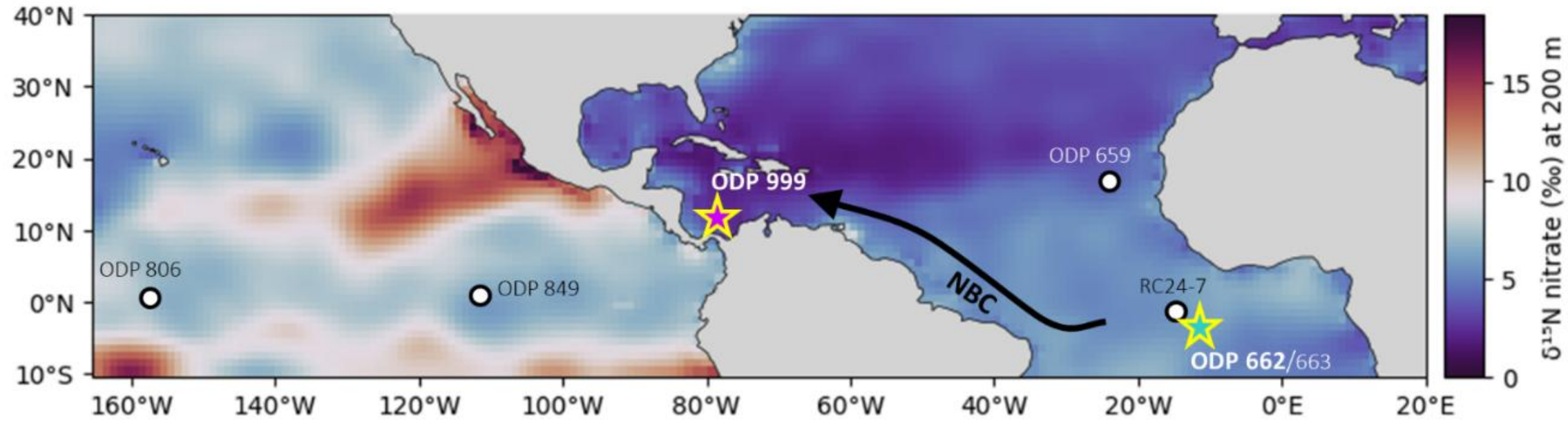

Fig. S1. Gridded δ^{15}N of nitrate (‰) at 200 m water depth predicted by an artificial-neural-network ensemble (1)**.** Circles and stars mark ODP sites discussed in the main text (Sites 849, 806, 662, 999, and 659. See further details in Fig. 1 caption of the main text). The modeled δ^{15}N illustrates regional gradients, with higher δ^{15}N values in eastern upwelling/ denitrification regions in the Pacific and Atlantic, and lower in the oligotrophic western basins. ODP Sites 662 and 999 highlight the modern east-west δ^{15}N contrast associated with the transfer of low-δ^{15}N, nitrate-depleted waters from the east to the west by the North Brazil Current (NBC).

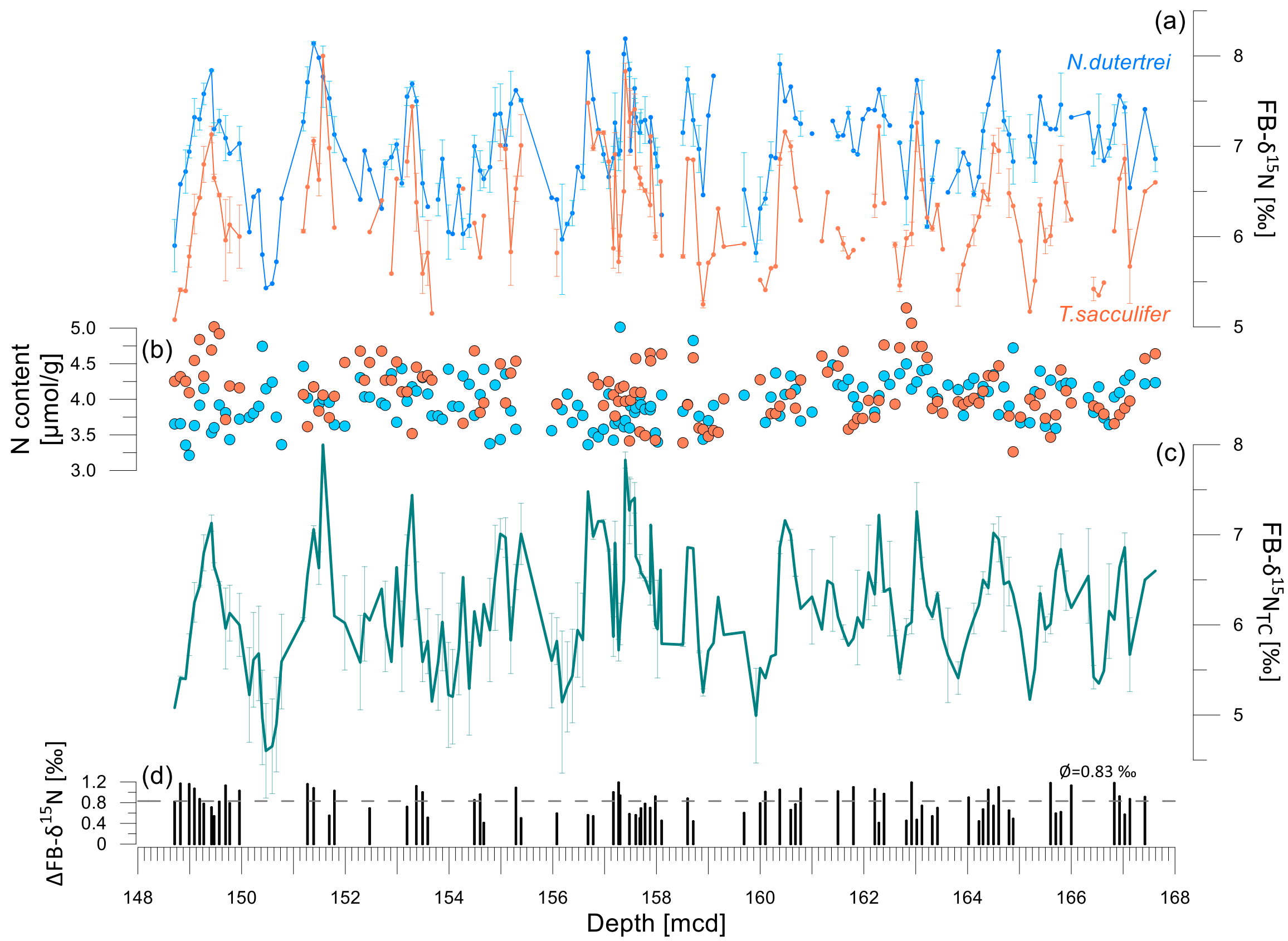

Fig. S2. FB-δ^{15}N from EEA Site ODP 662 during the PPT. (a) FB-δ^{15}N measured on the thermocline dwelling species *N.dutertrei* (blue) and on the euphotic zone dwelling species *T.sacculifer* (orange) (b) FB N content for both species. The average replicate standard deviation for each species' δ^{15}N is: *Ndut* = 0.16 ‰ *Tsacc*=0.17 ‰ (see Data Set S2). (c) Thermocline (TC) FB-δ^{15}N (teal). (d) Difference FB-$\delta^{15}N_{N.\ dutertrei}$ - FB-$\delta^{15}N_{T.\ sacculifer}$ at depths with measurements for both species, average=0.83 ± 0.47 ‰ (dashed horizontal line) was used for the calculation of the FB- $\delta^{15}N_{TC}$ (*see Materials and Methods section*).

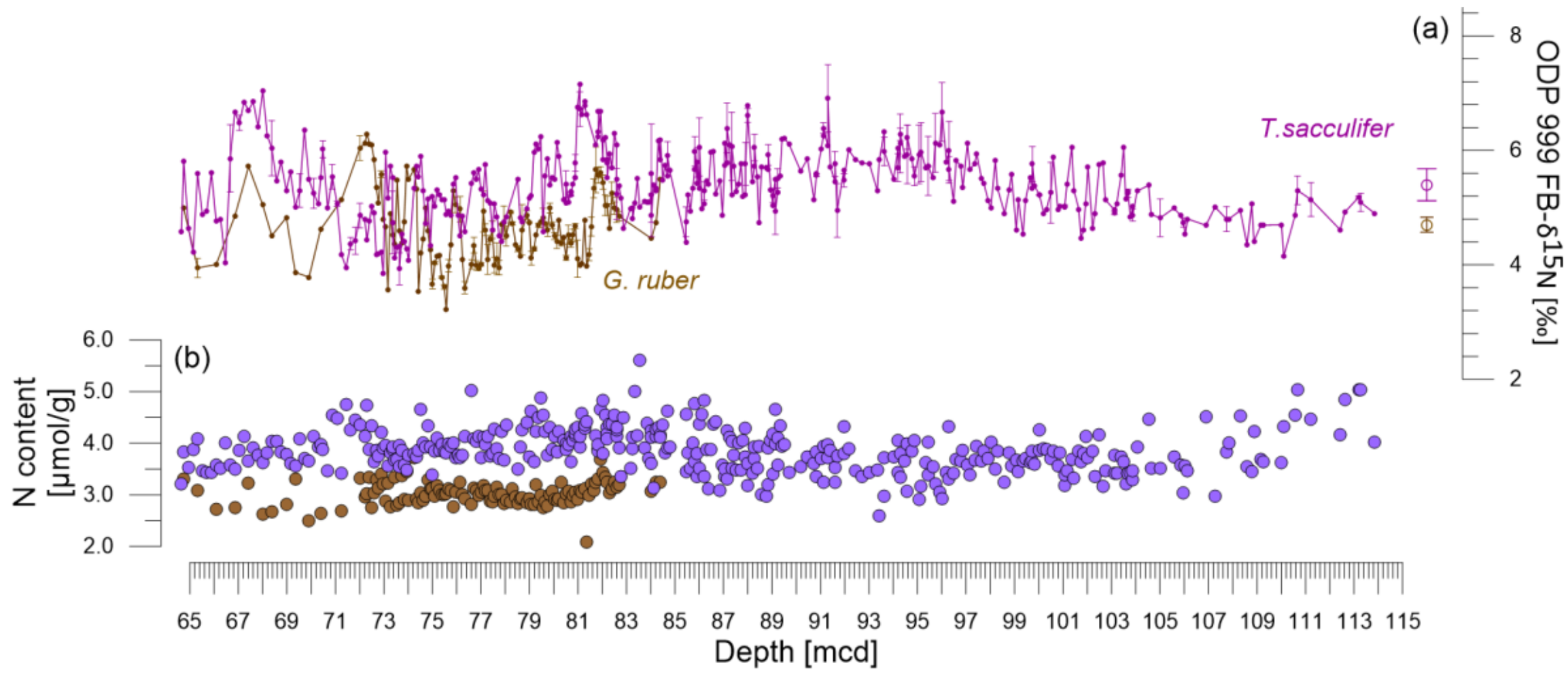

Fig. S3. FB-$\delta^{15}N$ from WTA Site ODP 999 during the PPT. (a) FB-$\delta^{15}N$ measured on the euphotic zone dwelling species *T. sacculifer* (purple) and *G. ruber* (brown) (b) N content for both species. The average replicate standard deviation for each species' $\delta^{15}N$ is: *Tsacc* = 0.28 ‰ *Gruber* = 0.13 ‰ (see Data Set S1).

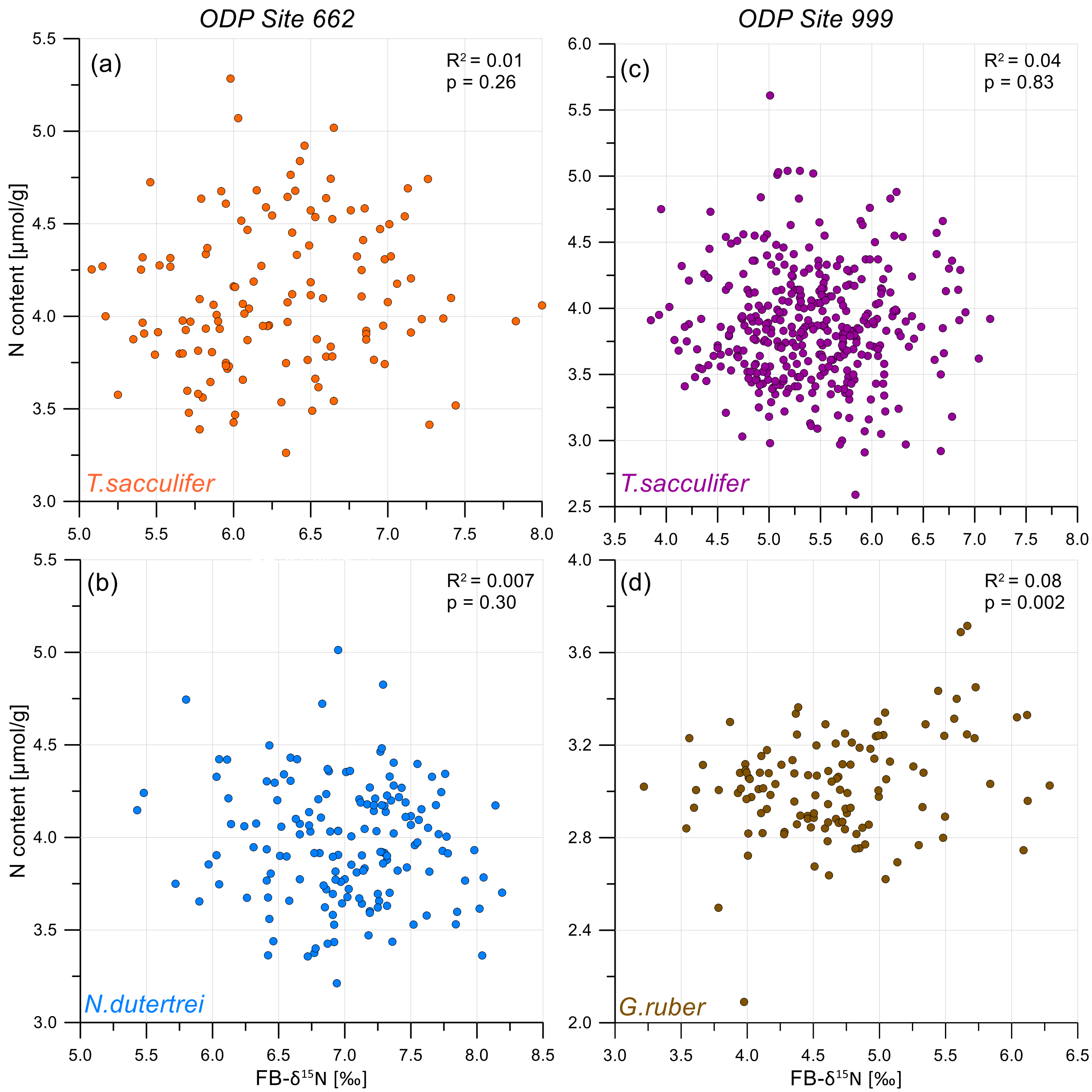

Fig. S4. Cross-plots of N content versus FB-$\delta^{15}N$ data from all species measured in the current study. (a) *T.sacculifer* data measured at ODP Site 662**.** (b) *N.dutertrei* data measured at ODP Site 662. (c) *T.sacculifer* data measured at ODP Site 999. (d) *G.ruber* data measured at ODP Site 999. For all species we find no relationship between N content and FB-$\delta^{15}N$. Linear regression results are shown for each panel; for *G. ruber*, a weak but significant positive correlation is observed ($R^2 = 0.08$, $p = 0.002$), whereas other relationships are not significant.

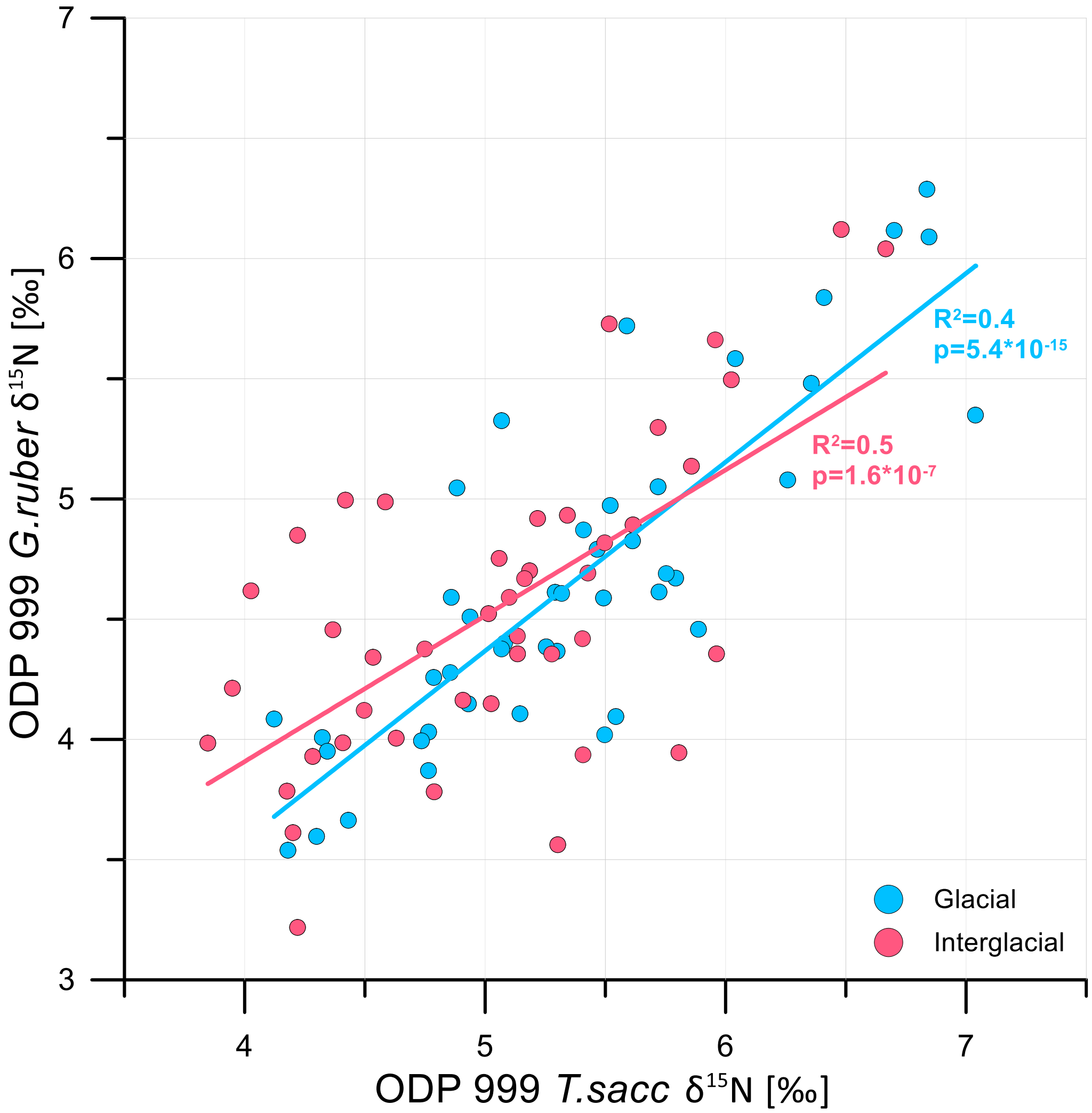

Fig. S5. Comparison of FB-δ^{15}N data from the two species measured at ODP Site 999. The data is divided into glacial (blue) and interglacial (peach) based on the LR04 δ^{18}O MIS boundaries (2). Solid lines indicate the linear regressions for each climate state. Both glacial and interglacial data subsets show strong correlations between the species ($R^2 = 0.7$, $p = 5.4\times10^{-15}$ for glacials; $R^2 = 0.5$, $p = 1.6\times10^{-7}$ for interglacials; regression analysis described in Materials and Methods, and results are presented in Table S4). These interspecies relationships illustrate that while there is a small systematic offset between species, both show consistent δ^{15}N trends across climate states. Therefore, using one or the other in this case would not change our interpretation, justifying the use of *T.sacc* as representative of N cycle changes.

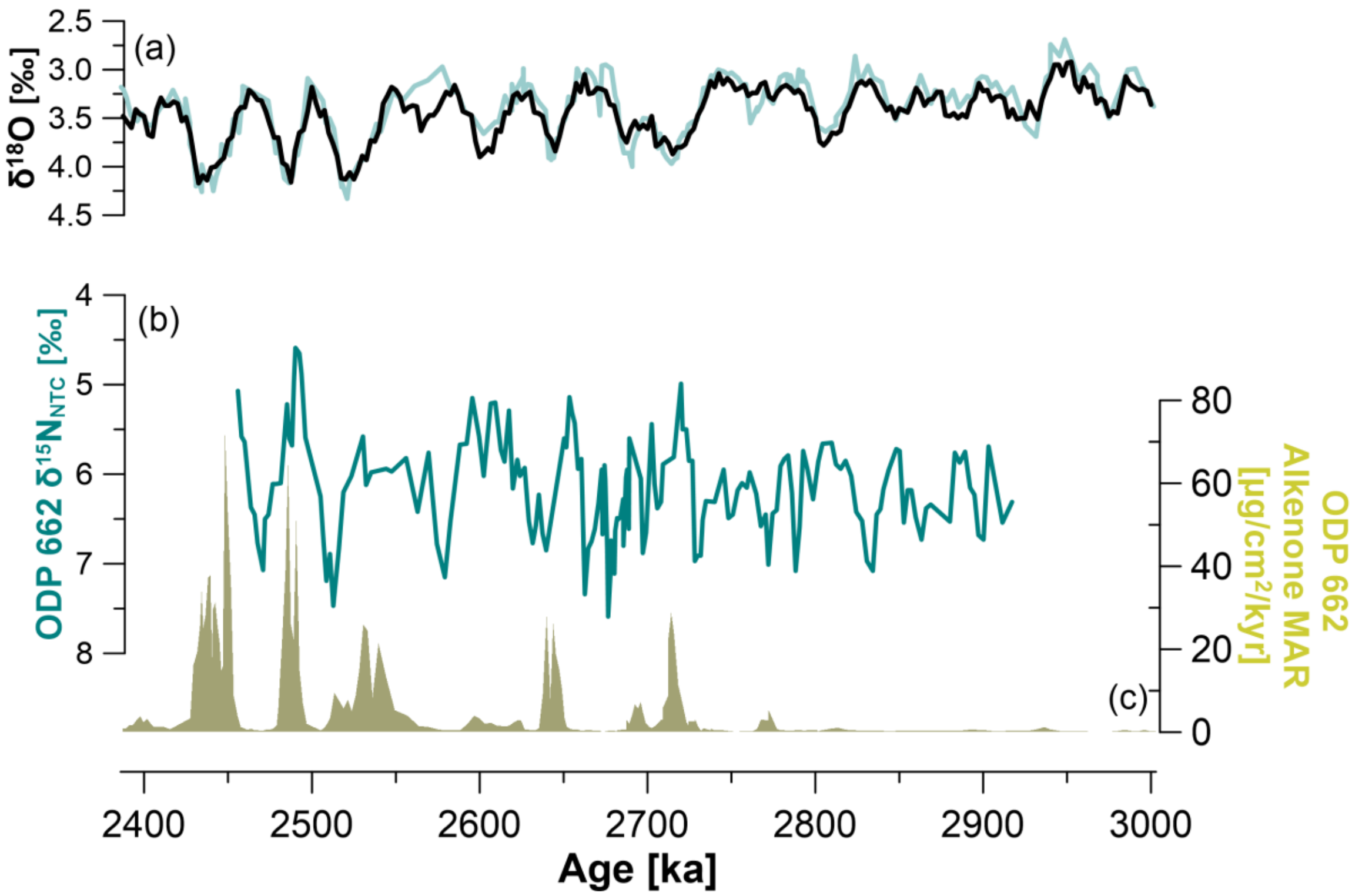

Fig. S6. Orbitally tuned age model for Site 662, FB-δ^{15}N and alkenone Mass Accumulation Rate (MAR) data. (a) LR04 δ^{18}O data (black) plotted with ODP 662 (light blue) benthic δ^{18}O record (2), on which the age model development was based. (b) FB-δ^{15}N ODP 662 data (teal; this study; ref. 3). (c) ODP 662 alkenone MAR (khaki; ref. 3).

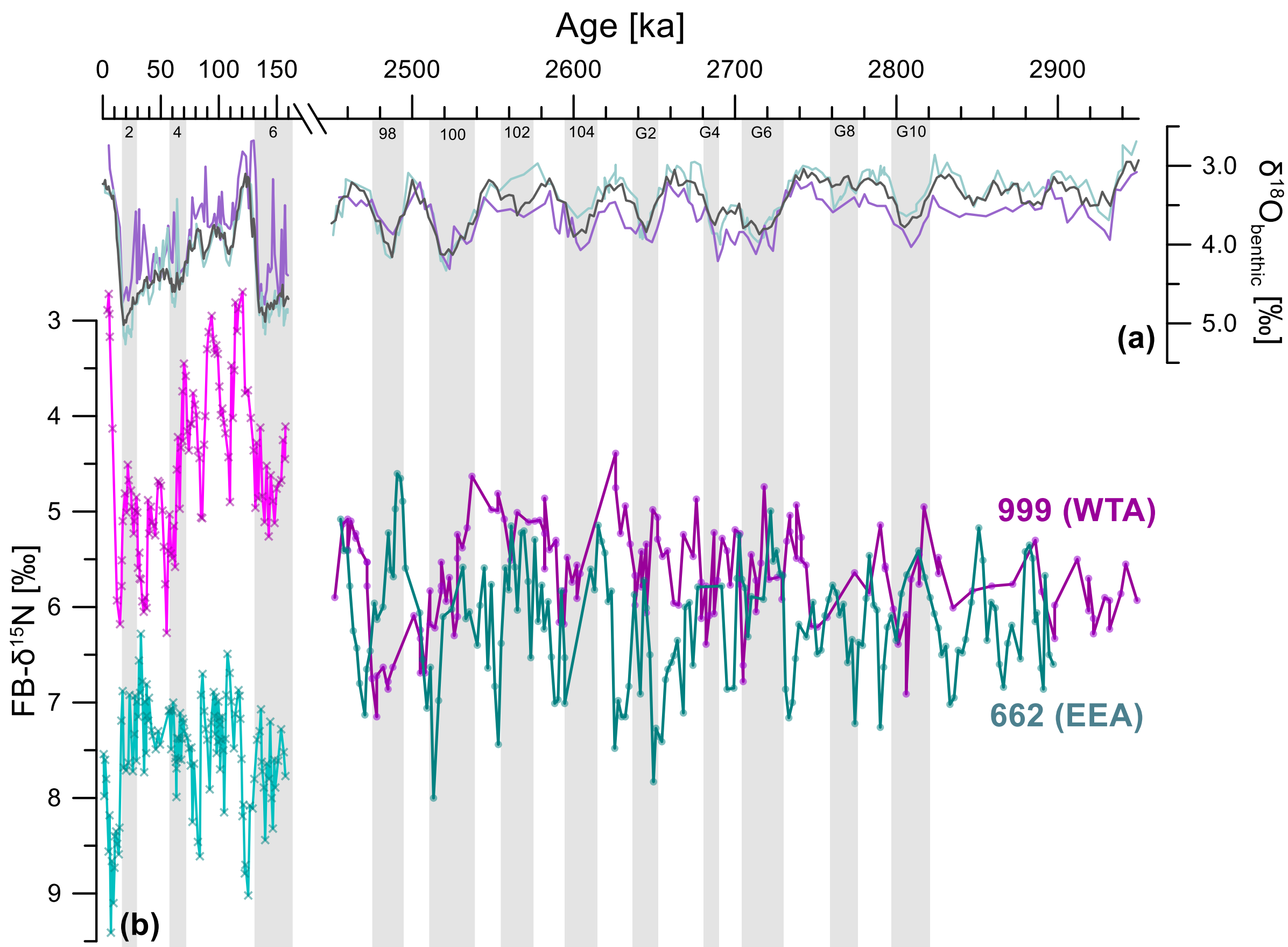

Fig. S7. Zoom-in on FB-δ^{15}N data from ODP Sites 999 in the Western Tropical Atlantic and 662 in the Eastern Equatorial Atlantic, compared to data from the last 160 kyr (4). (a) δ^{18}O data for Site 999 (light purple) and 662 (light blue) compared to the LR04 benthic δ^{18}O stack (grey; 2). (b) FB-δ^{15}N data from Sites 999 (this study in purple circles and ref. 5 in pink axes) and 662 (this study in teal circles and ref. 4 in light teal axes). The vertical grey bands represent Marine Isotope Stages (MIS) since ~2.8 Ma. MIS boundaries were assigned using LR04 information (2).

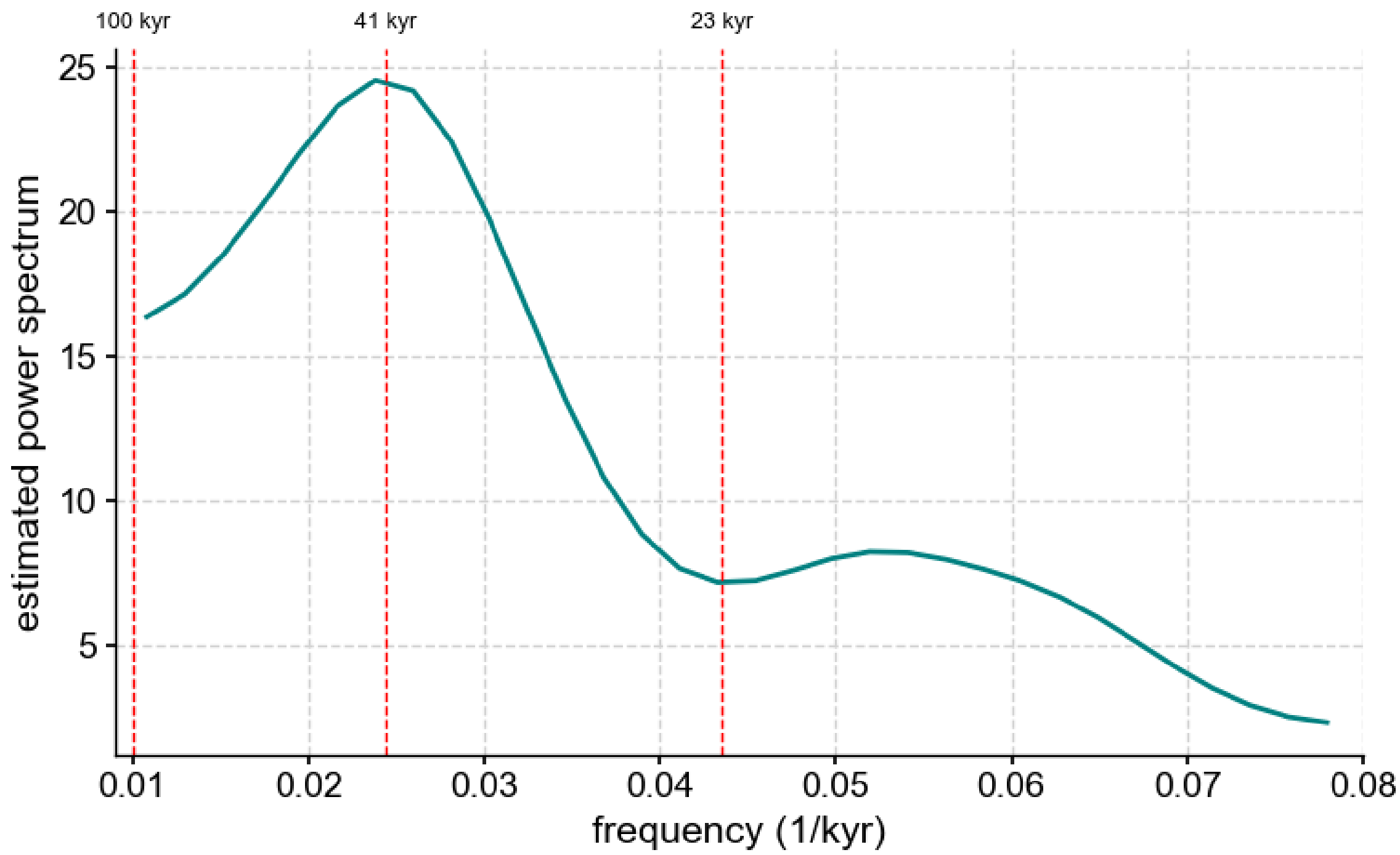

Fig. S8. Power spectrum of FB-δ^{15}N PPT values from ODP Site 662 calculated using the multi-taper method (MTM) in Pyleoclim (6) after resampling over a 3 kyr interval, matching the average sampling resolution at this site. The teal curve shows spectral power as a function of frequency (cycles per kyr). Vertical red dashed lines mark the orbital-scale periodicities of 100 kyr, 41 kyr, and 23 kyr. The analysis highlights the dominant variance structure in the δ^{15}N record used to evaluate orbital-scale nitrogen-cycle variability.

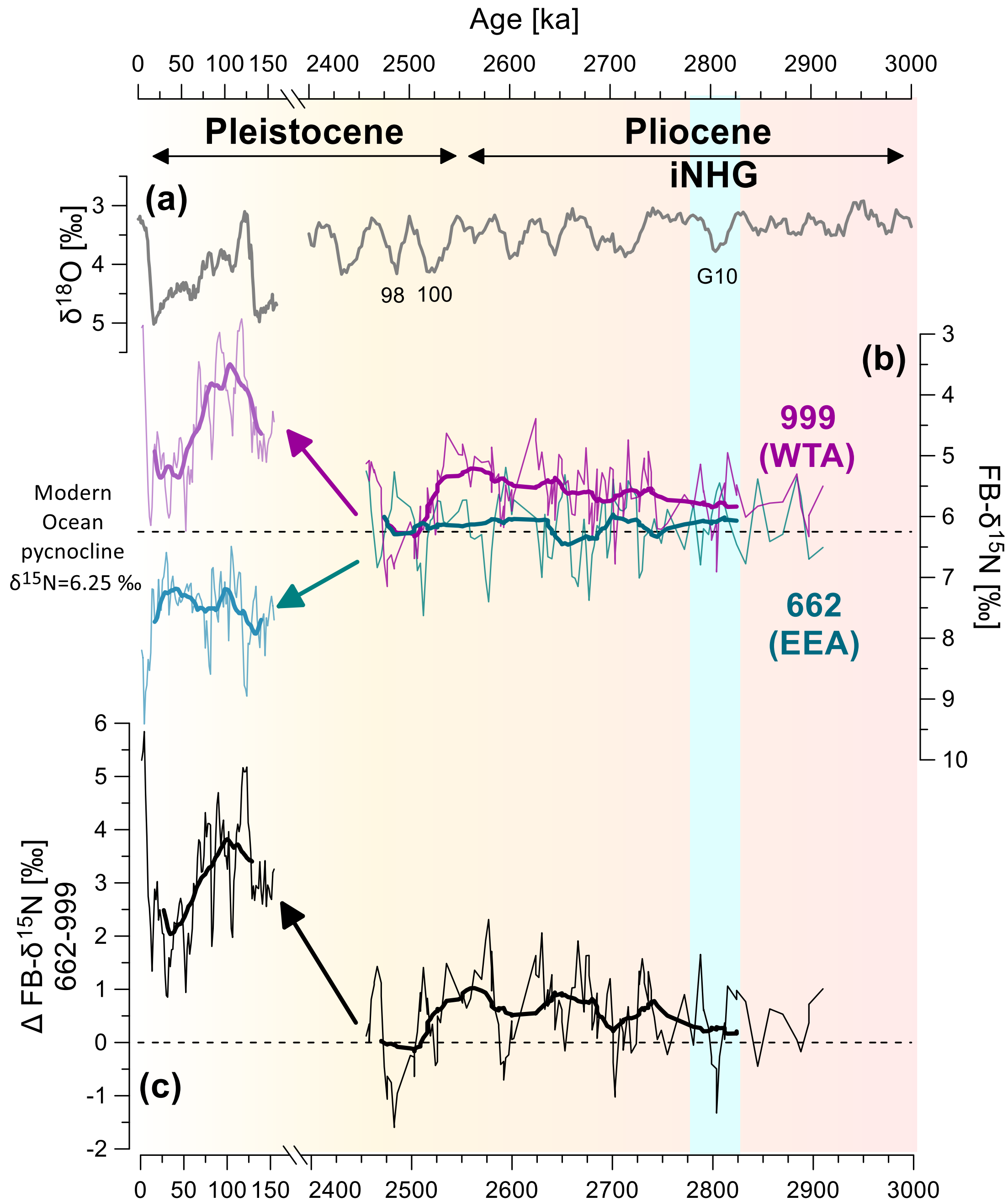

Fig. S9. δ^{15}N data from ODP Sites 999 in the Western Tropical Atlantic and 662 in the Eastern Equatorial Atlantic, and the difference between them, compared to data from the last 160 kyr (4). (a) LR04 benthic δ^{18}O stack (grey; 2). (b) δ^{15}N data from Sites 999 (this study in purple and ref. 5 in lilac) and 662 (this study in teal and ref. 4 in blue). (c) The difference (Δ FB-δ^{15}N= FB-δ$^{15}N_{662}$ - FB-δ$^{15}N_{999}$) between δ^{15}N of the two sites. Solid thicker lines represent smoothing over 21-point window for all records to emphasize the long-term trends, showing a smaller difference between the EEA and WTA over the PPT versus the past 160 kyr. The black thick arrow indicates the increase in Δ FB-δ^{15}N through time. Δ FB-δ^{15}N was calculated using FB-δ^{15}N data from Site 662 that was interpolated to match sampling points from Site 999.

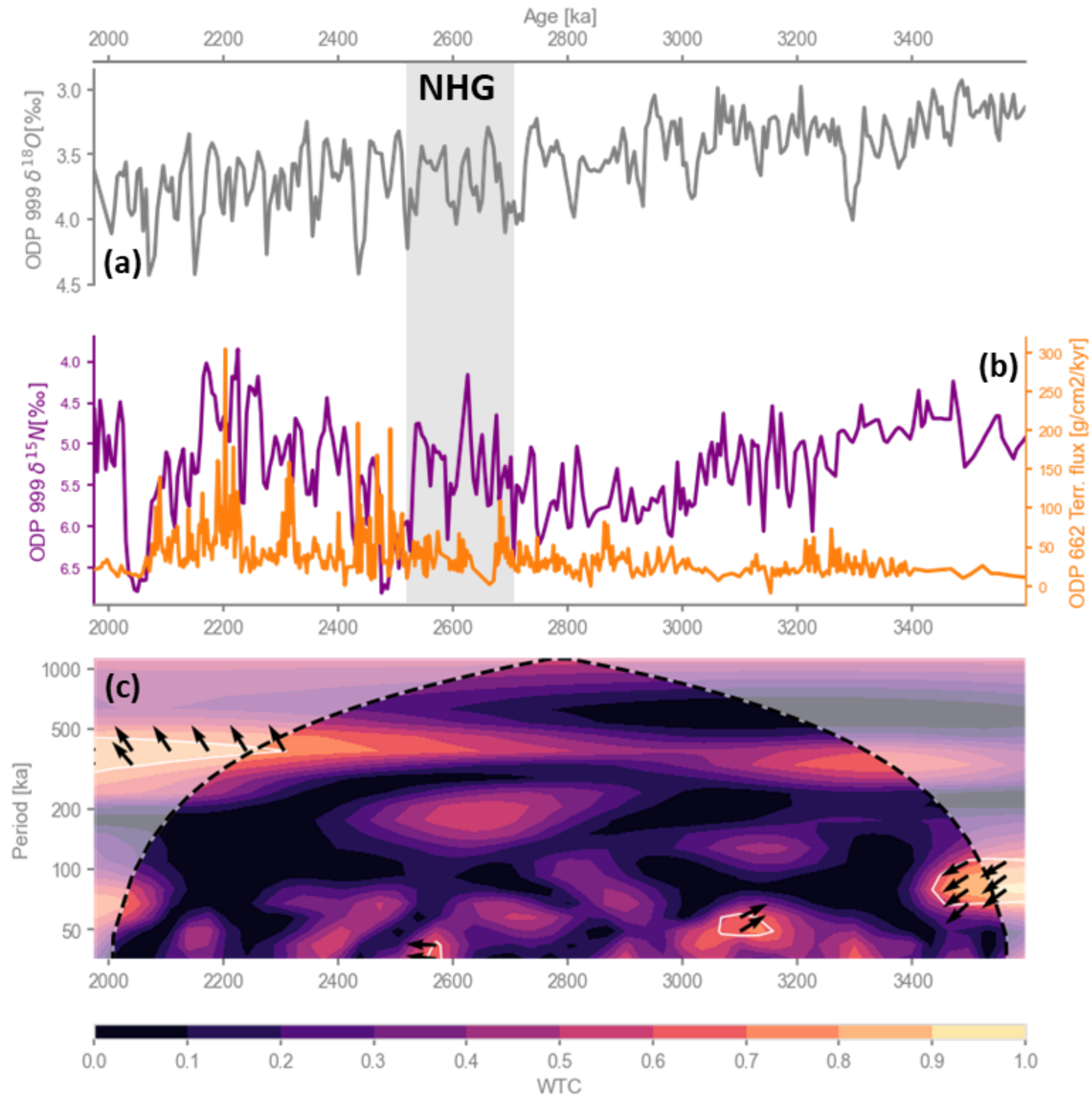

Fig. S10. Wavelet cross coherence (WTC) analysis of PPT δ^{15}N (this study) and a dust record from Site 662 (7)**.** (a) δ^{18}O record from ODP Site 999 (gray; 2). (b) ODP Site 999 FB-δ^{15}N record (purple; this study) and ODP Site 662 dust (terrigenous flux [g/cm^2/kyr]; orange; 7). (c) Wavelet cross coherence analysis results where a value of 1 is perfect coherence. We find no coherent relationship between ODP Site 999 FB-δ^{15}N and ODP Site 662 terrigenous flux. The white lines delineate regions of the scalogram that are significant against an AR(1) benchmark (AR(1) is a time series model where each value depends linearly on its immediately preceding value plus random noise, capturing short-term memory or persistence in the data), thus encircling “islands” of notable power. Plotting and analysis where conducted using Pyleoclim software (6).

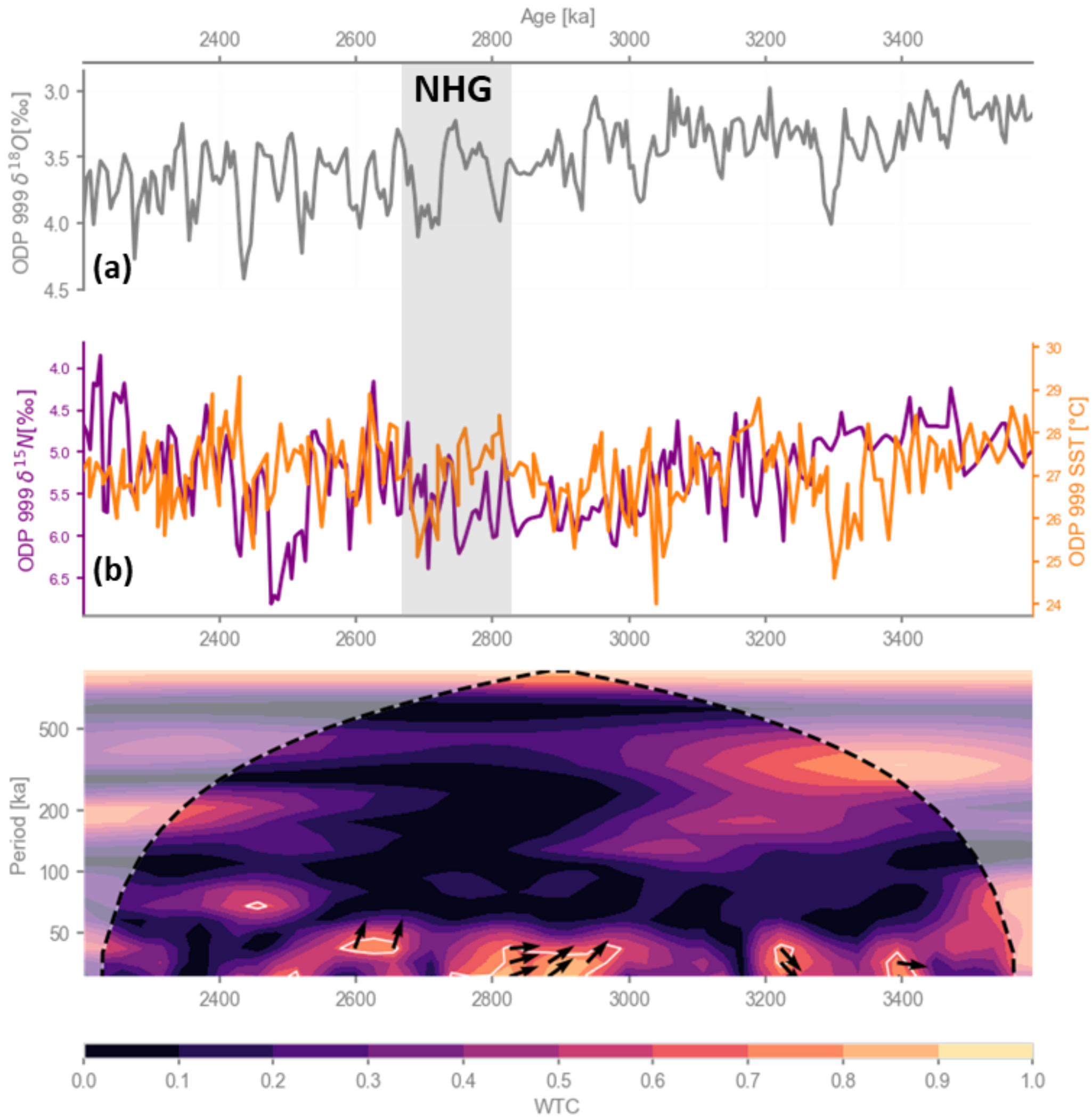

Fig. S11. Wavelet cross coherence (WTC) analysis of PPT δ^{15}N (this study) and SST records from Site 999 (8)**.** (a) δ^{18}O record from ODP Site 999 (gray; 2). (b) FB-δ^{15}N record (purple; this study) and SST (orange; 8) from Site 999. (c) Wavelet cross coherence analysis results where a value of 1 is perfect coherence. We find no coherent relationship between FB-δ^{15}N and SST at ODP Site 999. The white lines delineate regions of the scalogram that are significant against an AR(1) benchmark (AR(1) is a time series model where each value depends linearly on its immediately preceding value plus random noise, capturing short-term memory or persistence in the data), thus encircling “islands” of notable power. Plotting and analysis where conducted using Pyleoclim software (6).

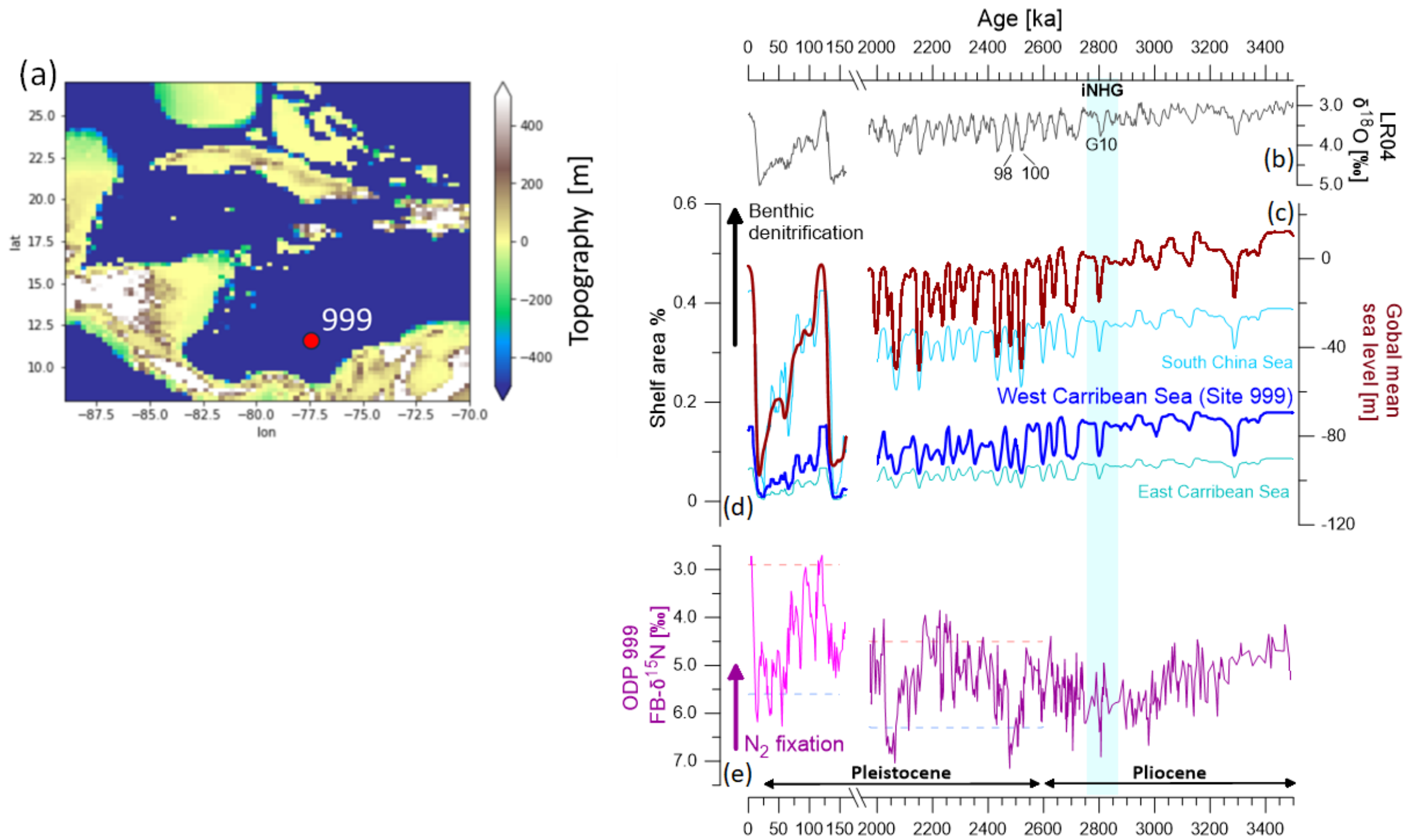

Fig. S12. Comparison of the FB-δ^{15}N data from Site 999 with shelf area percentage changes as a result of sea level fluctuations. (a) Map of the location of ODP Site 999 and the surrounding shelf area (defined as the water depth between 0 and 150 m, see *Materials and Methods* for more details) (b) LR04 benthic δ^{18}O stack (2). (c) the global mean sea level curve is plotted in dark brown (9) and is compared to (d) changes in shelf area percentage (%) in different basins, where the Western Caribbean Sea area is plotted in dark blue, Eastern Caribbean is plotted in turquois and the South China Sea is plotted in light blue. (e) FB-δ^{15}N from ODP Site 999. The dashed horizontal lines mark the interglacial (orange) and glacial (blue) average FB-δ^{15}N values for the last 160 kyr and the Early Pleistocene respectively. The changes in shelf area percentage are larger during the last 160 kyr in comparison to the PPT. During the PPT, the changes in shelf area percentage are largest in the South China Sea. Shelf area percentage changes are larger in the West Caribbean basin than in the Eastern Caribbean, which could have an effect on the amount of benthic denitrification as suggested in previous studies (10). Each drop in shelf area percentage corresponds to an increase in FB-δ^{15}N, which is interpreted to represent a reduction in N_2 fixation.

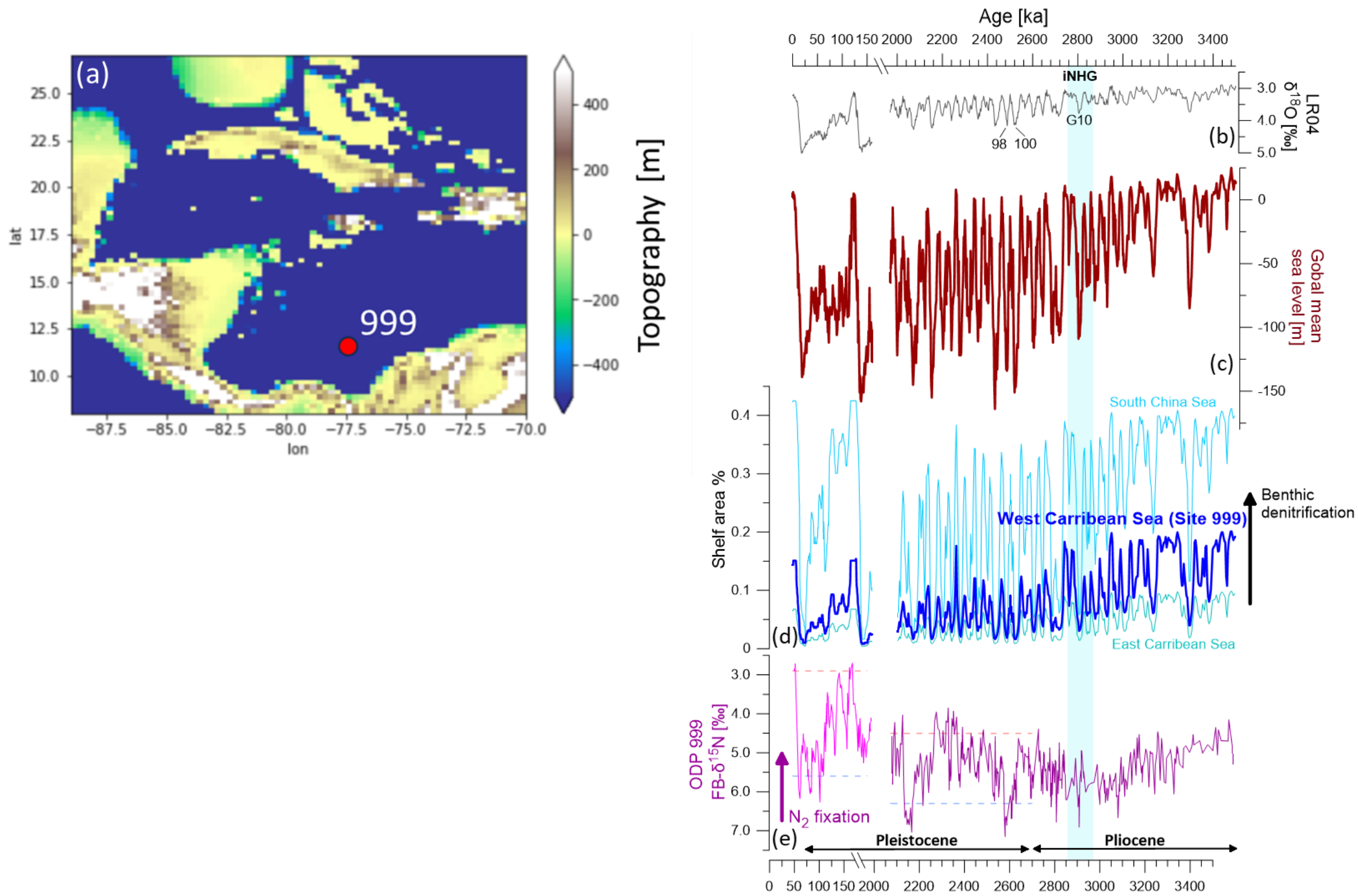

Fig. S13. Comparison of modeled West Caribbean shelf-area percentage as in Fig. S12, using a more recent Plio-Pleistocene global mean sea-level reconstruction (11). (a) Map of the location of ODP Site 999 and the surrounding shelf area (defined as the water depth between 0 and 150 m; see Materials and Methods for more details). (b) LR04 benthic $\delta^{18}O$ stack (2). (c) The global mean sea-level curve (11). (d) Changes in shelf area percentage (%) in different basins, where the Western Caribbean Sea area is plotted in dark blue, Eastern Caribbean in turquoise, and the South China Sea in light blue. (e) FB-$\delta^{15}N$ from ODP Site 999. The dashed horizontal lines mark the interglacial (orange) and glacial (blue) average FB-$\delta^{15}N$ values for the last 160 kyr and the Early Pleistocene, respectively. While this reconstruction produces lower absolute sea-level estimates after the iNHG, it shows a similar increase in variability after ~2.8 Ma. Both reconstructions are therefore consistent with the timing and direction of glacial FB-$\delta^{15}N$ changes at Site 999.

Supplementary Data 1 (separate file). ODP Site 999 FB-$\delta^{15}N$.

Supplementary Data 2 (separate file). ODP Site 662 FB-$\delta^{15}N$.

Supplementary Data 3 (separate file). ODP Site 662 age model LR04 tie points.

Supplementary Data 4 (separate file). Summary of $\delta^{15}N$ comparison between *T.sacculifer* and *G.ruber* for Site 999.

SI References

1. P. A. Rafter, A. Bagnell, D. Marconi, T. DeVries, Global trends in marine nitrate N isotopes from observations and a neural network-based climatology. *Biogeosciences* **16**, 2617–2633 (2019).
2. L. E. Lisiecki, M. E. Raymo, A Pliocene-Pleistocene stack of 57 globally distributed benthic d18O records. *Paleoceanography* **20**, 1–17 (2005).
3. K. T. Lawrence, *et al.*, Time-transgressive North Atlantic productivity changes upon Northern Hemisphere glaciation. *Paleoceanography* **28**, 740–751 (2013).
4. A. Auderset, *et al.*, Sea Level Modulation of Atlantic Nitrogen Fixation Over Glacial Cycles. *Paleoceanogr. Paleoclimatology* **39**, 1–21 (2024).
5. M. Straub, *et al.*, Nutrient conditions in the subpolar North Atlantic during the last glacial period reconstructed from foraminifera-bound nitrogen isotopes. **28**, 79–90 (2013).
6. D. Khider, *et al.*, Pyleoclim : Paleoclimate Timeseries Analysis and Visualization With Python Paleoceanography and Paleoclimatology. *Paleoceanogr. Paleoclimatologyoge* (2022). https://doi.org/10.1029/2022PA004509.
7. W. F. Ruddiman, *et al.*, Late Miocene to Pleistocene evolution of climate in Africa and the low-latitude Atlantic: overview of Leg 108 results. *Proc., Sci. results, ODP, Leg 108, East. Trop. Atl.* **108**, 463–484 (1989).
8. C. L. O'Brien, *et al.*, High sea surface temperatures in tropical warm pools during the Pliocene. *Nat. Geosci.* **7**, 606–611 (2014).
9. C. J. Berends, B. De Boer, R. S. W. Van De Wal, Reconstructing the evolution of ice sheets , sea level , and atmospheric CO 2 during the past 3 . 6 million years. *Clim. Past* **17**, 361–377 (2021).
10. H. Ren, *et al.*, Impact of glacial/interglacial sea level change on the ocean nitrogen cycle. *Proc. Natl. Acad. Sci. U. S. A.* **114**, E6759–E6766 (2017).
11. P. U. Clark, *et al.*, Global mean sea level over the past 4 . 5 million years Global mean sea level over the past. **8389**, 0–10 (2025).
