# Supplementary material for: Reduced N2 fixation in the tropical Atlantic Ocean during the warm late Pliocene
Source: Nat Commun. 2026 Aug 5;17:7931. doi: 10.1038/s41467-026-75846-4 (PMC13448806; doi:10.1038/s41467-026-75846-4)
Supplement: Supplementary file 2 [file 41467_2026_75846_MOESM2_ESM.pdf]

Description of Additional Supplementary Files

File Name: Supplementary Data 1
Description: ODP Site 999 FB-δ ^{15}N.

File Name: Supplementary Data 2
Description: ODP Site 662 FB-δ ^{15}N

File Name: Supplementary Data 3
Description: ODP Site 662 age model LR04 tie points.

File Name: Supplementary Data 4
Description: Summary of $\delta^{15}N$ comparison between *T.sacculifer* and *G.ruber* for Site 999.
