# Supplementary material for: Reduced N2 fixation in the tropical Atlantic Ocean during the warm late Pliocene
Source: Nat Commun. 2026 Aug 5;17:7931. doi: 10.1038/s41467-026-75846-4 (PMC13448806; doi:10.1038/s41467-026-75846-4)
Supplement: Supplementary file 3 [file 41467_2026_75846_MOESM3_ESM.pdf]

Sample lable (if available)	Depth (mcd)	Age (ka)	δ15N *Tsacc* (‰ vs. air)	sd (replicates)	*Tsacc* N content [µmol/mg]	δ15N *Gruber* (‰ vs. air)	sd (replicates)	*Gruber* N content (µmol/g)
999-8-1-5-7	64.65	1976	4.58		3.21			
999-8-1-15-17	64.74	1979	5.81		3.83	4.99		3.30
999-8-1-35-37	64.94	1984	4.63		3.53			
999-8-1-55-57	65.13	1989	4.22		3.88			
999-8-1-75-77	65.32	1994	5.59		4.09	3.95	0.17	3.08
999-8-1-95-97	65.51	1999	4.88		3.46			
999-8-1-115-117	65.70	2004	4.94		3.43			
999-8-1-135-137	65.89	2009	5.61		3.43			
999-8-2-5-7	66.08	2014	4.76		3.58	4.01		2.72
999-8-2-25-27	66.27	2019	4.79		3.52			
999-8-2-45-47	66.47	2024	4.03		4.01			
999-8-2-65-67	66.66	2029	5.86	0.59	3.58			
999-8-2-85-87	66.85	2034	6.67		3.50	4.85		2.75
999-8-2-105-107	67.04	2039	6.48	0.13	3.86			
999-8-2-125-127	67.23	2044	6.84		4.14			
999-8-2-145-147	67.42	2048	6.70		3.66	5.72		3.23
999-8-3-15-17	67.61	2053	6.85		3.91			
999-8-3-35-37	67.81	2059	6.41		3.77			
999-8-3-55-57	68.00	2064	7.04		3.62	5.05		2.62
999-8-3-75-77	68.19	2068	6.26		3.82			
999-8-3-94-96	68.37	2073	6.04	0.48	4.03	4.51		2.68
999-8-3-115-117	68.57	2078	5.47		4.04			
999-8-3-135-137	68.76	2083	5.79		3.83			
999-8-4-7-9	68.97	2089	5.30		3.78	4.83		2.82
999-8-4-25-27	69.14	2095	5.62		3.61			
999-8-4-45-47	69.34	2102	5.01		3.56	3.87		3.30
999-8-4-65-67	69.53	2108	5.30	0.30	4.08			
999-8-4-85-87	69.72	2114	6.36		3.71			
999-8-4-105-107	69.91	2119	5.49		3.65	3.78		2.50
999-8-4-125-127	70.10	2124	5.25	0.26	4.14			
999-8-4-145-147	70.29	2131	5.06		3.92			
999-8-5-5-7	70.39	2135	5.52		3.98	4.62		2.64
999-8-5-15-17	70.48	2139	6.02	0.15	3.88			
999-8-5-35-37	70.68	2147	4.99		3.46			
999-8-5-55-57	70.87	2153	5.54	0.21	4.55			
999-8-5-75-77	71.06	2160	5.07	0.04	4.48			
999-8-5-95-97	71.25	2166	4.18		3.41	5.14		2.69
999-8-5-115-117	71.44	2173	3.95		4.75			
999-8-5-135-137	71.63	2180	4.37	0.11	4.26			
999-8-6-5-7	71.82	2186	4.42	0.24	4.45			
999-8-6-25-27	72.01	2193	4.86	0.21	4.36	6.04	0.21	3.32
999-8-6-45-47	72.21	2200	4.79		4.14	6.12		2.96
999-8-6-55-57	72.30	2203	4.43		4.73	6.29		3.03
999-8-6-65-67	72.40	2206	4.76		3.87	6.12		3.33
999-8-6-75-77	72.49	2209	5.02		4.33	6.09		2.75
999-8-6-85-87	72.59	2213	4.91		3.64	5.84		3.03
999-8-6-95-97	72.68	2216	4.18		3.86	5.35		3.29
999-8-6-105-107	72.78	2219	4.20		3.75	5.08		3.13
999-8-6-115-117	72.88	2223	4.22		4.21	5.58	0.06	3.40
999-8-6-125-127	72.97	2226	3.85		3.91	4.79		3.21
999-8-6-135-137	73.07	2230	5.96		3.96	4.67		2.88
999-8-6-145-147	73.16	2233	5.17		3.56	3.56		3.23
999-8-7-5-7	73.26	2236	5.72		3.81	4.89		2.77

999-8-7-15-17	73.35	2239	5.52		3.87	4.52		
999-8-7-15-17	73.35	2239	4.74	0.55	3.92	4.52		
999-8-7-25-27	73.45	2243	4.12		3.68	4.37		3.34
999-8-7-35-37	73.55	2246	4.30		3.69	5.48		2.80
999-8-7-45-47	73.64	2249	4.32		3.55	4.59		2.84
999-8-7-45-47	73.64	2249	3.93	0.28	3.95	4.59		2.84
999-8-7-55-57	73.74	2253	4.53		3.86	4.39		3.36
999-8-7-65-67	73.83	2256	4.41		3.57	4.75		2.91
999-8-0-5-7	73.93	2259	4.28		3.48	5.73		3.45
----	74.02	2262				5.50		2.89
999-8-0-15-17	74.02	2263	4.08		3.76			
999-9-1-15-17	74.24	2270	5.34		3.80	5.66		3.72
999-9-1-25-27	74.34	2273	5.72		3.74	5.33		2.93
999-9-1-35-37	74.43	2276	5.54		3.86	3.54		2.84
999-9-1-45-47	74.53	2278	5.89		4.66	4.21		3.03
999-9-1-55-57	74.62	2281	5.29		4.01	4.46		2.89
999-9-1-65-67	74.72	2284	4.73		3.93	4.99		2.98
999-9-1-75-77	74.81	2286	5.15		4.33	4.59		3.29
999-9-1-85-87	74.91	2289	4.34		3.92	4.26		3.12
999-9-1-95-97	75.00	2292	5.19		3.39	3.66	0.08	3.11
999-9-1-105-107	75.10	2296	5.02		3.84	4.03	0.03	2.98
999-9-1-115-117	75.19	2299	5.31		4.10	4.15		3.18
999-9-1-125-127	75.29	2302	5.22		3.86	4.16		3.08
----	75.38	2305				3.78	0.01	3.01
999-9-1-135-137	75.38	2306	5.14		3.97			
999-9-1-145-147	75.48	2309	5.14		3.83	3.61	0.04	3.01
999-9-2-5-7	75.57	2312	4.88		3.82	3.22		3.02
999-9-2-15-17	75.67	2314	4.93		3.99	3.99	0.11	3.12
999-9-2-25-27	75.76	2317	4.87		3.94	4.36		3.08
999-9-2-35-37	75.86	2320	5.41		4.01	5.30		2.77
999-9-2-45-47	75.95	2322	5.52		3.72	5.05	0.14	3.05
999-9-2-55-57	76.05	2325	4.87		3.76			
999-9-2-65-67	76.14	2327	4.50		3.72	4.97	0.22	3.24
999-9-2-75-77	76.24	2330	4.85		3.77	4.09		3.01
999-9-2-85-87	76.33	2333	4.58		4.14	3.60	0.11	2.93
999-9-2-115-117	76.62	2343	5.43		5.02	4.01	0.08	2.82
999-9-2-125-127	76.71	2346	5.61		4.09	4.34	0.14	3.14
999-9-2-135-137	76.81	2349	5.50		4.04	3.99	0.08	3.09
999-9-2-145-147	76.90	2352	5.66		4.14	3.93	0.02	2.99
999-9-3-5-7	77.00	2356	5.32		3.72	4.01	0.03	3.06
999-9-3-15-17	77.09	2358	5.22		4.11	4.93	0.36	3.18
999-9-3-25-27	77.18	2361	5.75		3.98	4.61	0.10	3.09
999-9-3-35-37	77.28	2363	5.12		4.14	4.10	0.26	3.08
999-9-3-45-47	77.37	2366	5.08		3.75	4.46	0.05	2.88
999-9-3-55-57	77.47	2369	4.61		3.79	4.61	0.15	2.87
999-9-3-65-67	77.56	2372	5.07		4.28	3.99	0.14	2.97
999-9-3-75-77	77.66	2376	4.84		3.90	4.11	0.28	3.15
999-9-3-85-87	77.75	2379	4.50		3.70	3.95	0.12	3.01
999-9-3-95-97	77.85	2382	4.41		4.23	4.70		3.01
999-9-3-105-107	77.94	2385	4.75		3.68	4.74	0.29	2.84
999-9-3-115-117	78.04	2389	4.84		4.36	4.50	0.17	2.89
----	78.23	2395				4.92	0.02	2.86
----	78.30	2398				4.72	0.03	3.12
----	78.44	2403				4.36		2.96
999-9-4-15-17	78.51	2404	5.50		3.49	4.28	0.05	2.82
999-9-4-25-27	78.61	2407	4.82		3.93	4.15		2.92

999-9-4-35-37	78.70	2410	4.75		4.26	4.61	0.44	2.94
999-9-4-55-57	78.89	2415	4.98		4.40	4.87	0.08	2.84
999-9-4-65-67	78.99	2418	5.16		3.74	4.74	0.01	2.93
999-9-4-75-77	79.08	2420	5.21		4.63	4.12	0.12	2.82
999-9-4-85-87	79.18	2423	5.96		3.64	4.28	0.04	2.81
999-9-4-95-97	79.27	2426	6.11		4.22	4.52		3.20
999-9-4-105-107	79.37	2428	6.03		4.50	4.69	0.03	2.86
999-9-4-115-117	79.46	2431	6.24		4.88	4.76		2.99
999-9-4-125-127	79.56	2434	4.58		4.54	4.82	0.17	2.75
999-9-4-135-137	79.65	2436	5.55		4.23	4.72	0.02	2.87
999-9-4-145-147	79.75	2439	5.85		3.77	4.61	0.11	2.78
999-9-5-5-7	79.84	2441	5.39		4.30	4.99	0.08	3.00
999-9-5-15-17	79.94	2444	5.53		3.87	4.69	0.06	3.06
999-9-5-25-27	80.03	2447	5.49		4.13	4.50	0.06	2.91
999-9-5-35-37	80.13	2449	6.14		3.83	4.40	0.12	2.90
----	80.22	2452	5.90		4.22	4.78		2.93
----	80.32	2455				4.38	0.05	3.25
----	80.41	2457	5.13		4.06	4.48		2.84
999-9-5-75-77	80.51	2460	5.08		3.99	4.12	0.04	3.01
----	80.51	2460	5.41	0.23	3.87	4.12	0.04	3.01
----	80.60	2462	5.10		3.97	4.53	0.17	3.07
----	80.70	2465	5.28		4.14	4.38	0.07	2.86
999-9-5-95-97	80.70	2465	5.23	0.03	3.64	4.38	0.07	2.86
----	80.79	2468	5.41		4.06	4.52	0.10	2.98
999-9-5-115-117	80.89	2472	5.53		4.15	4.68	0.04	3.06
999-9-5-115-117	80.89	2472	5.53		4.19	4.68	0.04	3.06
----	80.89	2472	5.78	0.15	4.28	4.68	0.04	3.06
----	80.98	2475	6.75		4.30	4.10	0.33	2.91
----	81.08	2478	7.15		3.92	4.00		3.08
999-9-5-135-137	81.08	2478	6.72	0.30	4.13	4.00		3.08
----	81.17	2482	6.63		4.57	4.02		3.05
999-9-6-5-7	81.27	2485	6.86		4.29	4.78		3.12
----	81.27	2485	6.78		4.36	4.78		3.12
----	81.36	2488	6.63		4.41	3.98		2.09
----	81.46	2492				4.18	0.10	2.98
----	81.55	2495				4.67	0.17	3.21
----	81.65	2498				5.33	0.16	3.08
----	81.74	2501	6.09		4.15	5.66	0.40	3.25
999-9-6-65-67	81.84	2505	6.33		3.90	5.57	0.14	3.31
999-9-6-65-67	81.84	2505	6.69		3.85	5.57	0.14	3.31
----	81.84	2505	6.24		3.98	5.57	0.14	3.31
----	81.93	2508	6.69		4.66	5.62	0.10	3.69
999-9-6-85-87	82.03	2511	5.83		3.80	5.44	0.12	3.43
----	82.03	2511	6.18		4.83	5.44	0.12	3.43
----	82.12	2514	6.22		4.55	5.04	0.18	3.34
999-9-6-105-107	82.21	2518	5.83		4.30	5.03	0.17	3.24
999-9-6-105-107	82.21	2518	5.78		4.18	5.03	0.17	3.24
----	82.21	2518	5.53		4.09	5.03	0.17	3.24
----	82.31	2521	5.94		4.37	4.64		3.04
999-9-6-125-127	82.40	2523	5.69		4.37	5.26	0.19	3.11
----	82.50	2526	6.30		4.54	4.99	0.11	3.24
999-9-6-145-147	82.59	2528	6.10		4.14	4.96	0.22	3.14
----	82.59	2528	5.49		4.26	4.96	0.22	3.14
999-9-6-145-147	82.59	2528	5.24	0.44	4.30	4.96	0.22	3.14
999-9-7-5-7	82.69	2531	5.38		3.91	4.85	0.08	3.19
999-9-7-15-17	82.78	2534	5.17		3.36			

999-9-7-25-27	82.88	2537	4.63		4.49		
999-9-7-55-57	83.16	2549	4.98		3.51		
999-9-7-65-67	83.26	2553	4.99		3.89		
999-9-7-65-67	83.26	2553	4.81		4.11		
999-9-0-5-7	83.35	2557	5.08		5.01		
999-9-0-15-17	83.45	2561	5.51		4.15		
999-9-0-25-27	83.54	2565	5.01		5.61		
999-10-1-15-17	83.74	2572	5.11		3.91		
999-10-1-25-27	83.84	2576	5.10		4.39		
999-10-1-35-37	83.93	2579	5.09		3.71		
999-10-1-45-47	84.03	2582	5.23		4.24	4.46	3.07
999-10-1-45-47	84.03	2582	5.60	0.86	3.61	4.46	3.07
999-10-1-45-47	84.03	2582	4.86	0.37	4.12	4.46	3.07
999-10-1-55-57	84.12	2585	5.40		3.13		
999-10-1-65-67	84.22	2589	5.30		4.14	4.74	3.25
999-10-1-65-67	84.22	2589	5.34		4.27	4.74	3.25
999-10-1-75-77	84.31	2591	6.16		4.30		
999-10-1-85-87	84.40	2594	5.83		4.17	5.49	3.24
999-10-1-85-87	84.40	2594	6.18		4.12	5.49	3.24
999-10-1-95-97	84.50	2596	5.48		4.36		
999-10-1-105-107	84.59	2599	5.74		3.81		
999-10-1-115-117	84.69	2602	5.56		3.91		
999-10-1-115-117	84.69	2602	5.91	0.24	4.63		
999-10-1-125-127	84.78	2604	5.65		3.93		
999-10-2-45-47	85.45	2626	4.39		3.45		
999-10-2-45-47	85.45	2626	4.75	0.26	4.56		
999-10-2-55-56	85.54	2629	5.23		3.81		
999-10-2-65-67	85.63	2632	4.94		3.52		
999-10-2-75-77	85.73	2635	5.34		4.00		
999-10-2-85-87	85.82	2638	5.67		3.64		
999-10-2-85-87	85.82	2638	5.98	0.26	4.76		
----	85.92	2642	5.42		3.36		
999-10-2-95-97	85.92	2642	5.79	0.26	3.79		
999-10-2-105-107	86.01	2645	5.34		3.51		
999-10-2-105-107	86.01	2645	6.06	0.51	4.37		
999-10-2-115-117	86.11	2649	4.98		4.56		
999-10-2-125-127	86.20	2652	5.06		3.35		
999-10-2-125-127	86.20	2652	5.29	0.16	4.83		
999-10-2-135-137	86.30	2655	5.47		3.87		
999-10-2-145-147	86.39	2658	5.41		3.11		
999-10-3-5-7	86.49	2662	5.96		3.88		
----	86.58	2665	5.98		4.37		
999-10-3-25-27	86.68	2668	5.24		4.41		
999-10-3-45-47	86.86	2674	5.47		3.09		
999-10-3-55-57	86.96	2676	4.87		3.58		
999-10-3-65-67	87.05	2679	5.74		3.36		
999-10-3-65-67	87.05	2679	6.12		3.50		
999-10-3-75-77	87.15	2682	5.78		3.31		
999-10-3-75-77	87.15	2682	6.39	0.44	4.24		
999-10-3-85-87	87.24	2684	6.09		4.11		
999-10-3-95-97	87.34	2687	5.22		3.51		
999-10-3-95-97	87.34	2687	6.07	0.60	4.04		
999-10-3-105-107	87.43	2689	5.72		3.77		
999-10-3-115-117	87.53	2692	5.28		3.48		
999-10-3-125-127	87.62	2695	5.41		4.01		
999-10-3-135-137	87.72	2697	5.77		3.48		

999-10-3-145-147	87.81	2700	5.19		4.05
999-10-4-5-7	87.91	2703	5.23		3.74
999-10-4-5-7	87.91	2703	5.76	0.38	4.29
999-10-4-15-17	88.00	2705	6.78		3.18
999-10-4-15-17	88.00	2705	6.61	0.12	3.61
999-10-4-35-37	88.19	2710	5.45		3.41
999-10-4-45-47	88.28	2713	5.72		3.70
999-10-4-45-47	88.28	2713	6.05	0.23	3.92
999-10-4-55-57	88.38	2716	5.54		3.52
----	88.47	2718	4.74		3.94
999-10-4-75-77	88.57	2721	5.71		3.00
999-10-4-95-97	88.76	2726	5.69		2.97
999-10-4-105-107	88.85	2729	5.67		3.19
999-10-4-105-107	88.85	2729	5.92	0.18	3.91
999-10-4-115-117	88.95	2732	5.31		4.05
999-10-4-125-127	89.04	2734	5.04		3.40
999-10-4-125-127	89.04	2734	5.21	0.12	4.18
----	89.14	2738	5.49		4.65
999-10-4-135-137	89.14	2738	4.93	0.40	3.99
999-10-4-145-147	89.23	2741	5.27		3.58
999-10-4-145-147	89.23	2741	5.51	0.17	4.10
999-10-5-5-7	89.33	2744	5.56		4.33
999-10-5-15-17	89.42	2747	6.20		3.94
999-10-5-25-27	89.51	2751	6.21		3.98
999-10-5-45-47	89.70	2757	6.11		3.43
999-10-5-95-97	90.18	2774	5.64		3.54
999-10-5-125-127	90.46	2783	5.85		3.74
999-10-6-5-7	90.74	2790	5.14		3.61
999-10-6-15-17	90.84	2793	5.60		3.35
999-10-6-15-17	90.84	2793	5.57	0.03	3.83
999-10-6-35-37	91.03	2798	6.02		3.71
999-10-6-45-47	91.12	2801	6.26		3.24
999-10-6-45-47	91.12	2801	6.39	0.10	3.94
999-10-6-65-67	91.31	2806	6.08		3.72
999-10-6-65-67	91.31	2806	6.91	0.59	3.97
999-10-6-75-77	91.41	2809	5.71		3.86
999-10-6-95-97	91.60	2814	5.45		3.24
999-10-6-95-97	91.60	2814	5.76	0.22	3.53
999-10-6-105-107	91.69	2817	4.95	0.47	3.75
999-10-6-135-137	91.97	2826	5.65		3.79
999-10-6-135-137	91.97	2826	5.48	0.12	4.32
999-10-7-5-7	92.16	2835	6.01		3.90
999-10-7-35-37	92.45	2847	5.83		3.46
999-10-7-64-66	92.72	2859	5.78		3.36
999-10-0-25-27	93.02	2872	5.76		3.44
999-11-1-25-27	93.34	2886	5.30		3.48
999-11-1-35-37	93.43	2890	5.84		2.59
999-11-1-55-57	93.63	2898	6.33		2.97
999-11-1-55-57	93.63	2898	5.98	0.25	3.72
999-11-1-95-97	94.01	2912	5.50		3.74
999-11-1-115-117	94.20	2919	6.04		3.43
999-11-1-115-117	94.20	2919	5.70	0.25	4.06
999-11-1-125-127	94.29	2922	6.12	0.51	3.34
999-11-1-125-127	94.29	2922	6.28	0.11	3.79
999-11-1-145-147	94.48	2929	5.90		3.65
999-11-2-5-7	94.58	2932	5.93	0.51	3.07

999-11-2-5-7	94.58	2932	6.23	0.21	3.99
999-11-2-25-27	94.77	2939	5.86		3.85
999-11-2-35-37	94.87	2942	5.55	0.17	4.06
999-11-2-55-57	95.06	2949	5.93	0.51	2.91
999-11-2-65-67	95.15	2952	5.97	0.51	3.16
999-11-2-85-87	95.34	2959	5.69		3.63
999-11-2-95-97	95.44	2962	5.73	0.51	3.39
999-11-2-95-97	95.44	2962	5.72	0.01	4.02
999-11-2-115-117	95.63	2968	5.52		3.55
999-11-2-125-127	95.73	2971	6.13	0.51	3.22
999-11-2-145-147	95.92	2976	6.09		3.05
999-11-3-5-7	96.01	2978	6.67	0.51	2.92
999-11-3-25-27	96.20	2984	5.80		3.43
999-11-3-35-37	96.30	2986	5.99	0.51	3.31
999-11-3-35-37	96.30	2986	5.66	0.23	4.32
999-11-3-55-57	96.49	2992	5.11		3.42
999-11-3-65-67	96.59	2994	5.82		3.69
999-11-3-85-87	96.78	3000	5.73		3.65
999-11-3-95-97	96.87	3002	5.35		3.86
999-11-3-115-117	97.06	3008	6.12		3.58
999-11-3-125-127	97.16	3010	5.67		3.39
999-11-3-145-147	97.35	3016	5.76		3.68
999-11-4-5-7	97.44	3018	5.94		3.94
999-11-4-25-27	97.64	3024	5.51		3.65
999-11-4-35-37	97.73	3026	5.43		3.84
999-11-4-55-57	97.92	3031	5.12		3.71
999-11-4-65-67	98.02	3034	4.99		4.02
999-11-4-85-87	98.21	3039	5.82		3.50
999-11-4-95-97	98.30	3042	5.33		3.84
999-11-4-125-127	98.59	3050	4.90		3.25
999-11-4-145-147	98.78	3055	5.31		3.82
999-11-5-5-7	98.88	3058	5.58		3.56
999-11-5-25-27	99.07	3063	4.61		3.67
999-11-5-35-37	99.16	3066	5.14		3.45
999-11-5-55-57	99.35	3072	4.53		3.65
999-11-5-65-67	99.45	3075	5.12		3.70
999-11-5-85-87	99.64	3082	5.52		3.63
999-11-5-95-97	99.74	3085	5.33		3.66
999-11-5-95-97	99.74	3085	5.76	0.31	3.85
999-11-5-105-107	99.83	3088	5.38		3.60
999-11-5-125-127	100.02	3095	5.22		3.86
999-11-5-125-127	100.02	3095	5.22	0.00	4.25
999-11-5-145-147	100.21	3101	4.90		3.90
999-11-6-5-7	100.31	3105	4.97		3.88
999-11-6-25-27	100.50	3111	5.26	0.53	3.71
999-11-6-35-37	100.59	3115	5.88		3.84
999-11-6-55-57	100.79	3121	4.96		3.56
999-11-6-65-67	100.88	3125	5.02		3.81
999-11-6-85-87	101.07	3131	5.00		3.18
999-11-6-85-87	101.07	3131	5.03	0.02	3.39
999-11-6-95-97	101.17	3135	5.41		3.46
999-11-6-115-117	101.36	3141	6.06		3.69
999-11-6-125-127	101.45	3144	5.30		3.32
999-11-6-145-147	101.65	3151	4.97		3.84
999-11-7-5-7	101.74	3154	4.46		3.64
999-11-7-15-17	101.84	3158	4.61		3.80

999-11-7-25-27	101.93	3161	5.07		4.13
999-11-7-35-37	102.03	3165	5.71		3.72
999-11-7-55-57	102.22	3171	4.63		3.84
999-11-7-65-67	102.31	3175	4.90		3.36
999-11-0-15-17	102.50	3183	5.75		4.17
999-12-1-5-7	102.65	3190	5.78		3.17
999-12-1-15-17	102.74	3193	5.14		3.47
999-12-1-55-57	103.12	3210	4.96		3.76
999-12-1-55-57	103.12	3210	4.91	0.03	3.42
999-12-1-65-67	103.21	3214	5.07		3.42
999-12-1-85-87	103.40	3222	5.57		3.74
999-12-1-95-97	103.50	3226	6.06		3.64
999-12-1-105-107	103.59	3230	5.15		3.22
999-12-1-115-117	103.68	3234	5.25	0.07	3.41
999-12-1-115-117	103.68	3234	5.28	0.02	3.41
999-12-1-125-127	103.78	3239	4.83		3.44
999-12-1-135-137	103.87	3242	5.02		3.29
999-12-1-135-137	103.87	3242	4.87	0.11	3.46
999-12-2-5-7	104.06	3251	5.29		3.93
999-12-2-55-57	104.53	3266	5.39		4.46
999-12-2-65-67	104.63	3269	4.88		3.51
999-12-2-105-107	105.01	3279	4.82	0.33	3.52
999-12-3-15-17	105.57	3295	4.99		3.74
999-12-3-45-47	105.85	3304	4.86		3.57
999-12-3-57-59	105.97	3308	4.74	0.08	3.03
999-12-3-65-67	106.04	3311	4.53		3.56
999-12-3-75-77	106.14	3314	4.80		3.47
999-12-4-5-7	106.89	3340	4.69		4.51
999-12-4-45-47	107.27	3353	5.01		2.98
999-12-4-95-97	107.74	3374	4.80	0.22	3.83
999-12-4-105-107	107.84	3378	4.80		4.01
999-12-5-5-7	108.31	3398	4.95		4.53
999-12-5-35-37	108.59	3411	4.35		3.54
999-12-5-55-57	108.78	3419	5.06		3.45
999-12-5-65-67	108.87	3423	4.41		4.23
999-12-5-95-97	109.16	3435	4.69		3.69
999-12-5-105-107	109.25	3439	4.69		3.64
999-12-6-35-37	110.01	3466	4.70		3.63
999-12-6-45-47	110.10	3469	4.15		4.32
999-12-6-95-97	110.57	3485	4.86		4.55
999-12-6-105-107	110.67	3489	5.30	0.25	5.04
999-12-7-15-17	111.23	3508	5.14	0.30	4.46
999-13-1-35-37	112.43	3550	4.61		4.17
999-13-1-55-57	112.62	3556	4.92		4.84
999-13-1-115-117	113.19	3576	5.18	0.02	5.04
999-13-1-125-127	113.28	3579	5.09	0.16	5.03
999-13-2-35-37	113.85	3599	4.90		4.02
