# Supplementary material for: Reduced N2 fixation in the tropical Atlantic Ocean during the warm late Pliocene
Source: Nat Commun. 2026 Aug 5;17:7931. doi: 10.1038/s41467-026-75846-4 (PMC13448806; doi:10.1038/s41467-026-75846-4)
Supplement: Supplementary file 4 [file 41467_2026_75846_MOESM4_ESM.pdf]

Sample lable (if available)	Depth (mcd)	Age (ka)	δ15N *T. sacculifer* (‰ vs. air)	δ15N *T. sacculifer 1SD* (‰ vs. air)	N content *T. sacculifer* (µmol/g)	δ15N *N. dutertrei* (‰ vs. air)	δ15N *N. dutertrei 1SD* (‰ vs. air)	N content *N. dutertrei* (µmol/g)	δ15N *NTC* (‰ vs. air)
662/A17-2-54	147.74	2456	5.08		4.25	5.9	0.29	3.65	5.08
662/A17-2-65	147.85	2458	5.41	0.02	4.32	6.58		3.66	5.41
662/A17-2-75	147.95	2460	5.40		4.25	6.72	0.24	3.36	5.40
662/A17-2-82	148.02	2461	5.78	0.12	4.09	6.94	0.07	3.21	5.78
662/A17-2-92	148.12	2464	6.25	0.22	4.54	7.32	0.21	3.63	6.25
662/A17-2-102	148.22	2466	6.43	0.01	4.84	7.3	0.12	3.92	6.43
662/A17-2-110	148.30	2468	6.80	0.20	4.32	7.58	0.12	4.15	6.80
662/A17-2-125	148.45	2471	7.13	0.09	4.69	7.84	0.01	3.53	7.13
662/A17-2-130	148.50	2472	6.65	0.04	5.02	7.19	0.07	3.60	6.65
662/A17-2-140	148.60	2474	6.46	0.02	4.92	7.28	0.13	3.92	6.46
662/A17-3-2	148.72	2477	5.96	0.45	3.72	7.09	0.25	3.81	5.96
662/A17-3-10	148.80	2479	6.13	0.31	4.19	6.92	0.00	3.43	6.13
662/A17-3-29	148.99	2481	6.00	0.35	4.16	7.03	0.19	3.72	6.00
662/A17-3-48	149.18	2485				6.05		3.75	5.22
662/A17-3-56	149.26	2487				6.44		3.80	5.61
662/A17-3-66	149.36	2488				6.51		3.90	5.68
662/A17-3-73	149.43	2489				5.8		4.74	4.97
662/A17-3-80	149.50	2490				5.43		4.15	4.60
662/A17-3-92	149.62	2493				5.48		4.24	4.65
662/A17-3-100	149.70	2494				5.72		3.75	4.89
662/A17-3-110	149.80	2496				6.42		3.36	5.59
662/A17-4-2	150.22	2505	6.06	0.02	4.07	7.27	0.10	4.46	6.06
662/A17-4-10	150.30	2507	6.55		3.62	7.71	0.17	4.02	6.55
662/A17-4-22	150.42	2509	7.06	0.04	4.18	8.14	0.02	4.17	7.06
662/A17-4-32	150.52	2511	6.63	0.18	3.84	7.98		3.93	6.63
662/A17-4-40	150.60	2513	8.00		4.06	7.77	0.34	4.00	8.00
662/A17-4-52	150.72	2516	6.98		3.74	7.53	0.19	3.96	6.98
662/A17-4-62	150.82	2519	6.10		4.04	7.13	0.20	3.64	6.10
662/A17-4-82	151.02	2524			4.52	6.85		3.62	6.02
662/A17-4-112	151.32	2530			4.68	6.41		4.30	5.58
662/A17-4-120	151.40	2532			4.27	6.95		4.04	6.12
662/A17-4-130	151.50	2535	6.05		4.52	6.74		4.03	6.05
662/A17-5-2.5	151.73	2544	6.40		4.68	6.31		3.95	6.40
662/A17-5-10	151.80	2548			4.27	6.81	0.04	3.92	5.98
662/A17-5-22	151.92	2556	5.59		4.27	6.88	0.14	4.36	5.59
662/A17-5-32	152.02	2563	6.64		4.52	7.02	0.06	3.68	6.64
662/A17-5-42	152.12	2570			4.11	6.59	0.04	4.43	5.76
662/A17-5-52	152.22	2575	6.83	0.17	4.11	7.55	0.10	3.97	6.83
662/A17-5-62	152.32	2579	7.44		3.52	7.69	0.03	4.17	7.44
662/A17-5-70	152.40	2583	6.38	0.32	4.45	7.5	0.05	4.11	6.38
662/A17-5-82	152.52	2588	5.59	0.22	4.31	6.59	0.37	4.31	5.59
662/A17-5-92	152.62	2592	5.82	0.36	4.34	6.33		4.08	5.82
662/A17-5-100	152.70	2596	5.15		4.27			3.77	5.15
662/A17-5-112	152.82	2600				6.41	0.18	3.77	5.58
662/A17-5-120	152.90	2602				6.86	0.21	3.72	6.03
662/A17-5-132	153.02	2607				6.05	0.30	4.42	5.22
662/A17-5-140	153.10	2609				6.03		3.90	5.20
662/A17-6-2	153.22	2613				6.56	0.09	3.90	5.73
662/A17-6-10	153.30	2615	6.53		3.66	6.03	0.17	4.33	6.53
662/A17-6-22	153.42	2617				6.12	0.13	4.21	5.29
662/A17-6-32	153.52	2620	6.15		4.68	7	0.12	3.77	6.15
662/A17-6-43	153.63	2622	5.77		3.81	6.73	0.22	4.07	5.77

662/A17-6-50	153.70	2624	6.23		3.95	6.64	0.10	4.42	6.23
662/A17-6-62	153.82	2627				6.77	0.28	3.38	5.94
662/A17-6-72	153.92	2629				7.35	0.30	4.20	6.52
662/A17-6-82	154.02	2632	7.01	0.17	4.50	7.36	0.33	3.44	7.01
662/A17-6-92	154.12	2634	6.97	0.22	3.95	7.01		4.35	6.97
662/A17-6-102	154.22	2635	5.83	0.37	4.37	7.47	0.36	3.84	5.83
662/A17-6-112	154.32	2637	6.53	0.14	4.54	7.62		3.58	6.53
662/B9-2-103	152.73	2640	7.01	0.34		7.51	0.02		7.01
662/B9-3-3	153.23	2650				6.43		3.56	5.60
662/B9-3-13	153.33	2652	5.82	0.26	3.93	6.41		3.94	5.82
662/B9-3-23	153.43	2653				5.97	0.61	3.85	5.14
662/B9-3-33	153.53	2655				6.14	0.02	4.07	5.31
662/B9-3-43	153.63	2657				6.26	0.14	3.67	5.43
662/B9-3-53	153.73	2659				6.77		3.92	5.94
662/B9-3-63	153.83	2661				6.66	0.14	3.77	5.83
662/B9-3-73	153.93	2663	7.48			8.04		3.36	7.48
662/B9-3-83	154.03	2665	6.98	0.03	4.31	7.52		3.53	6.98
662/B9-3-93	154.13	2667	7.15		4.20	7.18	0.01	3.47	7.15
662/B9-3-103	154.23	2669	7.15	0.02	3.91	6.91	0.09	3.58	7.15
662/B9-3-113	154.33	2671	6.83	0.01	4.25	6.66	0.13	4.07	6.83
662/B9-3-123	154.43	2673	5.87	0.22	4.06	6.87	0.13	3.43	5.87
662/B9-3-133	154.53	2673	6.91	0.24	3.76	7.26	0.33	3.66	6.91
662/A18-1-42	155.62	2674	5.72	0.12	3.97	6.91	0.18	3.69	5.72
662/B9-3-143	154.63	2675	6.01	0.23	4.16	6.95	0.10	5.01	6.01
662/A18-1-52	155.72	2676	6.50		4.18	8.02		3.61	6.50
662/B9-4-3	154.73	2677	7.83	0.09	3.97	8.19		3.70	7.83
662/A18-1-62	155.82	2678	7.27	0.37	3.41	7.85	0.08	3.60	7.27
662/B9-4-13	154.83	2678	7.36	0.26	3.99	6.95		3.91	7.36
662/A18-1-72	155.92	2680	7.41	0.17	4.10	7.64	0.11	3.82	7.41
662/B9-4-23	154.93	2681	6.76		4.57	7.32	0.16	3.88	6.76
662/A18-1-82	156.02	2682	6.65	0.13	3.54	7.15	0.22	4.05	6.65
662/B9-4-33	155.03	2682	6.58	0.01	4.10	7.27	0.14	3.92	6.58
662/A18-1-92	156.12	2684	6.51	0.02	3.49	7.29	0.26	3.86	6.51
662/B9-4-43	155.13	2686	6.35	0.13	4.65	7.05		3.85	6.35
662/B9-4-53	155.23	2686	7.11		4.54	7.32		3.90	7.11
662/A18-1-111	156.31	2687	6.00	0.04	3.43	6.92	0.17	3.53	6.00
662/B9-4-63	155.33	2688				6.78	0.21	3.40	5.95
662/A18-1-124	156.44	2689	6.61						6.61
662/A18-1-132	156.52	2689	5.79		4.64	6.24		4.06	5.79
662/A18-2-2.5	156.73	2693							
662/A18-2-22.5	156.93	2696	5.78	0.02	3.39	7.15	0.14	3.83	5.78
662/A18-2-31.5	157.02	2697	6.86		3.92	7.74	0.14	3.93	6.86
662/A18-2-43	157.13	2699	6.85		4.58	7.29	0.29	4.83	6.85
662/A18-2-54	157.24	2701	5.70	0.01	3.60	6.97	0.26	3.76	5.70
662/A18-2-62	157.32	2703	5.25	0.04	3.58	6.46		3.44	5.25
662/A18-2-71.5	157.42	2704	5.71		3.48	7.34		3.70	5.71
662/A18-2-81.5	157.52	2706	5.80		3.56	7.78		3.91	5.80
662/A18-2-91	157.61	2708	6.31		3.54				6.31
662/A18-2-101.5	157.72	2709	5.89		4.01				5.89
662/A18-2-141	158.11	2716	5.92			6.52	0.41	4.06	5.92
662/A18-3-14	158.34	2720				5.82	0.10		4.99
662/A18-3-22	158.42	2721	5.52		4.28	6.31	0.35		5.52
662/A18-3-32	158.52	2723	5.41			6.42	0.07	3.67	5.41
662/A18-3-43	158.63	2725	5.65		3.80	6.89	0.16	4.03	5.65
662/A18-3-52	158.72	2727	5.67		3.80	6.87		4.37	5.67
662/A18-3-60	158.80	2728	6.86	0.07	3.90	7.91	0.11	3.77	6.86

662/A18-3-70	158.90	2730	7.16			7.5		4.07	7.16
662/A18-3-81	159.01	2732	7.00	0.06	4.08	7.66		4.33	7.00
662/A18-3-90	159.10	2733	6.54	0.00	3.88	7.31	0.00	4.14	6.54
662/A18-3-100	159.20	2735	6.18		4.27	7.25	0.14	3.69	6.18
662/A18-3-111	159.31	2738							
662/A18-3-122	159.42	2740				7.14		3.82	6.31
662/A18-3-133	159.53	2743							
662/A18-3-141	159.61	2745	5.95		4.61				5.95
662/A18-4-2	159.72	2748	6.49		4.38				6.49
662/A18-4-12	159.82	2751				7.28		4.48	6.45
662/A18-4-22	159.92	2754	6.09		4.47	7.11	0.05	4.21	6.09
662/A18-4-32	160.02	2756	5.92	0.08	4.68	7.12		4.19	5.92
662/A18-4-42	160.12	2759	5.77		3.58	7.37	0.07	4.28	5.77
662/A18-4-52	160.22	2761	5.85		3.65	6.95	0.00	4.03	5.85
662/A18-4-60	160.30	2763			3.73	6.91	0.00	3.90	6.08
662/A18-4-70	160.40	2765	5.97		3.73	7.3		4.17	5.97
662/A18-4-81	160.51	2768			3.98	7.41			6.58
662/A18-4-93	160.63	2770	6.34	0.13	3.75	7.4		3.82	6.34
662/A18-4-101	160.71	2772	7.22		3.98	7.63		4.05	7.22
662/A18-4-111	160.81	2775	6.37	0.01	4.76	7.34	0.22	4.33	6.37
662/A18-4-122	160.92	2777				7.23		4.21	6.40
662/A18-4-132	161.02	2779	5.91	0.03	3.93				5.91
662/A18-4-141	161.11	2782	5.46	0.07	4.72	7.04	0.01	4.36	5.46
662/A18-5-4	161.24	2784	5.98	0.09	5.28	6.43	0.30	4.50	5.98
662/A18-5-14	161.34	2786	6.03	0.13	5.07	7.22	0.16	4.14	6.03
662/A18-5-24	161.44	2788	7.26	0.32	4.74	7.73		4.24	7.26
662/A18-5-34	161.54	2791	6.63	0.12	4.74	7.37	0.36	4.40	6.63
662/A18-5-44	161.64	2793	6.21		4.59	6.11	0.02	4.42	6.21
662/A18-5-54	161.74	2795	6.09	0.03	3.87	6.63	0.04	4.10	6.09
662/A18-5-64	161.84	2799	6.35	0.02	3.97	7.05	0.01	4.01	6.35
662/A18-5-74	161.94	2802	5.86		3.81				5.86
662/A18-5-84	162.04	2804				6.49		4.20	5.66
662/A18-5-104	162.24	2810	5.41	0.18	3.97	6.73	0.25	4.14	5.41
662/A18-5-114	162.34	2812	5.69		3.93	6.93		3.77	5.69
662/A18-5-124	162.44	2815	5.90		3.97	6.8	0.00	4.21	5.90
662/A18-5-134	162.54	2818	6.07	0.17	4.01	6.47	0.03	4.30	6.07
662/A18-5-144	162.64	2821	6.22		3.95	6.66		4.02	6.22
662/A18-6-2	162.72	2824	6.50	0.09	4.11	7.17	0.20	4.18	6.50
662/A18-6-12	162.82	2828	6.41	0.07	4.33	7.46		4.11	6.41
662/A18-6-22	162.92	2831	7.02	0.1	4.32	7.76		4.34	7.02
662/A18-6-32	163.02	2834	6.95	0.25	4.47	8.05		3.78	6.95
662/A18-6-42	163.12	2837				7.28	0.11	4.17	6.45
662/A18-6-52	163.22	2839	6.48	0.27	3.76	7.13	0.20	3.90	6.48
662/A18-6-60	163.30	2841	6.34		3.26	6.83	0.25	4.72	6.34
662/A18-6-74	163.44	2844	5.95		3.75			3.67	5.95
662/A18-6-92	163.62	2848	5.17		4.00	7.11	0.24	3.67	5.17
662/A18-6-102	163.72	2850	5.51		3.91	6.82	0.22	4.11	5.51
662/A18-6-112	163.82	2853	6.35	0.08	4.08	7.55		4.40	6.35
662/A18-6-122	163.92	2855	5.95	0.14	3.73	7.25	0.02	3.62	5.95
662/A18-6-132	164.02	2857	6.01	0.11	3.47	7.19		4.27	6.01
662/A18-6-142	164.12	2860	6.60		3.78	7.19		3.59	6.60
662/A18-7-2	164.22	2863	6.84	0.17	4.41	7.46	0.35	4.19	6.84
662/A18-7-12	164.32	2866	6.38	0.22	4.12			4.23	6.38
662/A18-7-22	164.42	2869	6.19		3.95	7.32		4.23	6.19
662/B10-4-12	164.32	2880				7.37		4.02	6.54
662/B10-4-24	164.44	2883	5.42	0.13	3.91	6.93	0.15	3.82	5.42

662/B10-4-34	164.54	2886	5.35		3.88	7.22	0.36	4.17	5.35
662/B10-4-42	164.62	2889	5.49		3.79	6.84		3.74	5.49
662/A19-1-2	164.72	2892				6.98	0.10	3.64	6.15
662/B10-4-52	164.72	2895	6.06		3.66	7.24	0.22	4.03	6.06
662/B10-4-62	164.82	2897	6.64		3.78	7.56		4.09	6.64
662/A19-1-22	164.92	2900	6.86	0.16	3.87	7.43	0.06	4.27	6.86
662/B10-4-72	164.92	2903.2	5.67	0.41	3.98	6.54		4.34	5.67
662/B10-4-82	165.02	2911.5	6.50		4.57	7.41		4.22	6.50
662/B10-4-92	165.12	2917.25	6.60		4.64	6.86	0.14	4.23	6.60
