# Supplementary material for: Reduced N2 fixation in the tropical Atlantic Ocean during the warm late Pliocene
Source: Nat Commun. 2026 Aug 5;17:7931. doi: 10.1038/s41467-026-75846-4 (PMC13448806; doi:10.1038/s41467-026-75846-4)
Supplement: Supplementary file 5 [file 41467_2026_75846_MOESM5_ESM.pdf]

mcd (m)	age (LR04)	O18adj
117.31	1550	3.71
117.39	1553.4	3.55
117.49	1557.6	3.7
117.61	1562.6	3.66
117.7	1566	3.63
117.79	1568.8	3.94
117.91	1571.5	4.11
117.99	1573.3	4.08
118.09	1575.6	4.35
118.22	1578.5	4.17
118.3	1580.6	3.96
118.39	1583.7	3.83
118.51	1588	3.78
118.59	1590.8	3.81
118.66	1593.3	3.76
118.81	1598.6	3.72
118.89	1601.4	3.64
118.99	1604.9	3.58
119.11	1608.8	3.82
119.2	1610.8	3.95
119.29	1612.8	3.81
119.41	1615.4	3.72
119.49	1617.1	3.88
119.59	1619.1	3.7
119.71	1621.3	3.68
119.8	1622.8	3.48
119.89	1624.3	3.48
120.01	1626.4	3.89
120.31	1631.7	3.7
120.61	1637.4	3.52
120.79	1640.8	3.72
120.91	1643	3.9
120.99	1644.5	4.06
121.09	1646.4	4
121.21	1648.8	4.47
121.3	1650.7	4.41
121.3	1650.7	4.26
121.39	1652.6	4.49
121.39	1652.6	4.39
121.51	1655.2	4.34
121.51	1655.2	4.33
121.59	1656.9	4.41
121.59	1656.9	4.16
121.66	1658.4	4.12
121.81	1661.5	3.94
121.89	1663.2	3.79
121.99	1665.1	3.99
122.11	1667.2	3.93
122.2	1668.8	4.01

122.29	1670.3	3.9
122.49	1673.7	3.78
122.71	1677.4	3.77
122.8	1678.9	3.63
122.89	1680.4	3.46
123.01	1682.5	3.59
123.09	1683.8	3.54
123.16	1685	3.32
123.31	1687.5	3.56
123.49	1690.6	4.22
123.49	1690.6	4.16
123.61	1692.6	4.02
123.61	1692.6	3.9
123.7	1694.2	4.1
123.7	1694.2	3.96
123.79	1695.8	3.78
123.91	1698.3	3.52
123.99	1700	3.9
124.09	1702.6	4.02
124.21	1705.9	3.89
124.3	1708.5	3.96
124.51	1714.4	3.78
124.59	1716.7	3.69
124.59	1716.7	3.59
124.66	1718.6	3.23
124.81	1723.1	3.55
124.89	1725.9	3.36
124.99	1729.5	3.49
125.11	1734	3.6
125.2	1737.8	3.44
138.29	2271.4	3.4
138.29	2271.4	2.93
138.45	2275.6	3.83
138.53	2277.8	3.69
139.35	2296	3.76
139.53	2299.1	3.57
139.65	2301.1	3.46
139.73	2302.5	3.34
139.8	2303.7	3.17
139.95	2306.2	3.62
140.03	2307.6	3.4
140.25	2310.7	3.75
140.43	2312.7	3.81
140.55	2314.1	4.08
140.63	2315	3.67
140.73	2316.1	4.18
140.85	2317.4	3.8
141.03	2319.5	3.94
141.15	2320.8	4.22
141.23	2321.7	3.91

141.3	2322.5	3.78
141.63	2326.2	4.17
141.75	2327.6	3.7
141.93	2329.9	3.86
142.05	2331.9	3.57
142.13	2333.2	3.64
142.23	2335.1	3.42
142.35	2337.7	3.12
142.53	2342.7	3.23
142.65	2345.5	3.01
142.73	2347.3	3.13
142.8	2348.9	3.24
142.95	2352.1	3.91
143.03	2353.6	3.57
143.13	2355.5	4.13
143.25	2357.5	4.02
143.43	2360.4	3.89
143.63	2362.7	3.87
143.73	2363.9	3.8
143.73	2363.9	3.79
143.85	2365.8	3.77
144.03	2368.9	3.7
144.15	2370.9	3.68
144.23	2372.2	3.56
144.45	2376	3.3
144.53	2377.3	3.23
144.63	2379	3.3
144.75	2381	3.25
144.93	2384.1	3.13
145.05	2386.3	3.18
145.13	2387.8	3.21
145.23	2389.6	3.29
145.35	2392.1	3.47
145.53	2395.9	3.53
145.65	2398.5	3.45
145.73	2400.2	3.5
145.8	2401.7	3.56
145.95	2405	3.58
146.03	2406.8	3.48
146.13	2409.5	3.4
146.25	2413.6	3.33
146.35	2417.2	3.21
146.45	2420.7	3.34
146.57	2424.6	3.53
146.57	2424.6	3.3
146.68	2427.3	3.55
146.68	2427.3	3.77
146.78	2429.4	3.89
146.86	2430.9	4.16
146.86	2430.9	4.2

146.96	2432.6	4.15
147.08	2434.4	4.26
147.08	2434.4	3.98
147.26	2436.4	4.12
147.38	2437.8	4.09
147.46	2438.7	4.08
147.56	2439.8	4.1
147.68	2441.2	4.25
147.86	2443.2	4.08
147.86	2443.2	4.09
148.06	2445.5	3.96
148.13	2446.2	3.77
148.26	2447.8	3.88
148.36	2449.3	3.77
148.46	2451	3.88
148.58	2453.1	3.51
148.76	2456.5	3.65
148.88	2458.8	3.25
148.88	2458.8	3.17
149.56	2473.9	3.32
149.62	2475.1	3.46
149.79	2478.5	3.86
149.84	2479.4	3.76
149.99	2482	3.96
150.08	2483.5	4.12
150.26	2486.5	4.17
150.38	2488.6	4.09
150.46	2489.9	3.91
150.46	2489.9	3.83
150.56	2491.6	3.88
150.68	2493.9	3.67
150.85	2497.5	3.09
151.28	2506.6	3.32
151.36	2508.3	3.55
151.46	2510.4	3.5
151.58	2513.1	3.61
151.76	2517.7	4.21
151.76	2517.7	4.16
151.88	2521	4.33
151.96	2523	4.08
152.06	2525.3	4.05
152.18	2528	3.96
152.36	2532.1	3.91
152.56	2538.4	3.56
152.63	2541	3.64
152.78	2548.4	3.23
152.86	2554	3.32
152.96	2561.1	3.17
153.08	2569.1	3.11
153.26	2577.7	2.97

153.38	2583.3	3.23
153.46	2586.7	3.23
153.56	2591	3.43
153.68	2595.5	3.48
153.86	2602.3	3.66
154.06	2609.2	3.54
154.13	2611.2	3.54
154.28	2615.1	3.27
154.46	2619.2	3.42
154.46	2619.2	3.15
154.58	2621.9	3.34
154.58	2621.9	3.2
154.76	2626	3.07
154.76	2626	2.99
154.76	2626	3.23
154.88	2628.7	3.15
154.96	2630.5	3.16
155.06	2632.7	3.27
155.18	2635.4	3.34
155.36	2639.2	3.42
155.46	2641.1	3.91
155.54	2642.6	3.79
155.54	2642.6	3.93
155.63	2644.1	3.77
155.63	2644.1	3.91
155.74	2645.9	3.71
155.74	2645.9	3.77
155.84	2647.6	3.68
155.84	2647.6	3.6
155.95	2649.5	3.55
156.05	2651.2	3.5
156.15	2652.9	3.28
156.35	2656.3	3.32
156.45	2658	2.99
156.55	2659.8	3.06
156.65	2661.9	3.1
156.75	2664	3
156.85	2666.1	3.03
156.95	2668.2	3.09
157.05	2670.1	3.2
157.13	2671.6	3.47
157.19	2672.8	2.96
157.31	2675	2.95
157.49	2678.4	2.99
157.61	2680.7	3.27
157.69	2682.2	3.35
157.79	2683.8	3.7
157.91	2685.9	3.86
158.09	2688.9	3.86
158.21	2690.9	4

158.21	2690.9	3.84
158.29	2692.3	3.73
158.37	2693.6	3.67
158.37	2693.6	3.68
158.51	2696	3.56
158.59	2697.4	3.5
158.81	2701.1	3.51
158.99	2704.1	3.56
159.11	2706.2	3.81
159.19	2707.5	3.66
159.29	2709.2	3.85
159.41	2711.3	3.91
159.59	2714.3	3.97
159.71	2716.3	3.92
159.79	2717.7	3.91
159.84	2718.5	3.79
159.84	2718.5	3.74
160.01	2721.4	3.65
160.01	2721.4	3.68
160.09	2722.8	3.66
160.09	2722.8	3.68
160.18	2724.3	3.57
160.31	2726.5	3.54
160.49	2729.5	3.52
160.61	2731.7	3.37
160.69	2733.3	3.19
160.79	2735.4	3.19
160.91	2738	3.06
160.91	2738	3.13
161.09	2742.1	3
161.21	2745.5	3.02
161.29	2747.8	3.05
161.37	2750	3.03
161.51	2754	3.09
161.59	2756.2	3.15
161.69	2758.6	3.15
161.81	2761.3	3.55
161.99	2765.4	3.39
161.99	2765.4	3.43
162.11	2768.1	3.34
162.19	2769.9	3.21
162.29	2772.1	3.29
162.41	2774.8	3.35
162.59	2778.9	3.08
162.71	2781.6	3.05
162.79	2783.4	3.05
162.79	2783.4	3.15
162.87	2785.2	3.06
163.01	2788.4	3.24
163.09	2790.2	3

163.09	2790.2	3.12
163.19	2792.4	3.02
163.19	2792.4	3.11
163.19	2792.4	3.08
163.31	2795.4	3.15
163.49	2800.5	3.51
163.49	2800.5	3.58
163.61	2803.9	3.61
163.69	2806.1	3.64
163.79	2809	3.6
163.91	2812.3	3.57
163.91	2812.3	3.49
164.09	2817.4	3.37
164.21	2820.8	3.22
164.29	2823.7	2.86
164.37	2826.5	3.14
164.51	2831.4	2.96
164.59	2834.2	3.08
164.81	2839.4	3.13
164.99	2843.4	3.39
165.11	2846.2	3.41
165.19	2848	3.52
165.29	2850.2	3.4
165.41	2852.9	3.43
165.59	2857.1	3.27
165.71	2860.4	3.19
165.79	2862.7	3.06
166.01	2868.9	3.35
166.09	2871.4	3.21
166.29	2878.5	3.32
166.39	2882	3.21
166.5	2885.4	3.39
166.59	2887.9	3.34
166.69	2890.7	3.27
166.79	2893.6	3.3
166.89	2896.4	3.11
166.99	2899.2	3.07
167.09	2902	3.08
167.19	2904.9	3.18
167.29	2907.7	3.13
167.4	2910.8	3.23
167.44	2911.9	3.29
167.56	2915.3	3.23
167.64	2917.5	3.18
167.74	2921	3.36
167.86	2925.2	3.58
168.04	2931.5	3.69
168.16	2934.9	3.31
168.24	2937.6	3.08
168.31	2940.1	3.04

168.31	2940.1	2.74
168.46	2945.6	2.86
168.54	2948.6	2.69
168.64	2952.3	2.86
168.76	2956.8	3.1
168.94	2963.6	2.95
169.06	2968.1	3.06
169.06	2968.1	3.15
169.14	2971.1	3.32
169.24	2974.9	3.5
169.36	2979.4	3.3
169.54	2986.2	3
169.66	2990.7	2.99
169.74	2993.7	3.12
169.96	3001.6	3.38
170.04	3003.8	3.43
170.14	3006	3.41
170.14	3006	3.57
170.26	3008.8	3.57
170.44	3012.8	3.31
170.56	3015.9	3.51
170.64	3018.2	3.37
170.74	3021.7	3.22
170.86	3026	3.32
171.04	3032.4	3.08
171.04	3032.4	3.07
171.16	3036.9	3.14
171.16	3036.9	3.01
171.24	3039.9	3.14
171.31	3042.5	3.23
171.46	3048.2	3.47
171.54	3051.2	3.2
171.64	3054.9	2.96
171.76	3059.4	2.81
171.94	3066.2	2.83
172.06	3070.7	3.16
172.14	3073.7	3.04
172.24	3077.5	2.96
172.36	3082	2.97
172.36	3082	2.93
172.54	3088.8	3.03
172.66	3093.3	3.08
172.74	3096.3	3.15
172.81	3098.9	3.15
172.96	3104.6	3.09
173.04	3107.6	3.12
173.14	3111.3	3.06
173.26	3115.8	3.2
173.56	3126.5	3.26
173.64	3129.3	3.3

173.74	3132.8	3.28
173.86	3137.1	3.4
174.04	3143.4	3.33
174.16	3147.6	3.1
174.24	3150.5	2.83
174.31	3152.9	2.72
174.46	3158.2	2.87
174.54	3161	2.96
174.64	3164.3	3.04
174.76	3167.1	3.11
174.94	3171.2	3.01
175.06	3173.9	3.14
175.14	3175.7	3.02
175.24	3177.9	3.11
175.36	3180.6	3.15
175.54	3185.7	2.97
175.66	3189.1	2.84
175.74	3191.3	2.91
175.81	3193.3	3.02
175.96	3197.5	2.99
176.14	3202.6	2.89
176.42	3209.3	3.11
176.52	3211.6	3.08
176.62	3213.9	3
176.7	3215.7	3.06
176.82	3218.4	3.04
176.92	3220.6	2.99
177.02	3222.9	3.21
177.12	3225.1	3.2
177.22	3227.4	3.26
177.3	3229.2	3.01
177.42	3231.9	2.96
177.52	3234.2	3.02
177.62	3236.4	3.18
177.72	3239.2	2.93
177.8	3242	2.93
177.89	3245.2	2.92
177.99	3248.7	2.94
178.09	3252.2	2.98
178.19	3255.1	3.02
178.29	3257.7	2.98
178.39	3259.9	3.17
178.49	3262.2	3.19
178.59	3264.3	3.22
178.59	3264.3	3.16
178.69	3266.4	3.27
178.69	3266.4	3.2
178.79	3268.5	3.06
178.89	3270.6	3.15
178.99	3272.8	3.1

179.09	3274.9	3.16
179.19	3277	3.05
179.28	3278.9	3.17
179.28	3278.9	3.05
179.44	3282.3	3.15
179.44	3282.3	3.15
179.52	3284	2.95
179.62	3286.1	3.68
179.74	3288.6	3.61
179.92	3292.4	3.71
180.04	3295	3.63
180.04	3295	3.67
180.12	3296.7	3.72
180.22	3298.8	3.47
180.34	3301.3	4.07
180.52	3305.3	3.43
180.52	3305.3	3.4
180.64	3308.2	3.05
180.72	3310.5	3.29
180.79	3312.7	3.17
180.94	3318	3.08
180.94	3318	2.89
181.02	3320.8	2.95
181.12	3324.4	3.05
181.24	3327.8	3.21
181.42	3332.9	3.27
181.54	3336.3	3.1
181.72	3341.3	3.46
181.84	3345.1	3.19
181.93	3348.2	3.34
182.02	3351.4	3.2
182.14	3355.6	3.12
182.22	3358.5	3.04
182.29	3360.9	3.06
182.44	3366.2	3
182.52	3369	3.06
182.62	3371.9	3.31
182.74	3374.6	3.26
182.83	3376.6	3.22
182.83	3376.6	3.39
182.92	3378.7	3.29
183.04	3381.4	3.18
183.04	3381.4	3.3
183.12	3383.2	3.28
183.34	3388.4	2.96
183.43	3390.9	3.1
183.64	3396.8	3.09
183.79	3401.1	3.03
183.94	3406.1	3.02
184.02	3408.9	2.93

184.02	3408.9	2.82
184.12	3412.4	2.84
184.12	3412.4	2.92
184.24	3416.7	2.77
184.33	3419.8	2.9
184.42	3423	3.07
184.54	3427.3	3.11
184.62	3430.1	2.86
184.72	3433.6	2.67
184.84	3437.3	2.77
184.96	3440.7	2.77
185.44	3454.2	2.86
185.52	3456	2.81
185.62	3458.3	2.84
185.74	3461	2.94
185.83	3463	3.07
185.92	3465	3.13
186.04	3467.7	3.01
186.12	3469.5	3.01
186.22	3472.6	3.03
186.34	3477.1	2.8
186.43	3481	2.68
186.52	3485.2	2.83
186.64	3490.9	2.98
186.72	3494.6	2.94
186.79	3497.9	2.58
186.94	3504.9	2.73
187.02	3508.6	2.78
187.12	3512.6	3.01
187.44	3522.3	3.09
187.52	3524.1	3.07
187.62	3526.4	3.15
187.74	3529.8	3.02
187.83	3533.1	3.16
187.92	3536.6	3.01
188.04	3541.7	3.02
188.12	3545.3	2.96
188.22	3550	2.93
