# Supplementary material for: Reduced N2 fixation in the tropical Atlantic Ocean during the warm late Pliocene
Source: Nat Commun. 2026 Aug 5;17:7931. doi: 10.1038/s41467-026-75846-4 (PMC13448806; doi:10.1038/s41467-026-75846-4)
Supplement: Supplementary file 6 [file 41467_2026_75846_MOESM6_ESM.pdf]

Supplementary Dataset S4: Summary of δ^{15}N comparison between *T. sacculifer* and *G. ruber* for Site 999

Climate state	n (samples)	Mean δ15N (‰)	SD	Mean offset (‰)	Offset SD	R^2	p-value (regression)	p-value (offset t-test)	Bootstrap 95% CI (offset)
Glacial	77	5.54	0.77	-0.73	0.44	0.7	5.44E-15	0.045	-0.84 to -0.61
Interglacial	66	5.1	0.7	-0.52	0.55	0.5	1.64E-07	0.045	-0.68 to -0.37
