# Supplementary material for: Reduced N2 fixation in the tropical Atlantic Ocean during the warm late Pliocene
Source: Nat Commun. 2026 Aug 5;17:7931. doi: 10.1038/s41467-026-75846-4 (PMC13448806; doi:10.1038/s41467-026-75846-4)
Supplement: Supplementary file 7 [file 41467_2026_75846_MOESM7_ESM.pdf]

nature portfolio

Peer Review File

Reduced N2 fixation in the tropical Atlantic Ocean during the Warm Late Pliocene

Corresponding Author: Dr Maayan Yehudai

This file contains all reviewer reports in order by version, followed by all author rebuttals in order by version.

Version 0:

Reviewer comments:

Reviewer #1

(Remarks to the Author)
Yehudai et al present foram bound nitrogen isotopes (FB-d15N) at 2 sites in the low-latitude Atlantic during the Pliocene-Pleistocene Transition (PPT) to infer changes in nitrogen cycle dynamics during that period, in comparison to the last 160 ka. Based on the presented new records, they drew 2 conclusions. Firstly, N2 fixation was reduced during the late Pliocene compared to the late Quaternary, due to global water-column denitrification. Secondly, orbit-scale variabilities in N2 fixation emerged after intensification of Northern Hemispheric Glaciation (iNHG), due to sea level fluctuations.
The data presented is of high quality, and I largely agree with the authors on the data interpretation. However, currently the discussion is not laid out in a convincing and coherent way, especially for the first conclusion. Below, I provide summarised comments on the two discussion sections first, and then some detailed comments, many of which are also related to the layout of the discussion.

WTA N2 fixation during the late Pliocene
I agree that the smaller FB-d15N offset between sites 662 and 999 supports weakened N2 fixation in the WTA. However, before this conclusion can be drawn, it should be established that FB-d15N at site 662 can be interpreted as the global mean pycnocline level. To establish this, I have the following questions. What are the hydrographic and biogeochemical settings of this site at present and during the last 160 ka? How these conditions may or may not change during the PPT in comparison, especially considering changes in upwelling intensity before and after iNHG? How can these changes or a lack of changes be supported by the data?
The authors directly state that the lower N2 fixation during the late Pliocene is driven by lower excess P supply, without introducing possible controlling factors of this process (including excess P supply!) in the introduction or previously in the discussion. Alternative causes of the lowered N2 fixation were later dismissed (L169-178), a paragraph which I feel would be much better placed before presenting their preferred case.
It is not explained why benthic denitrification is excluded as the cause of the lowered excess P supply (L159-L164), until L244.

Detailed comments
L37: Contradictory to L84.
L53: A reference is needed.
L62-70: The authors discussed the potential of the studied period as an analog to future conditions, but this point is not revisited in the discussion/conclusion. Either remove this point here or discuss how the PPT reconstruction can inform us about future N cycle changes.
L68: Some might agree that mid-Pliocene is the ‘best’ analog, some might not. Consider rephrasing.
L74: Given the context mentioned above, it may be helpful to introduce potential contributors to N2 fixation changes.
L78: Introduce the notation of N isotope here might be better.
L80: (15N and 14N, respectively)
L86-8: This sentence is not clear. Consider breaking it into 2.
L88: Unclear what ‘Its’ refers to.
L88-90: What is stated here is correct, but the relation between sea level changes and benthic denitrification should be established before this statement.
L92: Isn’t N2 fixation also a controller of the mean ocean d15N?
L94: There is no context for mentioning the Site 1012 study. Adding something like “previous understanding about the PPT

N cycle changes was based on bulk sediment d15N" could help. This also helps to introduce FB-d15N.
L108: Changes in FB-d15N at Site 662 are interpreted as the mean ocean pycnocline changes, a point which was argued for previously for the last 160 ka (Auderset et al 2024). This is not necessarily true for the PPT and needs to be demonstrated using data.
L115: No need to shorten T. sacculifer or N. dutertrei.
L116 and elsewhere: Add standard deviations to the associated means.
L119: Not clear which period is discussed here.
L122: What is the age of MIS G10?
L129: There are too many jumps in introducing NTC-d15N. What's the hydrographic condition at site 662 that makes it suitable for reflecting the mean ocean thermocline d15N? Why d15N of a mixed layer species, T. sacculifer, can represent that of the thermocline? Why can d15N of T. sacculifer and N. dutertrei, after adjusting for the interspecies difference be treated as equivalent to each other?
L131 and Methods The way NTC d15N is calculated is not clearly described.
L133: Reporting average and sd is better than the range.
L137: It looks like that FB-d15N at site 999 displays orbital-scale variability, but not that at site 662.
L150: Only the lower FB-d15N difference between WTA and ETA indicates lower N2 fixation.
L151: EEA or ETA? Need to be consistent.
L163: Unclear what 'it' refers to.
L167: I don't think data from this site can be used to demonstrate lower denitrification, if the similarity between FB-d15N at site 662 and Equatorial Pacific sites has already been used to argue that d15N at site 662 reflects mean ocean pycnocline change. The argument would be circular.
L173-178: Is the FB-d15N offset between sites rather than the FB-d15N at site 999 related to N2 fixation, and thus be compared with Fe fluxes and SST?
L184: Need to explain why water upwelled in EEA is from Pacific.
L186-7: FB-d15N variations at site 999 are consistent with those at site 662 during the last 160 ka is not consistent with what was reported in Auderset et al 2024.
L191-193: This sentence is awkward here.
L194: More background (some is provided in Fig. 1 caption and L261) is needed in the main text for discussing the potential link between EEA upwelling and WTA N2 fixation.
L216: The high coherence started around 3.3 Ma based on Fig. 3e.
L223-4: The references are not correctly numbered.
L226: More details are needed for this calculation. It is not clear
L244-7: It is like a cliff hanger to me to find out why benthic denitrification did not cause lower N2 fixation. The discussion needs to be reorganized.
L238-40: Not clear which site is discussed here.
L250: Better to say that you provided d15N and inferred N2 fixation changes from it.
L252-3: Need to stress that this finding is in the WTA.
L264-5: Isn't sea level also regulated N2 fixation during the late Pleistocene as reported in Auderset et al 2024 and Ren et al 2017, a point recognized in L222-4?
L274: Better to tell readers that N2 fixation is strong at this site in the modern ocean.
L283: This statement is inaccurate in that it ignores the contribution of N2 fixation to mean pycnocline d15N.
L289-297: Some of the information regarding calculating NTC-d15N can be relocated to be after analytical methods, or be removed after moving supplement texts to methods.
Fig.1: Consider adding maps showing modern ocean nitrate concentration, and if possible, d15N.
Fig .2:
What's the time window roughly corresponding to 21 points? Does it differ between the last 160 ka and the PPT?
Need to provide more details about records from sites 849 and 806: what's the species?
I recommend showing d18O of the two sites here and highlighting G or IG.
I recommend distinguishing published data and new data in panel a at least, and in other (supplementary) figures if possible.
Fig 4: Making this figure wider can help to better show details in the record.
Highly recommend showing a sea level reconstruction in addition to the shelf area%.

Supplement texts: I recommend moving these to Methods.
S4: "Second, although GIA exerts a spatially heterogenous influence on continental shelf area in the late Pleistocene (e.g.: 11), this influence was likely more muted at low- to mid-latitudes during the Pliocene due to the smaller size and loading footprint of the Northern Hemisphere ice sheets."
I am not an expert on GIA adjustment, but this statement seems to contradict with Raymo et al (2011) ref 9 in supplementary material.

Reviewer #2

(Remarks to the Author)
Overview and general recommendation:

Yehudai et al. present high-resolution foraminifera-bound N stable isotope data from two IODP cores in the Western Tropical Atlantic and Eastern Equatorial Atlantic, to evaluate the drivers of nitrogen cycling, and particularly N-fixation, over a major global climate transition. They compare this to similar research from the last 160 kyr, to assess how drivers of N-fixation have varied with wider climate factors. The paper is generally well written, with robust interpretations and discussion, evidenced

through appropriate statistical and data analysis. The incorporation of modelled shelf area changes is particularly rigorous for an isotope-based study. There are minor spelling, grammar and figure call out errors throughout, so this will need thorough checking and updating. I also think there could also be improvements to the flow and clarity in places to aid a reader who does not have as strong a familiarity with the region, time period, and acronyms used. But the dataset produced is excellent and the overall findings are well outlined.

My only concern is that the paper's methods, results and broader conclusions are notably similar to previous studies from similar author groups. For example, Straub et al. 2013 (Nature) presents closely related material in the introduction, methods and discussion, which lessens the sense of novelty. While this paper identifies the source of excess P as being different during the PPT (from sea-level driven shelf derived source) compared to the last 160 kyr (upwelling), the wider significance of this difference is not well highlighted. Similarly, although also on modern sediments, the role of shelf-derived phosphorus as an important control on N fixation has also been previously evaluated using foram-d15N by Auderset et al., 2024 (Paleoceanography and Paleoclimatology). I am inclined to think the manuscript would benefit from revisions that more clearly highlight the novel insights from examining this time period specifically, particularly the shelf modelling, which to my knowledge has not been undertaken in previous studies.

The authors mention in the abstract that the Pliocene is a geologic analog for future warming however this is not discussed in any detail within the manuscript; what about the findings here are relevant to the future? If none, this should be removed from the abstract. Alternatively/in addition, as the authors present the data from the Pacific cores within their figure 2, it would be useful to add discussion about how this new Atlantic data fits within a broader global context through this major climate transition.

Overall, the study is well designed and presented and I have no major concerns with the methods, results or conclusions, but I think they need to better highlight the specific novelty of this work in the discussion for publication in Nature Communications. I therefore recommend for publication with minor revisions.

Other Comments:

I struggled a little with flow of the narrative where there are switches between date ranges and named periods (e.g. iNHG vs G10 vs ~2.8 Ma) – improved consistency between what is used in figures and text would be helpful. For example Figure 2 and 4 have NHG written and then MIS G10 associated with the iNHG in the caption. It would be helpful to someone who doesn't work specifically in this time period if the same terms were used throughout.

Consider adding the last paragraph of the Core Location section (lines 282-284) into the end of the introduction as this is a clearer explanation of how the sites link together.

Data from IODP sites 806, 849 and 1012 are presented in Figure 2, but are not discussed in the text. There is mention of 1012 in the introduction, but no discussion is made of those datasets in relation to the new data presented here, so it is not clear why they are included. I think this panel of the figure should either be removed or if not, could be an interesting addition to discuss the new findings from Atlantic in the broader context of the MOC and how similar sea level declines and potential P influx in the Pacific basin may contribute to d15N data presented from other papers?

Line 289: The authors indicate there is good correlation between G. ruber and Tssac (which looks to be mostly true from Fig S4).However, Straub et al., (2013), as cited by authors, indicates that there is variation in the species-specific offset between G.ruber and G. sacculifer during glacials and climate transitions in their FB-d15N study on the same site. Similarly in Ren et al., (2012), it states: "The δ15N of G. ruber is slightly lower than the other species...this difference is generally greatest between 16 and 25 ka...roughly similar to what has been observed in the Caribbean, where the maximal species difference also occurs at the LGM [Ren et al., 2009]." Could the authors comment on whether this is observed in their data, for example if they split their current Figure S4 data into glacial and interglacial datasets, does this alter the correlation significantly? In the modern, my understanding is that Tsacc lives slightly lower in the water column than G. ruber and therefore could be exposed to a more enriched N, as well as potentially calcifying later in the season (which could also enrich N signal). Could the authors comment on the potenthis and whether it could contribute to the higher values in this authors PPT record compared to the 160ka record which averages G. ruber and Tssac?

Lines 169-178: Authors discuss a lack of correlation between the FB-d15N and dust but do not appear to undertake this test statistically (as they have done for the SST). Could similar or even more simple correlative tests be used to confirm this more objectively?

Minor Comments:

Line 37: Add (N) after nitrogen?

Line 45: typo "reduced" rather than "reduce"

Line 64-64: low latitude with and without hyphen used

Line 78: add reference(s)?

Line 82: ODZ already defined

Line 116: Authors suggest elevation of ~2 ‰ but that the mean shifts from 5.4 to 4.5 ‰ (0.9 ‰ difference). Could the author clarify where they got 2 ‰ from?

Line 117-120: Cited Fig 2b, should be 2a?

Line 120: Notable increase not drop?

Line 118: Glacial-interglacial variability is discussed and cited to Fig 2, but this is not clear on the figure, I think in part as the eye is drawn to the smoothing spline (which is helpful). I wonder whether citing to Figure 4 where the glacial bands are highlighted would be more useful.

Line 132: the NTC part of FB-δ15NNTC notation is not used in the figure, figure caption or consistently in discussion (e.g. line 162). Perhaps remove?

Fig 2: panel b is cited before panel a, should these be flipped?

Fig S1. Caption typo – duplicated "on the"

Current references to SI S4 which I don't think has been included in this submission.

Version 1:

Reviewer comments:

Reviewer #1

(Remarks to the Author)
I reiterate that I agree with the data interpretation of the authors and acknowledge the potential contribution of the work. I still find the revised version not clearly organized, and at places arguments are not coherent.

Introduction:
Information regarding N2 fixation and denitrification is mixed. Given that the key finding of the paper is about N2 fixation, I highly recommend gathering all the scattered pieces of information about N2 fixation, put them together, and clarify the gap of our knowledge regarding this process.

Section "Drivers of WTA N2 fixation since the Pliocene":
The interpretation of the site 662 record that is driven by global water-column denitrification is mixed with the interpretation of the d15N offset between Sites 999 and 662 that is driven by local N2 fixation. I am also surprised not to be able to find a statement like "less excess P due to weaker global water-column denitrification led to lowered N2 fixation in the WTA" in the paragraph from L175-189 that connects the 2 observations from the dataset. I strongly recommend the authors using their data to explain these points separately and improve the flow of reasoning here.

Detailed comments
L42-44: better to specify that this process is observed locally in the WTA.
L86-91: flow here can be improved. You discussed water-column denitrification and then sediment denitrification and finally came back to water-column denitrification.
L116: do we know what percentage of global N2 fixation occurs in the WTA? If yes, it is worth mentioning.
L128: what is this "shorter" timescale compared to?
L130: No need to add "(hereafter T. sacculifer)". Genus names are shortened after the first appearance conventionally.
L151 move "throughout the PPT" after "dutertrei".
L154: standard deviation of the interspecies offset should be shown, and such an error should be incorporated to the derived thermocline d15N. Also, how is this interspecies 15N difference compared to those observed in previous work?
L160-1: If Site 999 d15N is lower than site 662, they are not similar. Better show averages with standard deviations.
L170-173: Only a higher d15N at site 662 can indicate that d15N supply to the tropical Atlantic was higher during the Pliocene.
L173-174: The observation that the d15N of nitrate supplied to the Tropical Atlantic was higher during the Pliocene does not support lower N2 fixation there.
L183: More explanation is needed here regarding the possible contribution of sedimentary denitrification to excess P production globally. If water column denitrification is associated with a d15N fractionation of ~-20 permil and the thermocline d15N is ~-6 permeil, global sedimentary denitrification should be more important for the denitrification and thus excess P production.
L187-189: What are "these observations"? This sentence seems to be saying previous data can demonstrate that Atlantic N2 fixation was lowered during the Late Pliocene, which is not what the authors meant I assume.
L207-209: this sentence is vague.
L211: While I agree that denitrification in the Pacific produces high-excess-P thermocline waters that can raise the global

average thermocline excess P, I do not think you can track “(high-excess-P) Pacific-sourced waters entering the Atlantic via the Southern Ocean”. This is not supported by modern hydrographic data.
L211-213: this piece of information is also needed in the previous section on the overall Pliocene N2 fixation changes.
L214-216: this sentence would work better if you rephrase it to something like “the upwelling control on N2 fixation can be supported by the relationships of d15N with alkenone MAR ”.
L225: change “lowest” to “lower than interglacials”.
L233: rephrase “rich in excess P”
L247-249: The observation that sea level controlled N2 fixation in SCS and tropical Atlantic during the late Pleistocene cannot be used to prove that such a process occur during the PPT in these regions or in the WTA.
L279: remove “and potentially global”. The observation is regional.
L286-289: similar to a previous comment, I wonder how the global sedimentary denitrification would change and how it would affect global excess P and thus N2 fixation.
L301-304: specify this occurs in the Atlantic.
L358: this is Materials and Methods section.
Fig. 2:
In the legend, it is better to provide the species used for the records, especially for Site 662 record that is derived from 2 species.
Standard errors for d15N measurements and derived thermocline d15N at 662 need to be shown.
It would be helpful to highlight PPT in the figure.

Fig. 3a,4a: specify these are benthic d18O data.
Fig. 5:
In d, better to say early Pleistocene glaicals to be consistent.
Double check processes in panels c &d.

S1: this section or a simplified version of it should be included the Methods.

(Remarks on code availability)

Reviewer #2

(Remarks to the Author)
Overview and general recommendation
I thank the authors for their careful consideration of my review and thoughtful responses. This version of the manuscript is much more coherent and highlights differences with existing work more clearly. I commend them for their additional work and data analysis to address some of my comments and particularly like the addition of Figure 5 which nicely visualizes the key findings. I have highlighted a few additional, minor comments, which I do not feel will significantly change the manuscript, but might enhance these small areas. I would recommend this manuscript for publication.

Paragraph at Line 248: As you outline how records from the late Pleistocene reinforce your results, would there be any relevance in discussing changes related to the Mid-Pleistocene transition, and closure of the Bering Strait/exposure of the Beringian continental shelf, which are proposed to have suppressed North Pacific upwelling (through enhanced NPIW) in glacial periods and caused elevated d15N records in the Bering Sea and subarctic Pacific? Would your mechanism of shelf exposure and P supply fit with this as well [making this a more global hypothesis]? There are several bulk d15N records from IODP Expedition 323 that cover this interval by authors including Knudson, Ravelo, Kender and Worne, as well as older published records from ODP Site 882. Maybe not relevant/beyond scope, but just a thought.

Lines 75-79: A very long and clunky sentence, could this be edited/split into two?

Fig S4. Could the authors put the r2 and p values onto the panels for each plot to visualize and evidence that there is no correlation. While it does not look like there is for most panels, it would be interesting to know the correlation between FB-d15N and N content of G.ruber which looks to be slightly positive? If so, some explanation of how this impacts results would be needed.

Fig S7. Color differences between light and dark teal/pink are very hard to see. Could the authors change the color/symbology to show a clearer difference between this study and the referenced studies, to make sure to consider the requirements for visually impaired readers who may struggle with the current color format. Also, the caption implies both panels of data have been smoothed but I don’t think the second panel of teal/pink data has been smoothed here, so caption may need to be adjusted, or the smoothed data replaced onto the figure.

(Remarks on code availability)

Version 2:

Reviewer comments:

Reviewer #1

(Remarks to the Author)
I still think that the data and science presented in this manuscript is high-quality, valid, and interesting. But I did not see substantial improvement in this version from the last one, and thus cannot support publication of the work in the current form, for the same reasons I gave in the last round of review.

I do not think my pervious comments on the Section "Drivers of WTA N2 fixation since the Pliocene" has been addressed. Here is my previous comment:
"The interpretation of the site 662 record that is driven by global water-column denitrification is mixed with the interpretation of the d15N offset between Sites 999 and 662 that is driven by local N2 fixation."
In this current version, the arguments for the use of Dd15N between Sites 662 and 999 for inferring N2 fixation changes are still not well laid out.
For example, the authors state that "the WTA-EEA FB-δ15N differences (ΔFB-δ15N; Fig. S9) to reflect the strength of N2 fixation in the tropical North Atlantic" on L178. A key premise of this statement is that "Regions where the main pycnocline outcrops, such as at Site 66253, provide an opportunity to monitor its nitrate δ15N, for comparison with records such as that of Site 999.", which does not appear until L196. Then L199 is repeating the statement at L178.
Also, the authors confusingly mentioned the excess P change at L189-90 but only explain the potential role of excess P to N2 fixation at L200, and then at L202 introduce potential drivers of N2 fixation (including excess P).
In the last review, I "recommend the authors using their data to explain these points separately". I reiterate this recommendation. It would be much clearer if you first discuss results from Site 662, compare it with Pacific data to establish that it reflects global thermocline changes (including site 999). Then you move on to discuss what the Dd15N tell us.

While I acknowledge that language should not be used to judge the quality of the manuscript, I am listing the large number of grammatical errors and confusing sentences in this 3rd version I read as a major concern. These errors make it difficult to follow the author's arguments. I encourage the authors to carefully proofread the manuscript, including the material and method section.

Here is an incomplete list of sentences need revision due to grammatical errors
L65: remove "biological" before "sources"
L72-4
L81-3
L83: in in
L85 rephrase 14N-nitrate
L85-6
L 98 change "and" to "because"
L103-5
L120 NEARLY
L165
L223: Unclear what "they" refer to.
L223: Southern-sourced and ventilated in the Southern Ocean, repetitive.

Minor comments.
L68: 3-3.3 Ma is the Mid Pliocene Warm Period. In L118, you are referring 3.6 Ma as Late Pliocene.
L112-4: This is repeating L59-63 and L89-91
L120 and 123: giving locations of these cores in the main text is the common practice.
L125: Do the authors mean the pycnocline is below the thermocline at this site? Perhaps stick to thermocline or pycnocline throughout the manuscript (see again eg, L158, L330), if it is not important to distinguish these two.
L126: this is misleading. Only Site 999 covers 1 ma of time.
L141-2: Firstly, I am not sure what you are comparing the PPT IG d15N with, the average of the last 160 ka or the IG values of the last 160 ka? Secondly, this is also a strange start to describe your G-IG scale results. It is better to focus on your data first then move on to comparison with published data. Thirdly, it should be mentioned that the glacial d15N are higher somewhere in this paragraph.
L164: I don't disagree with the authors that d15N of these two species can be used to reflect the thermocline signal, now that I am reading this manuscript the third time. But this is a point that will puzzle many in the field that know about the habitats of these species. Providing some explanations for your readers here would be great.
L165: Check the numbers here. The numbers are inconsistent with your arguments. This sentence is also unclearly written.
L177: Not sure this is true. The smoothed PPT d15N level at Site 999 is comparable to that during the last 50 ka according to Fig.2
L225-229: this is relevant information but seems to be awkwardly positioned here.

Fig 2: The duration of PPT is not correctly shown

(Remarks on code availability)

Reviewer #2

(Remarks to the Author)
I am satisfied that authors have responded to all comments.

(Remarks on code availability)

Version 3:

Reviewer comments:

Reviewer #1

(Remarks to the Author)
I am happy with revisions made to the manuscript and can recommend it for publication.

(Remarks on code availability)

Open Access This Peer Review File is licensed under a Creative Commons Attribution 4.0 International License, which permits use, sharing, adaptation, distribution and reproduction in any medium or format, as long as you give appropriate credit to the original author(s) and the source, provide a link to the Creative Commons license, and indicate if changes were made.
In cases where reviewers are anonymous, credit should be given to 'Anonymous Referee' and the source.
The images or other third party material in this Peer Review File are included in the article's Creative Commons license, unless indicated otherwise in a credit line to the material. If material is not included in the article's Creative Commons license and your intended use is not permitted by statutory regulation or exceeds the permitted use, you will need to obtain permission directly from the copyright holder.
To view a copy of this license, visit https://creativecommons.org/licenses/by/4.0/

Please find below a response to the reviewers' comments.

We numbered the comments in Italic and placed our response below each comment.

Reviewer #1 (Remarks to the Author):

Yehudai et al present foram bound nitrogen isotopes (FB-d15N) at 2 sites in the low-latitude Atlantic during the Pliocene-Pleistocene Transition (PPT) to infer changes in nitrogen cycle dynamics during that period, in comparison to the last 160 ka. Based on the presented new records, they drew 2 conclusions. Firstly, N2 fixation was reduced during the late Pliocene compared to the late Quaternary, due to global water-column denitrification. Secondly, orbit-scale variabilities in N2 fixation emerged after intensification of Northern Hemispheric Glaciation (iNHG), due to sea level fluctuations. The data presented is of high quality, and I largely agree with the authors on the data interpretation. However, currently the discussion is not laid out in a convincing and coherent way, especially for the first conclusion. Below, I provide summarised comments on the two discussion sections first, and then some detailed comments, many of which are also related to the layout of the discussion.

We appreciate the reviewer's positive feedback and acknowledgements of our findings. We have revised the discussion section of the manuscript and believe its coherency has improved significantly (see section XX of the revised manuscript).

1. WTA N2 fixation during the late Pliocene
   I agree that the smaller FB-d15N offset between sites 662 and 999 supports weakened N2 fixation in the WTA. However, before this conclusion can be drawn, it should be established that FB-d15N at site 662 can be interpreted as the global mean pycnocline level.

We have outlined this in the materials and methods ('Core location and hydrography settings' section) and lines 112-114 of the revised main text to make that case stronger. Due to the word limit, we briefly summarized the reasoning in the main text and referred the reader to the methods sections for further details.

We believe ODP Site 662 is considered a good representation of the global pycnocline $\delta^{15}N$ for several key reasons. Firstly, for its location in the equatorial Atlantic, a region where the pycnocline is well-defined and is relatively stable. Secondly, the $\delta^{15}N$ values from ODP Site 662 align with the mean nitrate $\delta^{15}N$ of the global ocean pycnocline, which is approximately 6.3‰ (Fripiat et al., 2023). *In addition, records from ODP Site 662 $\delta^{15}N$*

values over the last interglacial period have been suggested to represent the global pycnocline $\delta^{15}N$ (Auderset et al., 2024).

2. To establish this, I have the following questions. What are the hydrographic and biogeochemical settings of this site at present and during the last 160 ka?

In the low latitude modern ocean, Ekman wind-driven upwelling systems such as the Eastern Equatorial Atlantic, housing ODP site 662, the hydrography conditions enable the outcropping of the main pycnocline (Brandt et al., 2023). *That is, the sub-thermocline water nitrate is brought to the surface* (Marconi et al., 2017). *Given the complete consumption of nitrate in the modern equatorial Atlantic surface, FB-$\delta^{15}N$ at equatorial Atlantic ODP Site 662 should reflect the $\delta^{15}N$ of pycnocline nitrate. In addition, Auderset et al., 2024 compared FB-$\delta^{15}N$ data from various sites, including ODP Site 662, and found similar glacial and interglacial $\delta^{15}N$ values in the Equatorial Atlantic, suggesting a consistent isotopic composition (See figure 5b below, taken from Auderset et al.; 2024, also see materials and methods ('Core location and hydrography settings' section) and lines 112-114 of the revised manuscript).*

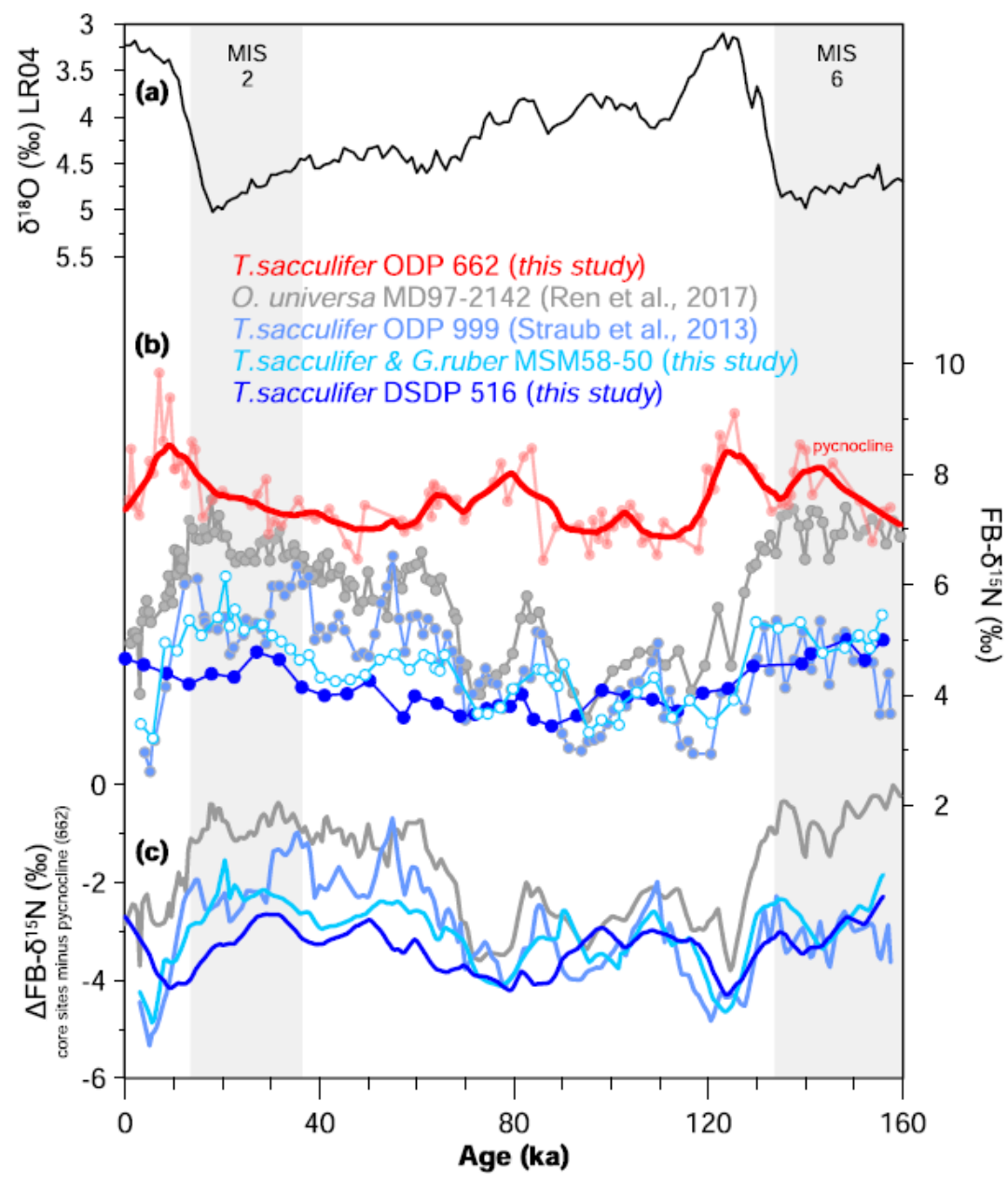

3. How these conditions may or may not change during the PPT in comparison, especially considering changes in upwelling intensity before and after iNHG?

The intensity of upwelling at Site 662 has varied over time, influenced by climatic events such as the intensification of Northern Hemisphere glaciation (iNHG). After 2.7 Ma upwelling intensity in the equatorial Atlantic increased during glacial period leading to enhanced nutrient supply to the surface waters (Lawrence et al., 2013). Conversely, interglacial periods were associated with reduced upwelling and a more stratified ocean structure after the iNHG. However, we consider that the consumption of nutrients at site 662 was complete even during glacial times. Therefore, FB-$\delta^{15}N$ should still reflect the mean pycnocline values. Over million year time scales, the site reflects the large-scale nitrate reservoir rather than strong local signals. Because the mean $\delta^{15}N$ of oceanic nitrate is primarily controlled by the balance of nitrogen fixation and denitrification, with denitrification exerting the stronger leverage, global decreases in denitrification would lower the mean $\delta^{15}N$. An overall lower denitrification intensity across the PPT should be expressed as an overall lower FB-$\delta^{15}N$ at Site 662, reflecting the lighter isotopic composition of the global nitrate pool. This is discussed in more detail now in materials and methods ('Core location and hydrography settings' section) and lines 112-114 and 162-173 of the revised manuscript.

4. How can these changes or a lack of changes be supported by the data?

The relatively lower global water-column denitrification rate revealed by Rafter et al., 2025, would have driven the pycnocline $\delta^{15}N$ value towards lower values, an expectation that is consistent with our FB-$\delta^{15}N$ data throughout the PPT. We have clarified this further in lines 162-173 of the revised manuscript and emphasized it further in Fig. S6.

5. The authors directly state that the lower N2 fixation during the late Pliocene is driven by lower excess P supply, without introducing possible controlling factors of this process (including excess P supply!) in the introduction or previously in the discussion.

Corrected. We have moved the introduction to excess P to the introduction. See lines 59-66 of the revised manuscript.

6. Alternative causes of the lowered N2 fixation were later dismissed (L169-178), a paragraph which I feel would be much better placed before presenting their preferred case.

We thank the reviewer for this suggestion. The discussion section has been substantially revised and streamlined in the new version, which we believe improves the overall flow and

clarity. However, we chose to retain the structure where the preferred interpretation is introduced first, followed by evaluation of alternative explanations. We feel that this organization more effectively conveys the main conclusion and provides clearer context for why competing hypotheses were rejected (see lines 164-199 of the revised text).

7. It is not explained why benthic denitrification is excluded as the cause of the lowered excess P supply (L159-L164), until L244.

Corrected. This is now further discussed in lines 166-167 and also lines of the revised manuscript.

Detailed comments

8. L37: Contradictory to L84.

Corrected. See lines 89-99 of the revised manuscript.

9. L53: A reference is needed.

We added a reference to Gruber (2008). See line 53 of the revised manuscript.

10. L62-70: The authors discussed the potential of the studied period as an analog to future conditions, but this point is not revisited in the discussion/conclusion. Either remove this point here or discuss how the PPT reconstruction can inform us about future N cycle changes.

We maintain that this is a point we would like to keep in the paper and have added a "closure" to this point in the 'conclusions' section (lines 250-278 of the revised manuscript).

11. L68: Some might agree that mid-Pliocene is the 'best' analog, some might not. Consider rephrasing.

Changed "best" to "potential". See lines 39 and 73 of the revised manuscript.

12. L74: Given the context mentioned above, it may be helpful to introduce potential contributors to N2 fixation changes.

We thank the reviewer for this suggestion. To maintain the flow of the manuscript and avoid overloading the Introduction with details, we chose to introduce potential

contributors to N_2 fixation changes at a later stage, where they are discussed in more detail. However, as the reviewer suggested in earlier comments, we added details about how excess P is crucial for maintaining N_2 fixation globally (See lines 50-66 of the revised manuscript), which provide better background to build on in the discussion sections. If the Editor considers it essential, we are happy to incorporate an additional brief mention in the Introduction.

13. L78: Introduce the notation of N isotope here might be better.

Done. See line 83 of the revised manuscript.

14. L80: (15N and 14N, respectively)

Corrected. See lines 84-86 of the revised manuscript.

15. L86-8: This sentence is not clear. Consider breaking it into 2.

Split. See lines 92-95 of the revised manuscript.

16. L88: Unclear what 'Its' refers to.

Corrected. See line 95 of the revised manuscript.

17. L88-90: What is stated here is correct, but the relation between sea level changes and benthic denitrification should be established before this statement.

Corrected. See lines 92-94 of the revised manuscript.

18. L92: Isn't N2 fixation also a controller of the mean ocean d15N?

This is now further discussed in lines 102-104 of the revised manuscript.

Both denitrification and N2 fixation matter for the mean ocean nitrate but in a steady state they affect it in different ways:

Denitrification removes fixed N with a relatively stronger isotopic fractionation effect (~20-30 ‰). This preferential removal of ^{14}N would immediately raise the residual mean ocean $\delta^{15}N$. N_2 fixation adds new nitrogen to the ocean with a very light isotopic signature ($\delta^{15}N \approx -1$‰), however the fractionation factor for this process is quite small, meaning that it mainly dilutes the heavier nitrate pool rather than actively fractionating it. This means that the impact on the mean $\delta^{15}N$ value is weaker per unit flux change in denitrification, as seen in the diagram below. Because the isotopic offset between fixed-N inputs (≈ −1 ‰)

and the residual nitrate pool (≈ +5 ‰) is smaller than the effective fractionation of denitrification, changes in denitrification exert the stronger influence on the mean $\delta^{15}N$ of the ocean (Altabet, 2007; Sigman and Fripiat, 2019).

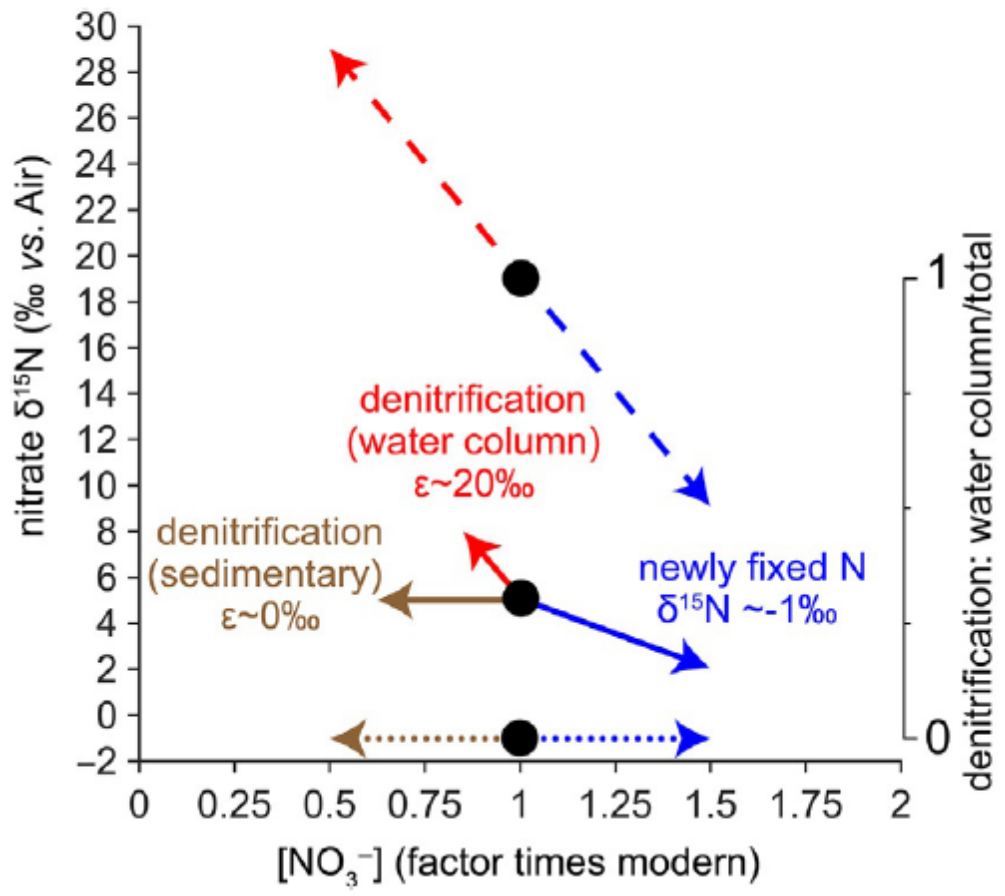

19. L94: There is no context for mentioning the Site 1012 study. Adding something like "previous understanding about the PPT N cycle changes was based on bulk sediment d15N" could help. This also helps to introduce FB-d15N.

Since Rafter et al. (2025) is now published, we have decided to remove the bulk $\delta^{15}N$ data from 1012 and therefore do not mention this site explicitly in our revised text.

20. L108: Changes in FB-d15N at Site 662 are interpreted as the mean ocean pycnocline changes, a point which was argued for previously for the last 160 ka (Auderset et al 2024). This is not necessarily true for the PPT and needs to be demonstrated using data.

This is now further discussed in the revised materials and method section (lines 292-315 of the revised text). Also see response to comments 2-4 above.

21. L115: No need to shorten T. sacculifer or N. dutertrei.

Corrected. See lines 125-141 and throughout the revised manuscript.

22. L116 and elsewhere: Add standard deviations to the associated means.

Corrected. See lines 125-132 of the revised manuscript.

23. L119: Not clear which period is discussed here.

Corrected. See line 132-135 of the revised manuscript.

24. L122: What is the age of MIS G10?

Added. See line 136 of the revised manuscript.

25. L129: There are too many jumps in introducing NTC-d15N. What's the hydrographic condition at site 662 that makes it suitable for reflecting the mean ocean thermocline d15N?

We have now consolidated everything to one primary point in the manuscript and expand on it in the materials and methods. See lines 142-150 of the revised manuscript. Also see response to comments 4-7 above.

26. Why d15N of a mixed layer species, T. sacculifer, can represent that of the thermocline?

We discuss this extensively in SI S1 which now moved to the materials and methods section and is referred to in line 145 of the revised manuscript. The reason is that although T. sacculifer calcifies in the mixed layer, its food source organic matter is exported from the thermocline nitrate reservoir, which dominates the $\delta^{15}N$ signal of exported material. Thus, its FB-$\delta^{15}N$ tracks the isotopic composition of the thermocline nitrate pool (Ren et al., 2012, 2009) .

27. Why can d15N of T. sacculifer and N. dutertrei, after adjusting for the interspecies difference be treated as equivalent to each other?

N. dutertrei lives deeper (upper thermocline) and thus incorporates the $\delta^{15}N$ of thermocline nitrate more directly, but paired core-top and down-core records show that the two species are offset by a nearly constant interspecific difference. After correcting for this offset, their $\delta^{15}N$ values can be treated as equivalent proxies of the thermocline signal (Ren et al., 2017, 2012; Smart et al., 2018). *See 'Calculation of thermocline/ nutricline - FB-$\delta^{15}N$ from ODP Site 662' section of the materials and methods, lines 356-377 of the revised manuscript).*

28. L131 and Methods The way NTC d15N is calculated is not clearly described.

Corrected. See lines 356-377 of the revised manuscript and previous comment response..

29. L133: Reporting average and sd is better than the range.

Corrected. See lines 141-152 of the revised manuscript.

30. L137: It looks like that FB-d15N at site 999 displays orbital-scale variability, but not that at site 662.

This is correct, the PPT records from both sites show the dominance of obliquity driven variability after ~2.8 Ma but they do not change in the same manner, which calls for another mechanism to explain the variations observed at Site 999 over this period. We have now also added Fig. S8 to the revised supplementary data to describe the obliquity dominant variability at Site 662. See lines 151-159 of the revised manuscript.

31. L150: Only the lower FB-d15N difference between WTA and ETA indicates lower N2 fixation.

Corrected. See lines 163-166 of the revised manuscript.

32. L151: EEA or ETA? Need to be consistent.

Corrected. See line 64 of the revised manuscript.

33. L163: Unclear what 'it' refers to.

Corrected. See lines 175-178 of the revised manuscript.

34. L167: I don't think data from this site can be used to demonstrate lower denitrification, if the similarity between FB-d15N at site 662 and Equatorial Pacific sites has already been used to argue that d15N at site 662 reflects mean ocean pycnocline change. The argument would be circular.

Because the mean $\delta^{15}N$ of oceanic nitrate is primarily controlled by the balance between nitrogen fixation and denitrification, with denitrification exerting the dominant influence, a global decrease in denitrification would lower the mean $\delta^{15}N$. Consequently, a reduction in denitrification intensity across the PPT should manifest as a decrease in FB-$\delta^{15}N$ at Site 662, reflecting the lighter isotopic composition of the global nitrate pool, which aligns with our observations. Importantly, the resemblance we refer to is not in the absolute FB-$\delta^{15}N$ values at Pacific sites compared to Site 662, but in the pattern of change over the PPT compared to the last 160 ka: specifically, whether $\delta^{15}N$ would shift more positive or negative in response to changes in global water column denitrification.

We clarify this line of reasoning further in lines 178-183 of the revised manuscript.

35. L173-178: Is the FB-d15N offset between sites rather than the FB-d15N at site 999 related to N2 fixation, and thus should be compared with Fe fluxes and SST?

Both records provide complementary information. The offset gives an idea of the residual (local) $\delta^{15}N$ once the changes in the global pycnocline have been accounted for. In any case, the use of one or the other record does not change our interpretation. We have clarified this in the new version of the manuscript (lines 184-193).

36. L184: Need to explain why water upwelled in EEA is from Pacific.

Explained. See lines 205-207 of the revised manuscript.

37. L186-7: FB-d15N variations at site 999 are consistent with those at site 662 during the last 160 ka is not consistent with what was reported in Auderset et al 2024.

Corrected. Over the last 160 ka, the variations in FB-$\delta^{15}N$ at Site 999 are consistent with variations in alkenone data and not the FB-$\delta^{15}N$ from Site 662. See lines 207-210 of the revised manuscript.

38. L191-193: This sentence is awkward here.

Rephrased. See lines 213-215 of the revised manuscript.

39. L194: More background (some is provided in Fig. 1 caption and L261) is needed in the main text for discussing the potential link between EEA upwelling and WTA N2 fixation.

This information is also provided in the previous paragraph to the one mentioned in this comment (lines 203-207) and we have now also expanded on this in the introduction (see lines 61-63 of the revised manuscript).

40. L216: The high coherence started around 3.3 Ma based on Fig. 3e.

Corrected. See lines 235-238 of the revised manuscript.

41. L223-4: The references are not correctly numbered.

Corrected. See lines 239-242 of the revised manuscript.

42. L226: More details are needed for this calculation. It is not clear

Corrected. See lines 242-246 of the revised manuscript.

43. L244-7: It is like a cliff hanger to me to find out why benthic denitrification did not cause lower N2 fixation. The discussion needs to be reorganized.

We appreciate the reviewer's observation. In the revised version, we have clarified and expanded the discussion to explicitly explain why benthic denitrification alone cannot account for the overall lower N_2 fixation observed during the Pliocene. Specifically, we now note that benthic denitrification is expected to ***increase****, not decrease, under higher sea levels, and that the simpler explanation for the long-term reduction in N_2 is lower global water-column denitrification, which would have reduced the oceanic excess P inventory. The revised discussion has been reorganized accordingly to clearly separate the effects of water-column and benthic denitrification (Lines 239-258 of the revised manuscript).*

44. L238-40: Not clear which site is discussed here.

Corrected. See lines 254-258 of the revised manuscript.

45. L250: Better to say that you provided d15N and inferred N2 fixation changes from it.

Corrected. See line 261 of the revised manuscript.

46. L252-3: Need to stress that this finding is in the WTA.

Corrected. See line 264 of the revised manuscript.

47. L264-5: Isn't sea level also regulated N2 fixation during the late Pleistocene as reported in Auderset et al 2024 and Ren et al 2017, a point recognized in L222-4?

While sea-level fluctuations have been shown to influence N_2 fixation during the late Pleistocene (Auderset et al., 2024; Ren et al., 2017)*, our study extends this relationship back to the Pliocene–Pleistocene Transition and demonstrates, for the first time, that sea-level–driven modulation of N_2 fixation was already active under early glacial conditions. We note this connection in the revised text (Lines 276-289).*

48. L274: Better to tell readers that N2 fixation is strong at this site in the modern ocean.

Corrected. See lines 296-297 of the revised manuscript.

49. L283: This statement is inaccurate in that it ignores the contribution of N2 fixation to mean pycnocline d15N.

Corrected. See lines 322-323 of the revised manuscript.

50. L289-297: Some of the information regarding calculating NTC-d15N can be relocated to be after analytical methods, or be removed after moving supplement texts to methods.

It is now added to the Materials and methods section. See lines 363-384 of the revised manuscript.

51. Fig.1: Consider adding maps showing modern ocean nitrate concentration, and if possible, d15N.

Added. See Figs. 1 and S1 of the revised manuscript.

52. Fig .2:
What's the time window roughly corresponding to 21 points? Does it differ between the last 160 ka and the PPT?

It corresponds to ~105 kyr. See line 627 of the revised manuscript.

53. Need to provide more details about records from sites 849 and 806: what's the species?

We have referenced the paper which was recently published, where all these details can be found (Rafter et al., 2025). See lines 178-183 and 629 on the revised manuscript.

54. I recommend showing d18O of the two sites here and highlighting G or IG.

We appreciate the suggestion. However, since $\delta^{18}O$ data are already shown and discussed in other figures, adding them here would be redundant and not essential for the specific point this figure illustrates. We therefore prefer to keep the figure as is, unless the editor advises otherwise.

55. I recommend distinguishing published data and new data in panel a at least, and in other (supplementary) figures if possible.

Corrected. See line 623-635 of the revised manuscript.

56. Fig 4: Making this figure wider can help to better show details in the record. Highly recommend showing a sea level reconstruction in addition to the shelf area%.

Corrected. See line 651 of the revised manuscript.

57. Supplement texts: I recommend moving these to Methods.

We moved some of it into the Materials and methods section but chose to leave some of the text in the SI.

58. S4: "Second, although GIA exerts a spatially heterogenous influence on continental shelf area in the late Pleistocene (e.g.: 11), this influence was likely more muted at low- to mid-latitudes during the Pliocene due to the smaller size and loading footprint of the Northern Hemisphere ice sheets."
I am not an expert on GIA adjustment, but this statement seems to contradict with Raymo et al (2011) ref 9 in supplementary material.

Corrected. See SI-S3 of the revised manuscript.

****

Reviewer #2 (Remarks to the Author):
Overview and general recommendation:

59. Yehudai et al. present high-resolution foraminifera-bound N stable isotope data from two IODP cores in the Western Tropical Atlantic and Eastern Equatorial Atlantic, to evaluate the drivers of nitrogen cycling, and particularly N-fixation, over a major global climate transition. They compare this to similar research from the last 160 kyr, to assess how drivers of N-fixation have varied with wider climate factors**. The paper is generally well written, with robust interpretations and discussion, evidenced through appropriate statistical and data analysis.**

We thank the reviewer for their thoughtful and encouraging consideration of our paper.

60. The incorporation of modelled shelf area changes is particularly rigorous for an isotope-based study.

We again thank the reviewer for acknowledging the innovative thinking and novelty of our approach.

61. There are minor spelling, grammar and figure call out errors throughout, so this will need thorough checking and updating.

The manuscript was edited thoroughly and all typos and spelling/grammar errors are now corrected.

62. I also think there could also be improvements to the flow and clarity in places to aid a reader who does not have as strong a familiarity with the region, time period, and acronyms used.

We thank the reviewer for this comment and have made the following changes to the manuscript in order to increase flow and clarity to ease reader experience.

63. But the dataset produced is excellent and the overall findings are well outlined.

We again thank the reviewer for highlighting the high quality of our findings.

64. My only concern is that the paper's methods, results and broader conclusions are notably similar to previous studies from similar author groups. For example, Straub et al. 2013 (Nature) presents closely related material in the introduction, methods and discussion, which lessens the sense of novelty.

We thank the reviewer for raising this important point. We have now made a clearer distinction throughout the paper of how it is different from previous studies and highlighted the novelty of our work (See lines 112-122 and 162-258 of the revised manuscript). We also added a new figure to make the new findings of our work clearer (New Fig. 5). While our study builds on previous work (e.g., Straub et al., 2013; Auderset et al., 2024), it provides several new contributions.

Most importantly, it is the first to apply FB-δ^{15}N to the Pliocene-Pleistocene Transition (3.6–1.8 Ma), in the Atlantic, extending beyond the late Quaternary and modern focus of earlier studies and over more than a million years. This data shows for the first time that N_2 fixation was lower during the Pliocene warm period (Fig. 2a). This is not an obvious conclusion based on the mechanism proposed in our previous papers such as Straub et al. (2013) and recent modern observations (Jung et al., 2025). For example, during the

Pliocene, sea level was higher so one may have expected higher N_2 fixation based on the mechanisms proposed by Auderset et al 2024. In addition, based on earlier (Straub et al., 2013) and recent studies (Jung et al., 2025), the expectation would have been an increase in N_2 fixation with the onset of equatorial upwelling changes at 2.8 Ma, which is again not consistent with our observations.

Our records reveal a distinct mechanism: reduced Pliocene N_2 fixation can only be explained by the diminished global denitrification that reduced the supply of excess P. Because of the reduced excess P in pycnocline waters, the mechanism proposed by Straub et al. 2013 (equatorial upwelling), did not have any effect on N_2 fixation. In contrast, on orbital timescales, N_2 fixation was paced by obliquity-driven, sea-level–controlled changes in shelf-derived benthic denitrification

These findings therefore provide novel insights into nitrogen cycle dynamics under warm climate states and during the onset of Northern Hemisphere glaciation. These points are now highlighted throughout the revised version of the manuscript (see for example lines 227- 238 and 280-289)).

65. While this paper identifies the source of excess P as being different during the PPT (from sea-level driven shelf derived source) compared to the last 160 kyr (upwelling), the wider significance of this difference is not well highlighted.

Thank you for this helpful comment. We agree that clarifying the role of different excess P sources is important. In our study, we distinguish between long-term and glacial-interglacial controls on N_2 fixation. The long-term trend across the PPT reflects changes in global water-column denitrification, which regulates the global oceanic excess P inventory. Reduced denitrification under warm Pliocene conditions would have lowered excess P and thus suppressed global N_2 fixation. After ~2.8 Ma, the glacial-interglacial variability emerging in N_2 fixation was primarily driven by sea-level changes that modulated benthic denitrification on continental shelves, altering the local excess P supply to surface waters. Together, these results show that N_2 fixation was influenced by distinct processes operating on different timescales, with global control by water-column denitrification and regional modulation by shelf exposure. This is further detailed in lines XX-XX of the revised manuscript.

In addition, we have now also revised the discussion and conclusions to highlight more clearly that while during the last 160 kyr N_2 fixation was controlled by upwelling in the Eastern Equatorial Atlantic, during the PPT, excess phosphorus supply was primarily controlled by sea-level driven changes in shelf denitrification. This contrast of demonstrates the shift in N cycle dynamics between these distinct time periods (PPT vs the late Pleistocene) and their respective climate states (see lines 162-258 and 261-289 of the revised manuscript).

66. Similarly, although also on modern sediments, the role of shelf-derived phosphorus as an important control on N fixation has also been previously evaluated using foram-d15N by Auderset et al., 2024 (Paleoceanography and Paleoclimatology).

*Auderset et al. (2024) demonstrated the influence of shelf-derived phosphorus on N fixation during the late Quaternary, using FB-$\delta^{15}N$ records and focusing on orbital-scale variability over the last 160 kyr. Our study extends this approach to a period of both global warmth (the late Pliocene) and the onset of large Northern Hemisphere glaciations, which makes it an ideal test case for understanding how the nitrogen cycle operates under very different background climate states. Importantly, according to Auderset et al., during the late Quaternary, N_2 fixation variability is mainly tied to **precession paced changes in upwelling** as well as shelf phosphorus supply. In contrast, our data provides evidence that the dominant pacing over the PPT is obliquity, and the mechanism is explicitly tied to sea-level driven shelf exposure and benthic denitrification, **not upwelling**. This means that the same basic process (shelf exposure driven phosphorus supply changes) operates differently depending on background climate and glacial boundary conditions.*

Finally, our main findings are that the PPT had overall lower N_2 fixation (linked to reduced global denitrification under a warm climate) and that superimposed on this were obliquity-paced fluctuations driven by sea-level change. This dual perspective, combining million-year trends with orbital-scale variability, available owing to the high resolution of our records, has not been done before with FB-$\delta^{15}N$. This demonstrates that the influence of shelf access P on N_2 fixation is climate-state dependent, and this insight into N cycle dynamic is not captured in late Quaternary studies and provides a new perspective on how the N cycle responds to major climate transitions.

All these points are now better clarified in the revised manuscript (see lines 200-258).

67. I am inclined to think the manuscript would benefit from revisions that more clearly highlight the novel insights from examining this time period specifically, particularly the shelf modelling, which to my knowledge has not been undertaken in previous studies.

Corrected. We now clearly highlight the novelty of our findings from examining this particular time period and of the shelf percentage modelling approach.

68. The authors mention in the abstract that the Pliocene is a geologic analog for future warming however this is not discussed in any detail within the manuscript;

See our response to comments #64-65. We have now revised this part and get back to it in the conclusions as well (see lines 73-83, 265-269 and 286-289).

69. what about the findings here are relevant to the future? If none, this should be removed from the abstract.

We have clarified this in lines 286-289 of the revised manuscript.

70. Alternatively/in addition, as the authors present the data from the Pacific cores within their figure 2, it would be useful to add discussion about how this new Atlantic data fits within a broader global context through this major climate transition.

We thank the reviewer for this comment as this is one of the main implications of our paper. We have clarified this point in lines 178-183 of the revised version, so it is more emphasized for the reader.

71. Overall, the study is well designed and presented and I have no major concerns with the methods, results or conclusions,

We thank the reviewer again for highlighting the quality of our work.

72. but I think they need to better highlight the specific novelty of this work in the discussion for publication in Nature Communications. I therefore recommend for publication with minor revisions.

See our response to comments #64-66 above.

Other Comments:

73. I struggled a little with flow of the narrative where there are switches between date ranges and named periods (e.g. iNHG vs G10 vs ~2.8 Ma) – improved consistency between what is used in figures and text would be helpful.

74. For example Figure 2 and 4 have NHG written and then MIS G10 associated with the iNHG in the caption.

75. It would be helpful to someone who doesn't work specifically in this time period if the same terms were used throughout.

Corrected. We have now improved the consistency of the different name and period indications throughout the paper.

76. Consider adding the last paragraph of the Core Location section (lines 282-284) into the end of the introduction as this is a clearer explanation of how the sites link together.

We agree that the relationship between the two sites is important for readers to understand. In the revised version, we have added a concise summary of how the two cores complement each other to the end of the introduction (lines 112–122), while retaining the more detailed hydrographic and methodological description in the Materials and Methods section to avoid overburdening the main text.

77. Data from IODP sites 806, 849 and 1012 are presented in Figure 2, but are not discussed in the text. There is mention of 1012 in the introduction, but no discussion is made of those datasets in relation to the new data presented here, so it is not clear why they are included.

We've added an explanation of how the new data relates to these records and why they are included. The bulk $\delta^{15}N$ data from IODP 1012 was removed and Sites 806 and 849 data have been recently published. These data are important because they provide direct constraints on water column denitrification changes associated with the Pacific ODZs, the regions that host the largest share of global denitrification. These records are therefore important for placing our Atlantic results in a global framework. See lines 178-183 of the revised manuscript.

78. I think this panel of the figure should either be removed or if not, could be an interesting addition to discuss the new findings from Atlantic in the broader context of the MOC and how similar sea level declines and potential P influx in the Pacific basin may contribute to d15N data presented from other papers?

We agree that discussing these data in the context of the meridional overturning circulation (MOC) is an interesting point, but it is beyond the main scope of this paper. Nevertheless, we have added a short mention of this broader connection in the revised manuscript (lines 205-208), noting the MOC related connection between the Pacific, excess P pathways and Atlantic upwelling and N_2 fixation.

79. Line 289: The authors indicate there is good correlation between G. ruber and Tssac (which looks to be mostly true from Fig S4). However, Straub et al., (2013), as cited by authors, indicates that there is variation in the species-specific offset between G.ruber and G. sacculifer during glacials and climate transitions in their FB-d15N study on the same site. Similarly in Ren et al., (2012), it states: "The δ15N of G. ruber is slightly lower than the other species…this difference is generally greatest between 16 and 25 ka…roughly similar to what has been observed in the Caribbean, where the maximal species difference also occurs at the LGM [Ren et al., 2009]." Could the authors comment on whether this is observed in their data, for example if they split their current Figure S4 data into glacial and interglacial datasets, does this alter the correlation significantly?

We thank the reviewer for raising this important point and agree it is appropriate to test whether the G. ruber–T. sacculifer offset in our Site 999 data differs between glacial and interglacial intervals rather than assuming a single fixed offset.

Accordingly, we divided Site 999 Fb-$\delta^{15}N$ data into glacial and interglacial subsets using MIS-based boundaries (Lisiecki and Raymo, 2005) and compared the two species' $\delta^{15}N$ values. The two species remain strongly correlated in both climate states ($r^2 = 0.64$, $p = 5.4 \times 10^{-14}$ for glacials; $r^2 = 0.49$, $p = 1.6 \times 10^{-7}$ for interglacials), with similar mean offsets of 0.81 ± 0.22‰ and 0.78 ± 0.25‰, respectively. Neither the t-test ($p > 0.1$) nor the bootstrap estimates show a significant difference in the species offsets between the two groups, meaning, the inter-species offset is stable across glacial interglacial conditions, supporting our use of a combined $\delta^{15}N$ record. Full details and figures are provided in the revised Materials and Methods section and in the Supplementary Information figures (See 'FB-$\delta^{15}N$ offset analysis between G.ruber and T.sacculifer at ODP Site 999' subsection of the revised Materials and methods, new Fig. S5 and Table S4).

80. In the modern, my understanding is that Tsacc lives slightly lower in the water column than G. ruber and therefore could be exposed to a more enriched N, as well as potentially calcifying later in the season (which could also enrich N signal). Could the authors comment on the potenthis and whether it could contribute to the higher values in this authors PPT record compared to the 160ka record which averages G. ruber and Tssac?

We agree that T. sacculifer typically calcifies slightly deeper and later in the season than G. ruber, which could in principle expose it to more ^{15}N-enriched nitrate. However, both our data and previous work at Site 999 (Straub et al., 2013) indicate that the offset between the two species is small and stable through time. In addition, as stated in the previous comment's response, G. ruber and T. sacculifer $\delta^{15}N$ values are strongly correlated ($r^2 = 0.64$, $p = 5.4 \times 10\text{-}14$ for glacial intervals; $r^2 = 0.49$, $p = 1.6 \times 10\text{-}7$ for interglacial intervals), and the mean offset between species (~0.8‰) does not differ significantly

between climate states (t-test $p > 0.1$). These results are now added to the manuscript (See 'FB-δ15N offset analysis between G.ruber and T.sacculifer at ODP Site 999' section of the revised Materials and methods of the revised manuscript) and confirm that the interspecies $\delta^{15}N$ difference is consistent. These results demonstrate that the higher $\delta^{15}N$ values observed during the Pliocene genuinely reflect changes in the nitrogen cycle.

81. Lines 169-178: Authors discuss a lack of correlation between the FB-d15N and dust but do not appear to undertake this test statistically (as they have done for the SST). Could similar or even more simple correlative tests be used to confirm this more objectively?

We thank the reviewer for their suggestion. We have now added a similar analysis with the dust records and show that there in no significant correlation between these two records that could point to dust derived Fe as a driver for N_2 fixation changes at the WTA See Fig. S10 of the revised manuscript.

Minor Comments:

82. Line 37: Add (N) after nitrogen?

Added. See line 36 of the revised manuscript.

83. Line 45: typo "reduced" rather than "reduce"

Corrected. See line 44 of the revised manuscript.

84. Line 64-64: low latitude with and without hyphen used

Corrected. See lines 69-71 of the revised manuscript.

85. Line 78: add reference(s)?

Added. See line 84 of the revised manuscript.

86. Line 82: ODZ already defined

Corrected. See line 87 of the revised manuscript.

87. Line 116: Authors suggest elevation of ~2 ‰ but that the mean shifts from 5.4 to 4.5 ‰ (0.9 ‰ difference). Could the author clarify where they got 2 ‰ from?

We have clarified this in lines 129-130 of the revised manuscript.

88. Line 117-120: Cited Fig 2b, should be 2a?

Corrected. See line 134 of the revised manuscript.

89. Line 120: Notable increase not drop?

Corrected. See line 134 of the revised manuscript.

90. Line 118: Glacial-interglacial variability is discussed and cited to Fig 2, but this is not clear on the figure, I think in part as the eye is drawn to the smoothing spline (which is helpful). I wonder whether citing to Figure 4 where the glacial bands are highlighted would be more useful.

Corrected. We've changed the order of figures so it matches the description in the text. See Figs 3, 4 of the revised manuscript.

91. Line 132: the NTC part of FB-$\delta^{15}N_{NTC}$ notation is not used in the figure, figure caption or consistently in discussion (e.g. line 162). Perhaps remove?

We retained the FB-$\delta^{15}N_{NTC}$ notation in the text for clarity but noted that the figures use a simplified label (FB-$\delta^{15}N$) to maintain consistency across sites displayed on shared axes. See lines 145-147 of the revised manuscript.

92. Fig 2: panel b is cited before panel a, should these be flipped?

Corrected throughout the revised manuscript.

93. Fig S1. Caption typo – duplicated "on the"

Corrected. See now Fig. S3 caption of the revised manuscript.

94. Current references to SI S4 which I don't think has been included in this submission.

Corrected. SI S1-S3 are now referenced throughout the revised manuscript.

References

Altabet, M.A., 2007. Constraints on oceanic N balance/imbalance from sedimentary15N records. Biogeosciences 4, 75–86. https://doi.org/10.5194/bg-4-75-2007

Auderset, A., Fripiat, F., Creel, R.C., Oesch, L., Studer, A.S., Repschläger, J., Hathorne, E., Vonhof, H., Schiebel, R., Gordon, L., Lawrence, K., Ren, H.A., Haug, G.H., Sigman, D.M., Martínez-García, A., 2024. Sea Level Modulation of Atlantic Nitrogen Fixation Over Glacial Cycles. Paleoceanogr. Paleoclimatology 39, 1–21. https://doi.org/10.1029/2024PA004878

Brandt, P., Alory, G., Awo, F.M., Dengler, M., Djakouré, S., Anicet, R., Koungue, I., Jouanno, J., Körner, M., Roch, M., Rouault, M., 2023. Physical processes and biological productivity in the upwelling regions of the tropical Atlantic 581–601.

Fripiat, F., Sigman, D.M., Martínez-García, A., Marconi, D., Ai, X.E., Auderset, A., Fawcett, S.E., Moretti, S., Studer, A.S., Haug, G.H., 2023. The Impact of Incomplete Nutrient Consumption in the Southern Ocean on Global Mean Ocean Nitrate δ15N. Global Biogeochem. Cycles 37, 1–25. https://doi.org/10.1029/2022GB007442

Gruber, N., 2008. The Marine Nitrogen Cycle: Overview and Challenges. Nitrogen Mar. Environ. 1–50. https://doi.org/10.1016/B978-0-12-372522-6.00001-3

Jung, J., Duprey, N.N., Foreman, A.D., Olivo, J.P.D., Pellio, C., Ryu, Y., Murphy, E.L., Romshoo, B., Kersting, D.K., Cardoso, G.O., Wald, T., Fripiat, F., Jimenez, C., Gischler, E., Montagna, P., Treinen-crespo, C., Carriquiry, J., Ong, M.R., Goodkin, N.F., Guppy, R., Aardema, H., Slagter, H., Heins, L., Angelis, I.H. De, Bieler, A.L., Yehudai, M., Noël, T.P., James, K., Scholz, D., Hu, C., Barnes, B.B., Pozzer, A., Pöhlker, C., Lelieveld, J., Pöschl, U., Vonhof, H., Haug, G.H., Schiebel, R., Sigman, D.M., Martínez-garcía, A., 2025. Equatorial upwelling of phosphorus drives Atlantic N 2 fixation and Sargassum blooms. Nat. Geosci. https://doi.org/10.1038/s41561-025-01812-2

Lawrence, K.T., Sigman, D.M., Herbert, T.D., Riihimaki, C.A., Bolton, C.T., Martinez-Garcia, A., Rosell-Mele, A., Haug, G.H., 2013. Time-transgressive North Atlantic productivity changes upon Northern Hemisphere glaciation. Paleoceanography 28, 740–751. https://doi.org/10.1002/2013PA002546

Lisiecki, L.E., Raymo, M.E., 2005. A Pliocene-Pleistocene stack of 57 globally distributed benthic d18O records. Paleoceanography 20, 1–17. https://doi.org/10.1029/2004PA001071

Marconi, D., Sigman, D.M., Casciotti, K.L., Campbell, E.C., Alexandra Weigand, M., Fawcett, S.E., Knapp, A.N., Rafter, P.A., Ward, B.B., Haug, G.H., 2017. Tropical Dominance of N2 Fixation in the North Atlantic Ocean. Global Biogeochem. Cycles 31, 1608–1623. https://doi.org/10.1002/2016GB005613

Rafter, P.A., Farmer, J.R., Martínez-García, A., Ravelo, A.C., Karnauskas, K.B., Batista, F.C., Bernasconi, S.M., Ren, H., Auderset, A., Haug, G.H., Sigman, D.M., 2025. Persistent eastern equatorial Pacific Ocean upwelling since the warm Pliocene. Science (80-.). 390. https://doi.org/10.1126/science.ads8720

Ren, H., Sigman, D.M., Martínez-García, A., Anderson, R.F., Chen, M. Te, Ravelo, A.C., Straub, M., Wong, G.T.F., Haug, G.H., 2017. Impact of glacial/interglacial sea level change on the ocean nitrogen cycle. Proc. Natl. Acad. Sci. U. S. A. 114, E6759–E6766. https://doi.org/10.1073/pnas.1701315114

Ren, H., Sigman, D.M., Meckler, A.N., Plessen, B., Robinson, R.S., Rosenthal, Y., Haug, G.H., 2009. Foraminiferal Isotope Evidence of Reduced Nitrogen Fixation in the Ice Age Atlantic Ocean. Science (80-.). 244, 244–248. https://doi.org/10.1126/science.1165787

Ren, H., Sigman, D.M., Thunell, R.C., Prokopenko, M.G., 2012. Nitrogen isotopic composition of planktonic foraminifera from the modern ocean and recent sediments. Limnol. Oceanogr. 57, 1011–1024. https://doi.org/10.4319/lo.2012.57.4.1011

Sigman, D.M., Fripiat, F., 2019. Nitrogen isotopes in the ocean, 3rd ed, Encyclopedia of Ocean Sciences. Elsevier Ltd. https://doi.org/10.1016/B978-0-12-409548-9.11605-7

Smart, S.M., Ren, H., Fawcett, S.E., Schiebel, R., Conte, M., Rafter, P.A., Ellis, K.K., Weigand, M.A., Oleynik, S., Haug, G.H., Sigman, D.M., 2018. Ground-truthing the planktic foraminifer-bound nitrogen isotope paleo-proxy in the Sargasso Sea. Geochim. Cosmochim. Acta 235, 463–482. https://doi.org/10.1016/j.gca.2018.05.023

Straub, M., Sigman, D.M., Ren, H., Martínez-García, A., Meckler, A.N., Hain, M.P., Haug, G.H., 2013. Changes in North Atlantic nitrogen fixation controlled by ocean circulation. Nature 501, 200–203. https://doi.org/10.1038/nature12397

REVIEWER COMMENTS

Reviewer #1 (Remarks to the Author):

I reiterate that I agree with the data interpretation of the authors and acknowledge the potential contribution of the work. I still find the revised version not clearly organized, and at places arguments are not coherent.

1. Introduction:

Information regarding N2 fixation and denitrification is mixed. Given that the key finding of the paper is about N2 fixation, I highly recommend gathering all the scattered pieces of information about N2 fixation, put them together, and clarify the gap of our knowledge regarding this process.

2. Section "Drivers of WTA N2 fixation since the Pliocene":

The interpretation of the site 662 record that is driven by global water-column denitrification is mixed with the interpretation of the d15N offset between Sites 999 and 662 that is driven by local N2 fixation.

We thank the reviewer for pointing this out. This section is now revised such that the interpretations about site 662 as representing a global signal and the $\delta^{15}N$ offset as driven by regional N_2 fixation are now clearly separated.

3. I am also surprised not to be able to find a statement like "less excess P due to weaker global water-column denitrification led to lowered N2 fixation in the WTA" in the paragraph from L175-189 that connects the 2 observations from the dataset. I strongly recommend the authors using their data to explain these points separately and improve the flow of reasoning here.

We have added a sentence stating this in the discussion, conclusions and Fig. 5 caption. See lines 212-215, 290-293 and 744-746 of the revised manuscript.

Detailed comments

4. L42-44: better to specify that this process is observed locally in the WTA.

Done. See line 45 of the revised manuscript.

5. L86-91: flow here can be improved. You discussed water-column denitrification and then sediment denitrification and finally came back to water-column denitrification.

Done. See lines 78-94 of the revised manuscript.

6. L116: do we know what percentage of global N2 fixation occurs in the WTA? If yes, it is worth mentioning.

Although a precise global percentage is not available, the tropical North Atlantic hosts the vast majority (~90%) of basin-wide N_2 fixation north of 11°S, identifying the region as a major center of marine N_2 fixation (Marconi et al., 2017). We have added this detail for clarification. See lines 120-122 of the revised manuscript.

7. L128: what is this "shorter" timescale compared to?

Revised. See line 132 of the revised version.

8. L130: No need to add "(hereafter T. sacculifer)". Genus names are shortened after the first appearance conventionally.

Revised. See line 134 of the revised version.

9. L151 move "throughout the PPT" after "dutertrei".

Revised. See lines 157-158 of the revised version.

10. L154: standard deviation of the interspecies offset should be shown, and such an error should be incorporated to the derived thermocline d15N.

Added. See line 160 and Materials and Methods section of the revised version.

11. Also, how is this interspecies 15N difference compared to those observed in previous work?

Added. See lines 396-417 of the revised version.

12. L160-1: If Site 999 d15N is lower than site 662, they are not similar. Better show averages with standard deviations.

Corrected. See lines 160-163 of the revised version.

13. L170-173: Only a higher d15N at site 662 can indicate that d15N supply to the tropical Atlantic was higher during the Pliocene.

Corrected. See lines 175-181 of the revised version.

14. L173-174: The observation that the d15N of nitrate supplied to the Tropical Atlantic was higher during the Pliocene does not support lower N2 fixation there.

Clarified. See lines 178-181 of the revised version.

15. L183: More explanation is needed here regarding the possible contribution of sedimentary denitrification to excess P production globally. If water column denitrification is associated with a d15N fractionation of ~-20 permil and the thermocline d15N is ~-6 permeil, global sedimentary denitrification should be more important for the denitrification and thus excess P production.

We have added more details about this in the following section and here chose to focus on the evidence we have for decreased water-column denitrification. See line 218-229 of the revised version.

16. L187-189: What are "these observations"? This sentence seems to be saying previous data can demonstrate that Atlantic N2 fixation was lowered during the Late Pliocene, which is not what the authors meant I assume.

Revised. See lines 197-201 of the revised version.

17. L207-209: this sentence is vague.

Revised. See line 219-221 of the revised version.

18. L211: While I agree that denitrification in the Pacific produces high-excess-P thermocline waters that can raise the global average thermocline excess P, I do not think you can track "(high-excess-P) Pacific-sourced waters entering the Atlantic via the Southern Ocean". This is not supported by modern hydrographic data.

Clarified. See line 221-229 of the revised version.

19. L211-213: this piece of information is also needed in the previous section on the overall Pliocene N2 fixation changes.

To maintain the flow of the previous paragraph we chose to keep the focus around evidence for decreased water column denitrification and added more explanation in the current paragraph. If the editor believes that this change should be implemented here we will change it. See lines 225-229 of the revised version.

20. L214-216: this sentence would work better if you rephrase it to something like "the upwelling control on N2 fixation can be supported by the relationships of d15N with alkenone MAR ".

Revised. See lines 230-232 of the revised version.

21. L225: change "lowest" to "lower than interglacials".

Done. See line 242 of the revised version.

22. L233: rephrase "rich in excess P"

Done. See lines 247-248 of the revised version.

23. L247-249: The observation that sea level controlled N2 fixation in SCS and tropical Atlantic during the late Pleistocene cannot be used to prove that such a process occur during the PPT in these regions or in the WTA.

We agree and therefore rephrased this sentence. See lines 261-264 of the revised version.

24. L279: remove "and potentially global". The observation is regional.

Done. See lines 283-286 of the revised version.

25. L286-289: similar to a previous comment, I wonder how the global sedimentary denitrification would change and how it would affect global excess P and thus N2 fixation.

We agree that changes in global sedimentary denitrification could influence excess P and N_2 fixation. Because this is beyond the scope of the present study, we revised the text to acknowledge this uncertainty and softened the wording of the global implication. See lines 291-296 of the revised version.

26. L301-304: specify this occurs in the Atlantic.

Done. See lines 308 of the revised version.

27. L358: this is Materials and Methods section.

Corrected. See lines 362 of the revised version.

28. Fig. 2: In the legend, it is better to provide the species used for the records, especially for Site 662 record that is derived from 2 species.

Done. See lines 684-686 in the revised version.

29. Standard errors for d15N measurements and derived thermocline d15N at 662 need to be shown.

30. Done. See Fig. 2 in the revised version.

31. It would be helpful to highlight PPT in the figure.

Done. See Fig. 2 in the revised version.

32. Fig. 3a,4a: specify these are benthic d18O data.

Done. See Figs. 3a and 4a in the revised version.

33. Fig. 5: In d, better to say early Pleistocene glaicals to be consistent.

Done. See Fig. 5d in the revised version.

34. Double check processes in panels c &d.

Done. See Fig. 5c and 5d in the revised version.

35. S1: this section or a simplified version of it should be included the Methods.

Done. The text was moved to the Materials and Methods section. See lines 418-448 of the revised manuscript.

Reviewer #2 (Remarks to the Author):

36. Overview and general recommendation

I thank the authors for their careful consideration of my review and thoughtful responses. This version of the manuscript is much more coherent and highlights differences with existing work more clearly. I commend them for their additional work and data analysis to address some of my comments and particularly like the addition of Figure 5 which nicely visualizes the key findings. I have highlighted a few additional, minor comments, which I do not feel will significantly change the manuscript, but might enhance these small areas. I would recommend this manuscript for publication.

We thank the reviewer for the thoughtful acknowledgment and kind words.

37. Paragraph at Line 248: As you outline how records from the late Pleistocene reinforce your results, would there be any relevance in discussing changes related to the Mid-Pleistocene transition, and closure of the Bering Strait/exposure of the Beringian continental shelf, which are proposed to have suppressed North Pacific upwelling (through enhanced NPIW) in glacial periods and caused elevated d15N records in the Bering Sea and subarctic Pacific? Would your mechanism of shelf exposure and P supply fit with this as well [making this a more global hypothesis]? There are several bulk d15N records from IODP Expedition 323 that cover this interval by authors including Knudson, Ravelo, Kender and Worne, as well as older published records from ODP Site 882. Maybe not relevant/beyond scope, but just a thought.

We thank the reviewer for this insightful suggestion. We agree that exploring potential links to Mid-Pleistocene transition changes in the North Pacific and Bering Strait dynamics would be an interesting avenue for future work. However, such analysis would require additional regional datasets and is beyond the scope of our present study, which focuses on tropical Atlantic N_2 fixation dynamics. We therefore did not modify the manuscript text.

38. Lines 75-79: A very long and clunky sentence, could this be edited/split into two?

Done. See lines 70-74 in the revised version.

39. Fig S4. Could the authors put the r2 and p values onto the panels for each plot to visualize and evidence that there is no correlation. While it does not look like there is for most panels, it would be interesting to know the correlation between FB-d15N and N content of G.ruber which looks to be slightly positive? If so, some explanation of how this impacts results would be needed.

Done. See Fig. S4 in the revised version.

40. Fig S7. Color differences between light and dark teal/pink are very hard to see. Could the authors change the color/symbology to show a clearer difference between this study and the referenced studies, to make sure to consider the requirements for visually impaired readers who may struggle with the current color format.

Colors/symbols have been changed. See Fig. S7 in the revised version.

41. Also, the caption implies both panels of data have been smoothed but I don’t think the second panel of teal/pink data has been smoothed here, so caption may need to be adjusted, or the smoothed data replaced onto the figure.

The caption was corrected. See Fig. S7 in the revised version.

REVIEWER COMMENTS

Reviewer #1 (Remarks to the Author):

1. I still think that the data and science presented in this manuscript is high-quality, valid, and interesting.

We appreciate the reviewer's positive feedback on our manuscript.

2. But I did not see substantial improvement in this version from the last one, and thus cannot support publication of the work in the current form, for the same reasons I gave in the last round of review.

Per the reviewer's suggestion we have carefully re edited the manuscript to address all the concerns raised.

3. I do not think my pervious comments on the Section "Drivers of WTA N2 fixation since the Pliocene" has been addressed. Here is my previous comment:

"The interpretation of the site 662 record that is driven by global water-column denitrification is mixed with the interpretation of the d15N offset between Sites 999 and 662 that is driven by local N2 fixation."
In this current version, the arguments for the use of Dd15N between Sites 662 and 999 for inferring N2 fixation changes are still not well laid out.

For example, the authors state that "the WTA-EEA FB-δ15N differences (ΔFB-δ15N; Fig. S9) to reflect the strength of N2 fixation in the tropical North Atlantic" on L178. A key premise of this statement is that "Regions where the main pycnocline outcrops, such as at Site 66253, provide an opportunity to monitor its nitrate δ15N, for comparison with records such as that of Site 999.", which does not appear until L196. Then L199 is repeating the statement at L178.

We have reedited this section again such that we first discuss results from Site 662 (see lines 181-186 of the revised manuscript), compare it with Pacific data to establish that it reflects global pycnocline changes (see lines 186-189 of the revised manuscript). Then, we move on to discuss the interpretation of the FB-δ^{15}N data and include the data from Site 999 (lines 189-206) and finally Sites 999-662 $\Delta\delta^{15}$N values (see lines 206-215 of the revised manuscript).

4. Also, the authors confusingly mentioned the excess P change at L189-90 but only explain the potential role of excess P to N2 fixation at L200, and then at L202 introduce

potential drivers of N2 fixation (including excess P).
In the last review, I "recommend the authors using their data to explain these points separately". I reiterate this recommendation. It would be much clearer if you first discuss results from Site 662, compare it with Pacific data to establish that it reflects global thermocline changes (including site 999). Then you move on to discuss what the Dd15N tell us.

We have reedited this section again such that we first explain the potential role of excess P in maintaining N_2 fixation (see lines 195-202 of the revised version) and then mention excess P changes inferred from our data and their implications for N_2 fixation (see lines 204-215 of the revised manuscript).

5. While I acknowledge that language should not be used to judge the quality of the manuscript, I am listing the large number of grammatical errors and confusing sentences in this 3rd version I read as a major concern. These errors make it difficult to follow the author's arguments. I encourage the authors to carefully proofread the manuscript, including the material and method section.

We thank the reviewer for thoroughly reading through our manuscript. In the revised version we have carefully proofread the text and corrected all typos and grammar mistakes, including the materials and methods.

Here is an incomplete list of sentences need revision due to grammatical errors

6. L65: remove "biological" before "sources"
Corrected. See line 64 of the revised text.

7. L72-4
Corrected. See line 70-72 of the revised text.

8. L81-3
Corrected. See line 80-82 of the revised text.

9. L83: in in
Corrected. See line 82 of the revised text

10. L85 rephrase 14N-nitrate
Corrected. See line 83-87 of the revised text

11. L85-6
Revised. See line 83-87 of the revised text

12.L 98 change "and" to "because"
Done. See line 99 of the revised text

13. L103-5
Rephrased. See line 103-106 of the revised text

14. L120 NEARLY
Corrected. See line 120 of the revised text

15. L165
Corrected. See line 156-170 of the revised text

16. L223: Unclear what "they" refer to.
Revised. See line 237 of the revised text

17. L223: Southern-sourced and ventilated in the Southern Ocean, repetitive.
Revised. See line 236-239 of the revised text

Minor comments.
18. L68: 3-3.3 Ma is the Mid Pliocene Warm Period. In L118, you are referring 3.6 Ma as Late Pliocene.
Revised. See line 117 of the revised text

19. L112-4: This is repeating L59-63 and L89-91
Removed. See line 109-115 of the revised text

20. L120 and 123: giving locations of these cores in the main text is the common practice.
Added. See lines 19 and 123 of the revised text

21. L125: Do the authors mean the pycnocline is below the thermocline at this site? Perhaps stick to thermocline or pycnocline throughout the manuscript (see again eg, L158, L330), if it is not important to distinguish these two.

We thank the reviewer for this comment. In the low-latitude open ocean settings of our study, the thermocline and pycnocline are essentially co-located, as temperature is the dominant control on density stratification. We use "thermocline-dwelling" when describing foraminiferal depth habitats, following standard usage in the foraminifera literature, and "pycnocline" when referring to the nitrate pool these species record. We have revised the manuscript throughout for consistency. See line 124 and throughout the revised text.

22. L126: this is misleading. Only Site 999 covers 1 ma of time.
Revised. See line 126-128 of the revised text

23. L141-2: Firstly, I am not sure what you are comparing the PPT IG d15N with, the average of the last 160 ka or the IG values of the last 160 ka?

Clarified. See line 141-145 of the revised text

24. Secondly, this is also a strange start to describe your G-IG scale results. It is better to focus on your data first then move on to comparison with published data.

Done. We first describe the 999 data and only then compare it to what was published from Site 999 in order to build the interpretive framework for this Site without disturbing the flow. Then we describe Site 662 data and then compare it to the published data from the last 160 ka and also compare the data between the two sites. See line 141-177 of the revised text

25. Thirdly, it should be mentioned that the glacial d15N are higher somewhere in this paragraph.

Revised. See line 141-145 of the revised text

26. L164: I don't disagree with the authors that d15N of these two species can be used to reflect the thermocline signal, now that I am reading this manuscript the third time. But this is a point that will puzzle many in the field that know about the habitats of these species. Providing some explanations for your readers here would be great.

Done. See line 170-177 of the revised text

27. L165: Check the numbers here. The numbers are inconsistent with your arguments. This sentence is also unclearly written.

The numbers have been checked and this paragraph was also revised to address the previous comment. See line 170-177 of the revised text

28. L177: Not sure this is true. The smoothed PPT d15N level at Site 999 is comparable to that during the last 50 ka according to Fig.2

Revised. See line 203-206 of the revised text

29. L225-229: this is relevant information but seems to be awkwardly positioned here.
We have moved these sentences to the intro and revised this paragraph, see lines 216-229 of the revised text.

30. Fig 2: The duration of PPT is not correctly shown
Corrected. We have removed the wording label. The arrows now show when the Pliocene ends and the Pleistocene begins. See Fig. 2 and all relevant figures of the revised version of the manuscript.

Reviewer #2 (Remarks to the Author):

31. I am satisfied that authors have responded to all comments.

We appreciate the reviewer's thoughtful and thorough evaluation of our study.
